# A study on the correlation between TCM syndrome types, TCM symptoms and myocardial injury markers in patients with coronary heart disease

**DOI:** 10.3389/fcvm.2025.1669239

**Published:** 2026-01-12

**Authors:** Zhou Mi, Li Jieyun, Xiao Xin ‘ang, Lim Jiekee, Xu Zhaoxia

**Affiliations:** 1 School of Traditional Chinese Medicine, Shanghai University of Traditional Chinese Medicine, Shanghai, China; 2 Shanghai Key Laboratory of Health Identification and Evaluation, Shanghai, China; 3 Shanghai Guangde Traditional Chinese Medicine Clinic, Shanghai, China

**Keywords:** coronary heart disease, discriminant analysis, markers of myocardial injury, predictive model, TCM syndrome types

## Abstract

**Background:**

Coronary heart disease (CHD) is the leading cause of death from cardiovascular diseases. Previous studies related to traditional Chinese medicine (TCM) mostly lacked objective basis for TCM syndrome types, which may affect the accuracy of syndrome differentiation.

**Objective:**

To explore the distribution pattern of TCM syndrome types in patients with CHD, analyze its correlation with TCM symptoms and myocardial injury markers.

**Method:**

This study adopted a cross-sectional research design. A total of 1,503 patients with CHD from January 2023 to January 2025 were included. The clinical and TCM four diagnostic data of the selected patients were systematically collected, aiming to analyze the association between TCM syndrome types and TCM symptoms as well as myocardial injury markers, and to construct and verify the predictive model of TCM syndrome types.

**Result:**

A total of 1435 patients were included and grouped according to the syndrome types in TCM. The main types were 295 cases (20.5%) of phlegm blocking the heart meridian syndrome, 289 cases (20.1%) of qi and Yin deficiency syndrome, 257 cases (17.9%) of heart and kidney Yin deficiency syndrome, 199 cases (13.8%) of qi deficiency and blood stasis syndrome, 177 cases (12.3%) of qi stagnation and blood stasis syndrome, and 186 cases (12.9%) of heart blood stasis obstruction syndrome. BNP and NT-proBNP were statistically significant differences among different TCM syndrome types (*P* < 0.05). Further, unordered multi-class regression analysis was used to compare the differences in TCM symptom indicators among different TCM syndrome types. Meaningful statistical results and TCM symptoms were combined for discriminant analysis of TCM syndrome types (coincidence rate of discrimination = 62.8%), and machine learning models (LGBM, XGBoost, etc.) were used to construct TCM syndrome type prediction models. Ultimately, the best-performing model LGBM (validation set accuracy = 72.51%) was selected and SHAP was used to explain the contribution of the model.

**Conclusion:**

The combination of TCM symptoms and myocardial injury markers can be used to distinguish and predict the syndrome types of patients.

## 1 Introduction

The “Summary of the 2023 China Cardiovascular Health and Disease Report” (1) shows that the current number of people suffering from CHD is 11.39 million. Globally, cardiovascular diseases (CVDs) are the leading cause of morbidity and mortality, incurring significant social and economic costs. Among them, CHD is the leading cause of death from cardiovascular diseases worldwide (2). Although percutaneous coronary intervention (PCI) and drug therapy have improved clinical outcomes, the management of CHD still faces challenges in terms of risk stratification and personalized treatment, especially the combination of TCM and evidence-based Western medicine (3).

The TCM syndrome differentiation of CHD, such as “blood stasis blocking the heart” and “phlegm-dampness blocking the meridians”, has long been used to guide treatment strategies. However, their clinical application is limited by subjective diagnostic criteria relying on symptomatology, and there is a lack of biomarkers to verify the syndrome differentiation (4). This ambiguity may lead to inconsistent treatment responses and hinder the standardization of TCM protocols in modern cardiology.

More and more evidence indicates that biomarkers of myocardial injury, such as lactate dehydrogenase (LDH), cardiac troponin (cTnT), and N-terminal pro-brain natriuretic peptide (NT-proBNP) (5), not only reflect myocardial pathophysiology but may also be related to TCM syndrome types. For instance, elevated LDH is associated with tissue hypoxia and microcirculation dysfunction, which may be consistent with the pathogenesis of “phlegm and blood stasis” in TCM (6); Similarly, NT-proBNP, a marker of ventricular wall pressure, may reflect the “Yang deficiency and water retention” syndrome characterized by fluid retention and autonomic nerve imbalance (7); cTnT has the highest level in “Qi deficiency and blood stasis syndrome”, followed by “Yang deficiency and water retention syndrome” and “Qi and Yin deficiency syndrome”, which may be related to myocardial microcirculation disorders associated with CHD (8). Despite these hypotheses, systematic studies on the biomolecular characteristics of TCM syndrome types of CHD remain scarce. Existing studies often have small sample sizes, high heterogeneity in syndrome classification, and insufficient statistical methods to handle high-dimensional data. Therefore, strictly designed studies are needed to clarify syndrome-specific biomarkers.

Here, we conducted a cross-sectional analysis of 1,435 patients with CHD confirmed by coronary angiography, aiming to explore the distribution characteristics of TCM syndrome types in patients with CHD and analyze the correlation between myocardial injury markers (e.g., LDH, cTnT, N-proBNP, etc.) and TCM syndrome types was further explored. The predictive value of these biomarkers for differentiating TCM syndrome types was further investigated. Finally, the relevant factors were screened through multiple disordered logistic regression analysis. The discriminant and predictive model of TCM syndrome types for CHD were finally established, and Shapley Additive Explanatory Values (SHAP) was used to explain the contribution of the predictive model.

**Figure 1:**
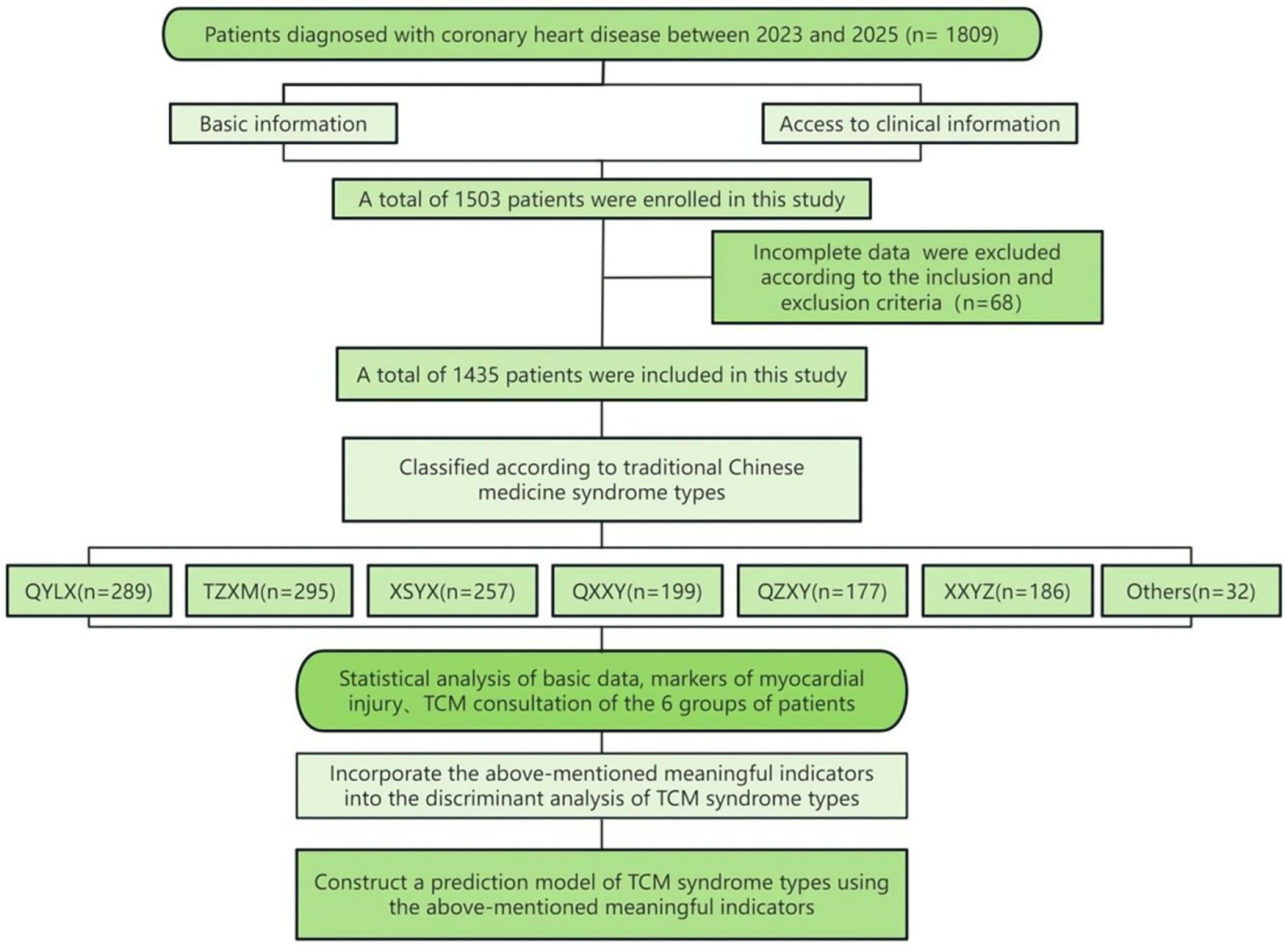
Flowchart.

Our research aims to bridge the gap between TCM syndrome differentiation and objective biomarkers, enhance the accuracy of syndrome differentiation, and provide a framework for precise medical treatment of CHD, with the expectation of offering ideas for the objectification and standardization of TCM syndrome type research on CHD. The flowchart is shown in Figure 1.

## 2 Materials and methods

### 2.1 Clinical data

#### 2.1.1 Source of research subjects

This study included 1,435 patients with CHD who were hospitalized in Shuguang Hospital Affiliated to Shanghai University of Traditional Chinese Medicine, Yueyang Hospital Affiliated to Shanghai University of Traditional Chinese Medicine, Shanghai Yangpu District Hospital of Traditional Chinese Medicine, Shanghai Antu Branch of Yangpu District Central Hospital, and Shanghai Jiading District Central Hospital from January 2023 to January 2025.

#### 2.1.2 Diagnostic criteria

The diagnostic criteria for CHD in Western medicine: If coronary angiography (CAG) reveals that the diameter of the subepicardial coronary artery is narrowed by more than 50%, and the patient has typical angina pectoris symptoms, and there is evidence of ischemic changes on the electrocardiogram (such as ST-segment elevation or lowering, T-wave inversion, etc.), CHD can be diagnosed (9).There are two major types of CHD in the guidelines, namely Chronic coronary syndrome (CCS) and acute coronary syndrome (ACS). The former is further divided into stable angina pectoris, ischemic cardiomyopathy and latent cardiomyopathy. The latter is divided into Unstable angina (UA) and ST-segment elevation myocardial infarction (STEMI) and non-ST-segment elevation myocardial infarction (NSTEMI).

The criteria for TCM diagnosis and treatment of CHD in our research group: According to the “Guidelines for the Diagnosis and Treatment of Stable Angina Pectoris in Coronary Heart Disease with Traditional Chinese Medicine” (10), patients with CHD are classified into eight syndrome types: Qi and Yin deficiency syndrome, phlegm blocking the heart meridian syndrome, heart and kidney Yin deficiency syndrome, Qi stagnation and blood stasis syndrome, heart blood stasis obstruction syndrome, Qi deficiency and blood stasis syndrome, Yang Qi deficiency and decline syndrome, and Yin cold stagnation syndrome. (The clinical manifestations are as Supplementary Appendix AI).

#### 2.1.3 Inclusion criteria

① It meets the Western medical diagnostic criteria for CHD; ② Age: 35–85 years old. ③ Informed consent of the patient; ④ The clinical literature and materials are complete and accurate.

#### 2.1.4 Exclusion criteria

① Patients with acute or chronic nephritis, urinary tract infections, hypertrophic cardiomyopathy, trauma, surgery, acute infectious diseases and severe liver or kidney dysfunction; ② Pregnant and lactating women, and those with mental disorders; ③ Subjects who voluntarily propose to withdraw; ④ In cases where serious complications occur or the condition deteriorates during the research process and emergency measures need to be taken; ⑤ Incomplete clinical data or those that do not conform to the data collection process.

#### 2.1.5 Research methods

This study adopted a cross-sectional research design and included 1,435 patients with CHD. General information, disease diagnosis, TCM symptoms, and myocardial injury markers of the patients were collected.

The TCM symptoms were collected based on the TCM consultation information collection form for CHD made by our research group (see Supplementary Appendix AII). This scale includes a total of 275 symptoms and signs, including general conditions (such as gender, age, height, weight, past medical history and family history, etc.), specialized inquiries (palpitations, chest tightness, chest pain, shortness of breath/shortness of breath, etc.), systemic inquiries (chills and fever, sweating, head, body, chest and abdomen, diet, sleep, defecation and urination, etc.) and other conditions (exercise status, information on the patient's tongue, face, pulse, etc.). As this syndrome type analysis was grouped based on TCM syndrome types, the items were screened in combination with clinical experience and TCM theories, and the symptoms and signs that have reference value for diagnosis were retained, totaling 141.

This research has been reviewed and approved by the Ethics Committee of Shanghai University of Traditional Chinese Medicine, with the approval number: 2023-3-10-08-07 (see Supplementary Appendix AIII). At the same time, it is confirmed that informed consent has been obtained from the research participants and that compliance with the guidelines outlined in the Helsinki Declaration has been confirmed.

#### 2.1.6 General information

The patient's age, gender, Body Mass Index (BMI), TCM symptoms and other information, as well as past medical history and risk factors, such as: history of hypertension, history of diabetes, history of hyperlipidemia, history of hyperuricemia, history of cerebral infarction, history of chronic gastritis, history of malignant tumors, history of smoking, history of drinking, etc.

#### 2.1.7 Markers of myocardial injury

Creatine kinase isoenzyme (CK-MB), creatine kinase (CK), lactate dehydrogenase (LDH), aspartate aminotransferase (AST), troponin I (cTnI), troponin T (cTnT), hypersensitive troponin (hs-cTnI), myoglobin (MYO), heart-type fatty acid binding protein (H-FABP), brain Natriuretic peptide (BNP), N-terminal pro-B-type natriuretic peptide (NT-proBNP).

#### 2.1.8 Quality control

All the clinical data required for this research were sourced from the hospital. The data entry was completed by two people (Zhou Mi and Xiao Xin ‘ang). They carefully checked the data needed for this research, strictly followed the established diagnostic criteria for TCM syndrome types (10) to conduct syndrome differentiation for patients with CHD, and used a unified TCM consultation information collection form for CHD to enter the data. After the data entry was completed, the two people carefully checked and proofread. If there are any objections, please have the third chief physician of TCM (Xu Zhaoxia) make the ruling.

#### 2.1.9 Statistical methods

Data analysis was conducted using SPSS 27.0 statistical software. Counting data are expressed in absolute numbers and relative numbers (%). Measurement data conforming to the normal distribution were expressed as (z ± s), and those not conforming to the normal distribution were expressed as M(P25, P75). The chi-square test was used for the comparison between groups of count data, and a two-sided *P* < 0.05 was statistically significant. One-way analysis of variance (ANOVA) or Kruskal–Wallis H test was used for the comparison between groups of measurement data. A two-sided *P* < 0.05 was statistically significant.

The modeling plan of this research is divided into principal analysis and exploratory analysis. Among them, establishing a predictive model is the principal analysis, while unordered multiple logistic regression and discriminant analysis are exploratory analyses.

Unordered multiple logistic regression was used to analyze the symptom indicators of TCM. The above baseline data with statistical significance, myocardial injury markers, and symptom indicators of TCM were analyzed for discriminant analysis and a discriminant analysis model was constructed. The prediction accuracy of each TCM syndrome type was calculated and a typical function classification graph was drawn. The included indicators were feature screened through the Boruta method, and finally, machine learning was used to construct a multi-classification prediction model for the main syndrome types. Machine learning algorithms can extract useful information from massive amounts of data and make objective and accurate predictions through algorithm models. We hope to analyze baseline data, myocardial injury markers and TCM symptoms through machine learning to more comprehensively assess their application value in predicting TCM syndrome types. The predictive capabilities of five different models (XGBoost, LGBM, Logistic, Random Forest and GBDT) were compared. The cross-validation method was adopted with 5 times and 88 random seeds. The accuracy was calculated, and the learning curve and confusion matrix heat map were drawn. During each modeling process, the dataset is randomly divided into a training set and a test set in a ratio of 8.5:1.5. The training set is used for model training, while the test set is used to verify model performance. The classification metrics used to evaluate the performance of model training and validation include accuracy, precision, recall, support and F1 Score.

To enhance the credibility of the model and facilitate its use by clinicians, it is necessary not only to report the prediction results but also to explain the model. In our study, we used SHAP for visual analysis. The SHAP value not only reveals the significance of features but also provides a quantitative indicator to evaluate the contribution of each feature to the prediction results, which enables us to accurately compare the impact of different baseline data, myocardial injury markers, and TCM symptoms on the model output results. This study will utilize the best machine learning algorithm for TCM syndrome types and use the Jizhi Analysis platform to plot the SHAP value of each sample, the vertical axis represents variables, with the higher the elevation, the greater the importance. The horizontal axis represents numerical values. Different colors in the graph represent different types of TCM syndromes, and the larger the area, the greater the importance of the variable to a certain syndrome type.

## 3 Results

### 3.1 Comparison of data on different TCM syndrome types in patients with CHD

#### 3.1.1 Distribution frequency of TCM syndrome types of CHD

**Figure 2:**
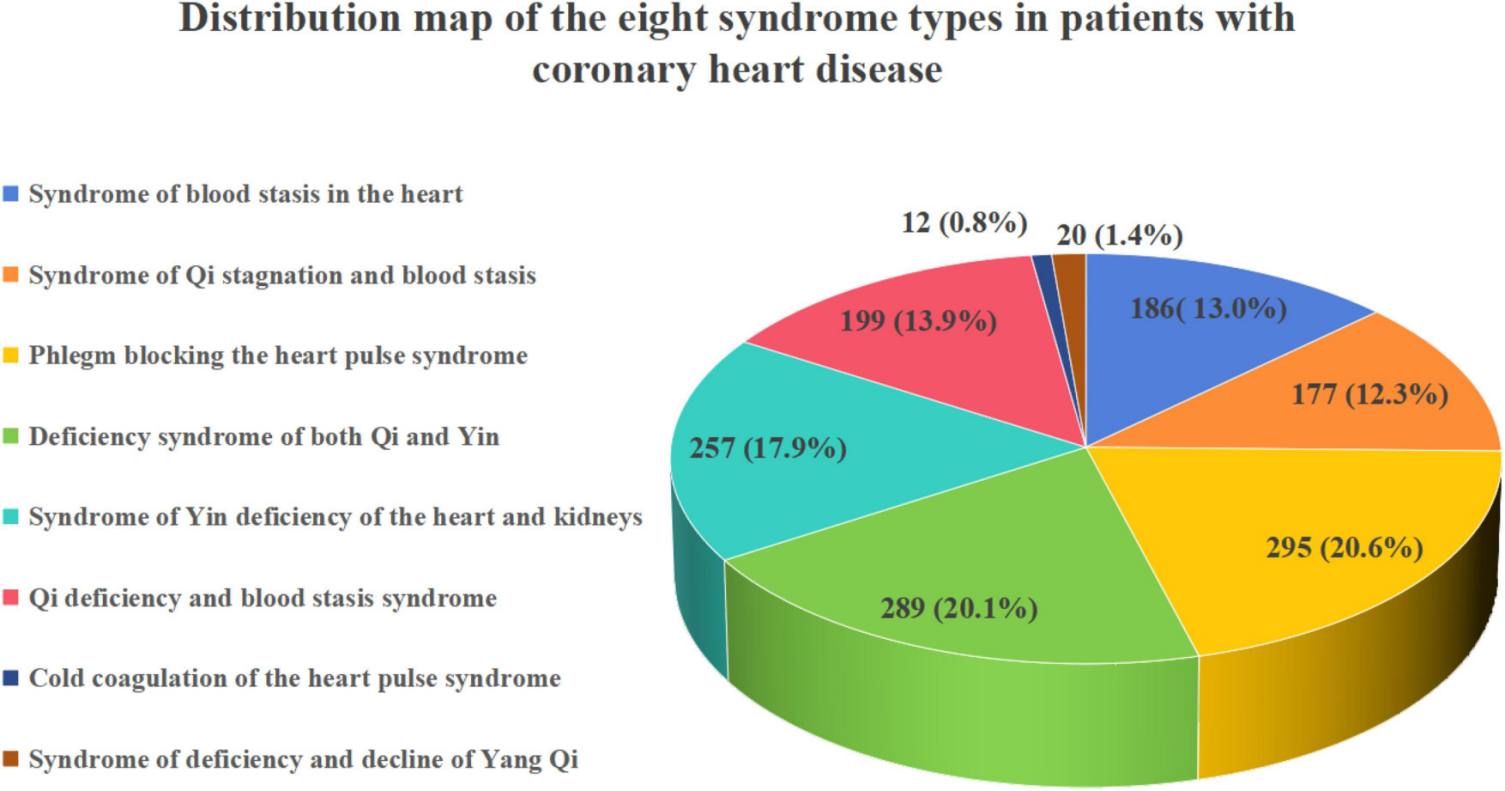
Distribution map of the 8 major syndrome types of CHD.

As shown in Figure 2, the results of this study indicate that the distribution of various syndrome types among 1,435 CHD patients is as follows: There were 295 cases (20.5%) of phlegm blocking the heart meridian syndrome, 289 cases (20.1%) of Qi and Yin deficiency syndrome, 257 cases (17.9%) of heart and kidney Yin deficiency syndrome, 199 cases (13.8%) of Qi deficiency and blood stasis syndrome, 177 cases (12.3%) of Qi stagnation and blood stasis syndrome, and 186 cases (12.9%) of heart blood stasis obstruction syndrome. The sample sizes of the other syndrome types were too small. A total of 32 cases (with 2.1% of the cases being Yang Qi deficiency syndrome and Yin cold stagnation syndrome), and the specific distribution is shown in Figure 2.

#### 3.1.2 Baseline data

This study statistically analyzed 1,403 cases of six major syndrome types with a relatively large number of syndrome types (phlegm blocking the heart meridian syndrome, Qi and Yin deficiency syndrome, heart and kidney Yin deficiency syndrome, Qi deficiency and blood stasis syndrome, Qi stagnation and blood stasis syndrome, and heart blood stasis blocking syndrome). Chi-square test and Kruskal–Wallis H test were used to statistically analyze the baseline differences among patients of the six syndrome types. The results showed that the differences in BMI, Pulmonary artery systolic pressure, disease course, White blood cell(WBC), High-density lipoprotein cholesterol(HDL) among the six syndrome types were statistically significant (*P* < 0.05), as detailed in Table 1.

**Table 1:** Baseline data analysis of patients with different TCM syndrome types of CHD.

Variable name		1 (*n* = 289)	2 (*n* = 177)	3 (*n* = 295)	4 (*n* = 257)	5 (*n* = 186)	6 (*n* = 199)	Z	*p*
Gneder, *n* (%)	Female	136 (47.059)	94 (53.107)	118 (40.000)	121 (47.082)	80 (43.011)	95 (47.739)	9.035	0.108
	Male	153 (52.941)	83 (46.893)	177 (60.000)	136 (52.918)	106 (56.989)	104 (52.261)		
Age		69.000 [61.000,75.000]	70.000 [64.000,76.000]	69.000 [62.000,76.000]	70.000 [63.000,76.000]	69.000 [64.000,74.000]	71.000 [65.000,75.000]	4.185	0.523
Systolic blood pressure (SBP)		130.000 [120.000,142.000]	130.000 [120.000,141.000]	130.000 [122.000,142.000]	127.000 [120.000,141.000]	133.000 [121.000,146.000]	133.000 [120.000,144.000]	7.829	0.166
(Diastolic blood pressure) DBP		78.000 [70.000,86.000]	77.000 [70.000,82.000]	78.000 [72.000,82.000]	77.000 [70.000,82.000]	77.000 [68.000,86.000]	78.000 [72.000,83.000]	4.061	0.541
Heart rate		76.000 [70.000,80.000]	75.000 [68.000,80.000]	77.000 [66.000,80.000]	76.000 [68.000,81.000]	75.000 [68.000,80.000]	75.000 [68.000,80.000]	1.596	0.902
BMI		24.035 [21.778,26.644]	24.035 [21.094,26.639]	25.381 [22.491,27.587]	24.280 [22.032,27.239]	24.088 [20.957,26.122]	23.438 [21.970,25.620]	22.368	<0.001
History of smoking, *n* (%)	0	200 (69.204)	110 (62.147)	174 (58.983)	164 (63.813)	115 (61.828)	131 (65.829)	7.485	0.187
	1	89 (30.796)	67 (37.853)	121 (41.017)	93 (36.187)	71 (38.172)	68 (34.171)		
History of alcohol consumption, *n* (%)	0	212 (73.356)	120 (67.797)	185 (62.712)	184 (71.595)	127 (68.280)	134 (67.337)	9.057	0.107
	1	77 (26.644)	57 (32.203)	110 (37.288)	73 (28.405)	59 (31.720)	65 (32.663)		
Hypertension, *n* (%)	0	77 (26.644)	53 (29.944)	100 (33.898)	73 (28.405)	71 (38.172)	62 (31.156)	9.083	0.106
	1	212 (73.356)	124 (70.056)	195 (66.102)	184 (71.595)	115 (61.828)	137 (68.844)		
Type 2 diabetes(T2DM), *n* (%)	0	196 (67.820)	116 (65.537)	211 (71.525)	175 (68.093)	120 (64.516)	144 (72.362)	4.799	0.441
	1	93 (32.180)	61 (34.463)	84 (28.475)	82 (31.907)	66 (35.484)	55 (27.638)		
Hyperlipidemia, *n* (%)	0	268 (92.734)	161 (90.960)	267 (90.508)	238 (92.607)	174 (93.548)	177 (88.945)	4.108	0.534
	1	21 (7.266)	16 (9.040)	28 (9.492)	19 (7.393)	12 (6.452)	22 (11.055)		
Hyperuricemia, *n* (%)	0	281 (97.232)	170 (96.045)	290 (98.305)	249 (96.887)	183 (98.387)	193 (96.985)	3.333	0.649
	1	8 (2.768)	7 (3.955)	5 (1.695)	8 (3.113)	3 (1.613)	6 (3.015)		
Cerebral infarction, *n* (%)	0	251 (86.851)	150 (84.746)	258 (87.458)	236 (91.829)	163 (87.634)	173 (86.935)	5.826	0.324
	1	38 (13.149)	27 (15.254)	37 (12.542)	21 (8.171)	23 (12.366)	26 (13.065)		
Chronic gastritis, *n* (%)	0	280 (96.886)	167 (94.350)	280 (94.915)	248 (96.498)	180 (96.774)	192 (96.482)	3.332	0.649
	1	9 (3.114)	10 (5.650)	15 (5.085)	9 (3.502)	6 (3.226)	7 (3.518)		
Gensini		40.000 [0.000,170.000]	30.000 [0.000,180.000]	40.000 [0.000,150.000]	37.500 [0.000,170.000]	60.000 [5.000,240.000]	37.000 [0.000,155.000]	7.566	0.182
Left ventricular ejection fraction(LVEF)		63.000 [58.000,67.000]	64.000 [61.000,68.000]	64.000 [61.000,67.000]	64.000 [60.000,67.000]	64.000 [62.000,67.000]	67.000 [67.000,68.000]	9.854	0.079
Pulmonary artery systolic pressure		54.000 [54.000,54.000]	49.000 [30.000,65.000]	38.000 [24.000,57.000]	50.000 [30.000,65.000]	42.000 [27.000,55.000]	46.000 [30.000,57.000]	22.482	<0.001
Course of the disease		1.000 [1.000,2.000]	1.000 [1.000,2.000]	1.000 [1.000,2.000]	1.000 [1.000,2.000]	1.000 [1.000,2.000]	2.000 [1.000,2.000]	14.830	0.011
Red blood cell (RBC)		4.252 [3.950,4.580]	4.398 [4.111,4.750]	4.290 [3.900,4.670]	4.270 [3.910,4.630]	4.310 [3.980,4.650]	4.375 [4.030,4.730]	15.167	0.010
WBC		6.000 [5.000,6.940]	6.100 [5.500,7.430]	6.040 [5.200,7.200]	6.109 [5.000,7.500]	6.110 [5.200,7.400]	6.160 [5.200,7.400]	3.442	0.632
Platelet		193.449 [168.000,229.000]	188.000 [152.000,233.000]	187.000 [155.000,229.000]	194.000 [166.000,230.000]	196.000 [157.000,229.000]	193.000 [166.000,229.000]	2.949	0.708
Total cholesterol (TC)		4.000 [3.280,4.630]	3.863 [3.250,4.690]	4.000 [3.280,4.900]	3.900 [3.320,4.870]	3.840 [3.260,4.670]	3.930 [3.280,4.800]	1.160	0.949
Low-density lipoprotein cholesterol (LDL)		2.100 [1.720,2.710]	2.046 [1.560,2.720]	2.250 [1.680,3.000]	2.129 [1.680,2.950]	2.240 [1.570,3.000]	2.082 [1.560,2.810]	6.255	0.282
HDL		1.060 [0.890,1.240]	1.080 [0.870,1.280]	1.120 [0.950,1.310]	1.110 [0.960,1.300]	1.040 [0.890,1.280]	1.030 [0.860,1.220]	13.984	0.016
TG (Triglyceride)		1.300 [0.970,1.744]	1.280 [0.900,1.890]	1.300 [1.030,1.790]	1.251 [0.950,1.712]	1.290 [0.960,1.950]	1.310 [0.960,1.850]	1.271	0.938

1 Qi and Yin deficiency syndrome.

2 Qi stagnation and blood stasis syndrome.

3 Phlegm turbidity obstruction syndrome.

4 Syndrome of Yin deficiency of heart and kidney.

5 Syndrome of blood stasis in the heart.

6 Qi deficiency and blood stasis syndrome.

#### 3.1.3 Comparison of myocardial injury markers in patients with CHD of different TCM syndrome types

The myocardial injury markers in different TCM syndrome types groups were compared. Table 2 below shows that there were statistically significant differences in BNP and NT-proBNP indicators (*P* < 0.05), while the remaining indicators had no statistical significance.

**Table 2:** Analysis of differences in myocardial injury markers among patients with different TCM syndrome types of CHD.

Variable name	1 (*n* = 289)	2 (*n* = 177)	3 (*n* = 295)	4 (*n* = 257)	5 (*n* = 186)	6 (*n* = 199)	*Z*	*p*
AST	21.877 [18.000,26.000]	21.000 [17.000,27.200]	23.700 [19.000,28.400]	21.000 [17.500,26.700]	21.000 [17.000,28.000]	23.000 [18.400,28.000]	6.410	0.268
CK	75.290 [61.000,99.000]	79.000 [61.000,95.944]	76.000 [53.000,99.000]	77.526 [64.000,99.468]	78.251 [67.000,97.116]	76.000 [58.000,99.000]	2.300	0.806
LDH	188.380 [168.000,214.309]	187.000 [161.139,212.000]	194.840 [170.000,224.000]	189.000 [173.000,215.630]	187.860 [170.000,211.000]	190.000 [172.000,224.000]	6.176	0.290
MYO	37.396 [30.454,48.278]	37.800 [31.601,48.792]	36.601 [28.400,48.000]	37.941 [31.298,50.066]	35.747 30.000,47.700]	37.547 [32.500,48.600]	4.760	0.446
CKMB	2.290 [1.460,6.000]	2.290 [1.310,6.000]	2.740 [1.510,6.000]	2.640 [1.510,6.050]	2.400 [1.450,5.000]	3.000 [1.620,6.000]	6.256	0.282
cTnT	0.017 [0.008,0.024]	0.017 [0.007,0.020]	0.016 [0.007,0.028]	0.020 [0.008,0.040]	0.020 [0.008,0.033]	0.019 [0.007,0.027]	7.350	0.196
BNP	107.657 [64.563,193.822]	116.849 [62.783,239.925]	113.167 [59.320,253.864]	121.819 [65.662,252.054]	114.580 [70.654,201.828]	145.940 [90.630,224.263]	11.731	0.039
NT-proBNP	250.400 [124.238,589.714]	255.600 [95.000,477.900]	223.203 [70.000,492.032]	265.400 [131.200,669.500]	249.060 [98.766,550.953]	286.686 [133.643,653.423]	11.761	0.038

1 Qi and Yin deficiency syndrome.

2 Qi stagnation and blood stasis syndrome.

3 Phlegm turbidity obstruction syndrome.

4 Syndrome of Yin deficiency of heart and kidney.

5 Syndrome of blood stasis in the heart.

6 Qi deficiency and blood stasis syndrome.

### 3.2 Discriminant analysis of different syndrome types based on baseline data, TCM symptoms, and myocardial injury markers

This study took the syndrome types of TCM as the dependent variable and used TCM symptom indicators such as palpitations, chest tightness, shortness of breath, and chest pain as independent variables for unordered multi-class logistic regression analysis. Due to the excessive number of symptom indicators in TCM, the baseline data with statistical differences in the above statistical results, the symptom indicators of TCM and the myocardial injury marker indicators (BNP, NT-proBNP) were discriminated and analyzed to construct the discriminant.The specific results of the unordered multi-class logistic regression analysis can be found in Supplementary Material Table S1.

A total of 32 indicators were ultimately included in the discriminant analysis, and a classification variable based on TCM syndrome types was established: BMI (X1), chest pain (X2), pain on the inner side of the shoulder and arm (X3), distending pain (chest) (X4), dull pain (chest) (X5), emotional distress induced (X6), alcohol or overeating induced (X7), rainy days induced (X8), relief after rest (X9), fatigue and reluctance to speak (X10), forgetfulness (X11), spontaneous sweating (X12), dizziness (X) 13) Dry eyes (X14), tinnitus (X15), distension in the flanks (X16), general body aches (X17), preference for cold drinks (X18), constipation (X19), frequent urination at night (X20), insomnia (X21), irritability (X22), anxiety/depression (X23), sallow complexion (X24), red tongue (X25), dark purple tongue (X) 26), plump tongue (X27), scanty coating (X28), mirror tongue (X29), taut pulse (X30), BNP (X31), NT-proBNP (X32) are the independent variable discriminant equations. The coefficients of the discriminant analysis classification function are shown in Table 3, as detailed below, Table 4 shows the dialectical results of the discriminant analysis:

1. Y1 = 0.834X1−0.259X2−0.473X3 + 1.024X4 + 0.269X5−1.108X6 +  1.542X7−2.257X8 + 3.626X9 + 2.902X10 + 0.780X11 + 2.182X12 +  2.248X13−0.627X14 + 1.022X15 + 0.200X16−0.294X17 − 0.204X18 + 1.018X19 + 0.918X20 + 1.116X21 + 0.416X22 + 0.940X23−0.971X24 + 2.423X25 + 1.929X26 + 0.896X27 + 1.584X28  + 1.424X29 + 1.669X30 + 0.001X31 + 0.000X32−16.569
2. Y2 = 0.816X1 + 3.641X2−1.619X3 + 3.649X4−2.808X5 + 4.844X6− 0.988X7−1.622X8 + 2.184X9 + 2.362X10 + 0.321X11 + 0.407X12 + 1.018X13 + 0.102X14 + 1.329X15 + 2.315X16 + 0.127X17− 0.142X18 + 0.550X19 + 0.836X20 + 0.576X21 + 2.513X22 +  3.401X23 + 1.443X24 + 2.518X25 + 2.814X26 + 0.798X27 +  3.173X28 + 4.565X29 + 2.857X30 + 0.001X31 + 0.000X32−19.664
3. Y3 = 0.857X1 + 0.558X2 + 0.850X3 + 1.411X4 + 2.773X5−1.115X6 +  3.257X7−0.703X8 + 2.649X9 + 2.425X10−0.062X11 + 0.690X12 +  1.108X13−0.194X14 + 1.537X15−0.303X16 + 2.592X17 + 1.199X18 +  0.879X19 + 0.675X20 + 0.726X21 + 0.610X22 + 1.618X23−0.592X24 + 2.749X25 + 2.370X26 + 1.166X27 + 1.532X28 +  1.285X29 + 1.339X30 + 0.001X31 + 0.000X32−17.243
4. Y4 = 0.881X1−0.248X2−0.454X3 + 1.864X4−0.173X5−1.280X6 +  1.974X7−1.038X8 + 2.611X9 + 2.578X10 + 1.380X11 + 0.563X12 +  0.982X13 + 1.448X14 + 2.342X15−0.236X16 + 0.211X17 +  0.107X18 + 0.634X19 + 0.448X20 + 2.751X21 + 1.008X22 +  1.480X23−0.728X24 + 3.728X25 + 2.681X26 + 0.599X27 +  2.462X28 + 3.141X29 + 1.315X30 + 0.001X31 + 0.000X32−18.938
5. Y5 = 0.812X1 + 6.156X2 + 3.451X3−1.957X4−4.657X5−0.614X6−1.055X7−0.434X8 + 2.181X9 + 2.582X10 + 1.303X11 + 0.735X12 + 0.607X13−0.324X14 + 2.050X15−0.058X16−0.405X17−0.348X18−0.105X19 + 0.546X20 + 0.543X21 + 0.095X22 + 1.226X23 + 0.718X24 + 2.543X25 + 2.818X26 + 0.546X27 + 1.290X28 + 3.107X29 + 1.504X30 + 0.001X31 + 0.000X32−18.338
6. Y6 = 0.821X1 + 2.865X2−0.656X3−0.408X4−0.556X5−0.847X6 +  1.027X7−1.828X8 + 3.640X9 + 5.227X10−0.687X11 + 1.389X12 +  1.243X13−0.433X14 + 1.426X15 + 1.006X16−0.161X17 − 0.400X18 + 0.701X19 + 0.657X20 + 0.449X21 + 0.442X22 + 1.634X23 +  3.922X24 + 3.203X25 + 4.258X26 + 3.180X27 + 1.217X28 + 2.027X29 + 1.091X30 + 0.001X31 + 0.000X32−18.305

**Table 3:** Coefficients of discriminant analysis classification function.

Variable name	Certificate type					
	1	2	3	4	5	6
X1BMI	0.834	0.816	0.857	0.881	0.812	0.821
X2Chest pain	−0.259	3.641	0.558	−0.248	6.156	2.865
X3Pain leads to the inner side of the shoulder and arm	−0.473	−1.619	0.850	−0.454	3.451	−0.656
X4Distending pain (chest)	1.024	3.649	1.411	1.864	−1.957	−0.408
X5Dull pain (chest)	0.269	−2.808	2.773	−0.173	−4.657	−0.556
X6Triggered by unfulfilled emotions	−1.108	4.844	−1.115	−1.280	−0.614	−0.847
X7Induced by excessive drinking or overeating	1.542	−0.988	3.257	1.974	−1.055	1.027
X8Induced by rainy days	−2.257	−1.622	−0.703	−1.038	−0.434	−1.828
X9Relieved after rest	3.626	2.184	2.649	2.611	2.181	3.640
X10Weak and reluctant to speak	2.902	2.362	2.425	2.578	2.582	5.227
X11Forgetfulness	0.780	0.321	−0.062	1.380	1.303	−0.687
X12Spontaneous sweating	2.182	0.407	0.690	0.563	0.735	1.389
X13Dizziness	2.248	1.018	1.108	0.982	0.607	1.243
X14Dry eyes	−0.627	0.102	−0.194	1.448	−0.324	−0.433
X15Tinnitus	1.022	1.329	1.537	2.342	2.050	1.426
X16Fullness in the costal region	0.200	2.315	−0.303	−0.236	−0.058	1.006
X17Aches all over the body	−0.294	0.127	2.592	0.211	−0.405	−0.161
X18Like cold drinks	−0.204	−0.142	1.199	0.107	−0.348	−0.400
X19Constipation	1.018	0.550	0.879	0.634	−0.105	0.701
X20Frequent urination at night	0.918	0.836	0.675	0.448	0.546	0.657
X21Insomnia	1.116	0.576	0.726	2.751	0.543	0.449
X22Quick-tempered and irritable	0.416	2.513	0.610	1.008	0.095	0.442
X23Anxiety/Depression	0.940	3.401	1.618	1.480	1.226	1.634
X24Sallow complexion	−0.971	1.443	−0.592	−0.728	0.718	3.922
X25Red tongue	2.423	2.518	2.749	3.728	2.543	3.203
X26Purple Dark Tongue	1.929	2.814	2.370	2.681	2.818	4.258
X27Fat tongue	0.896	0.798	1.166	0.599	0.546	3.180
X28Less moss	1.584	3.173	1.532	2.462	1.290	1.217
X29Mirror tongue	1.424	4.565	1.285	3.141	3.107	2.027
X30Pulse Strings	1.669	2.857	1.339	1.315	1.504	1.091
X31BNP	0.001	0.001	0.001	0.001	0.001	0.001
X32NT-proBNP	0.000	0.000	0.000	0.000	0.000	0.000
(Constant)	−16.569	−19.664	−17.243	−18.938	−18.338	−18.305

1 Qi and Yin deficiency syndrome.

2 Qi stagnation and blood stasis syndrome.

3 Phlegm turbidity obstruction syndrome.

4 Syndrome of Yin deficiency of heart and kidney.

5 Syndrome of blood stasis in the heart.

6 Qi deficiency and blood stasis syndrome.

**Table 4:** Dialectical results of discriminant analysis.

Classification result^a^^,^^c^									
Certificate type		Certificate type	Predict the information of the group members						
			1	2	3	4	5	6	Total
Original	Count (%)	1	192 (66.4)	8 (2.8)	11 (3.8)	33 (11.4)	16 (5.5)	29 (10.0)	289 (100)
		2	13 (7.3)	108 (61.0)	7 (4.0)	13 (7.3)	29 (16.4)	7 (4.0)	177 (100)
		3	39 (13.2)	14 (4.7)	160 (54.2)	36 (12.2)	27 (9.2)	19 (6.4)	295 (100)
		4	35 (13.6)	10 (3.9)	19 (7.4)	159 (61.9)	23 (8.9)	11 (4.3)	257 (100)
		5	3 (1.6)	10 (5.4)	5 (2.7)	6 (3.2)	158 (84.9)	4 (2.2)	186 (100)
		6	23 (11.6)	10 (5.1)	24 (12.1)	8 (4.0)	29 (14.6)	104 (52.5)	198 (100)
Cross-validation^b^	Count (%)	1	184 (63.7)	9 (3.1)	15 (5.2)	39 (13.5)	16 (5.5)	26 (9.0)	289 (100)
		2	16 (9.0)	107 (60.5)	6 (3.4)	9 (5.1)	30 (16.9)	9 (5.1)	177 (100)
		3	48 (16.3)	14 (4.7)	154 (52.2)	36 (12.2)	27 (9.2)	16 (5.4)	295 (100)
		4	40 (15.6)	11 (4.3)	24 (9.3)	150 (58.4)	20 (7.8)	12 (4.7)	257 (100)
		5	4 (2.2)	11 (5.9)	5 (2.7)	6 (3.2)	151 (81.2)	9 (4.8)	186 (100)
		6	30 (15.2)	12 (6.1)	30 (15.2)	13 (6.6)	31 (15.7)	82 (41.4)	198 (100)

1 Qi and Yin deficiency syndrome.

2 Qi stagnation and blood stasis syndrome.

3 Phlegm turbidity obstruction syndrome.

4 Syndrome of Yin deficiency of heart and kidney.

5 Syndrome of blood stasis in the heart.

6 Qi deficiency and blood stasis syndrome.

^a^ 62.8% of the original grouped cases were classified correctly.

^b^ Cross-validation is only conducted for individual cases in the analysis. In cross-validation, each case is classified by the functions derived from all cases other than that one.

^c^ The 59.1% of cross-validated grouped cases were correctly classified.

**Figure 3:**
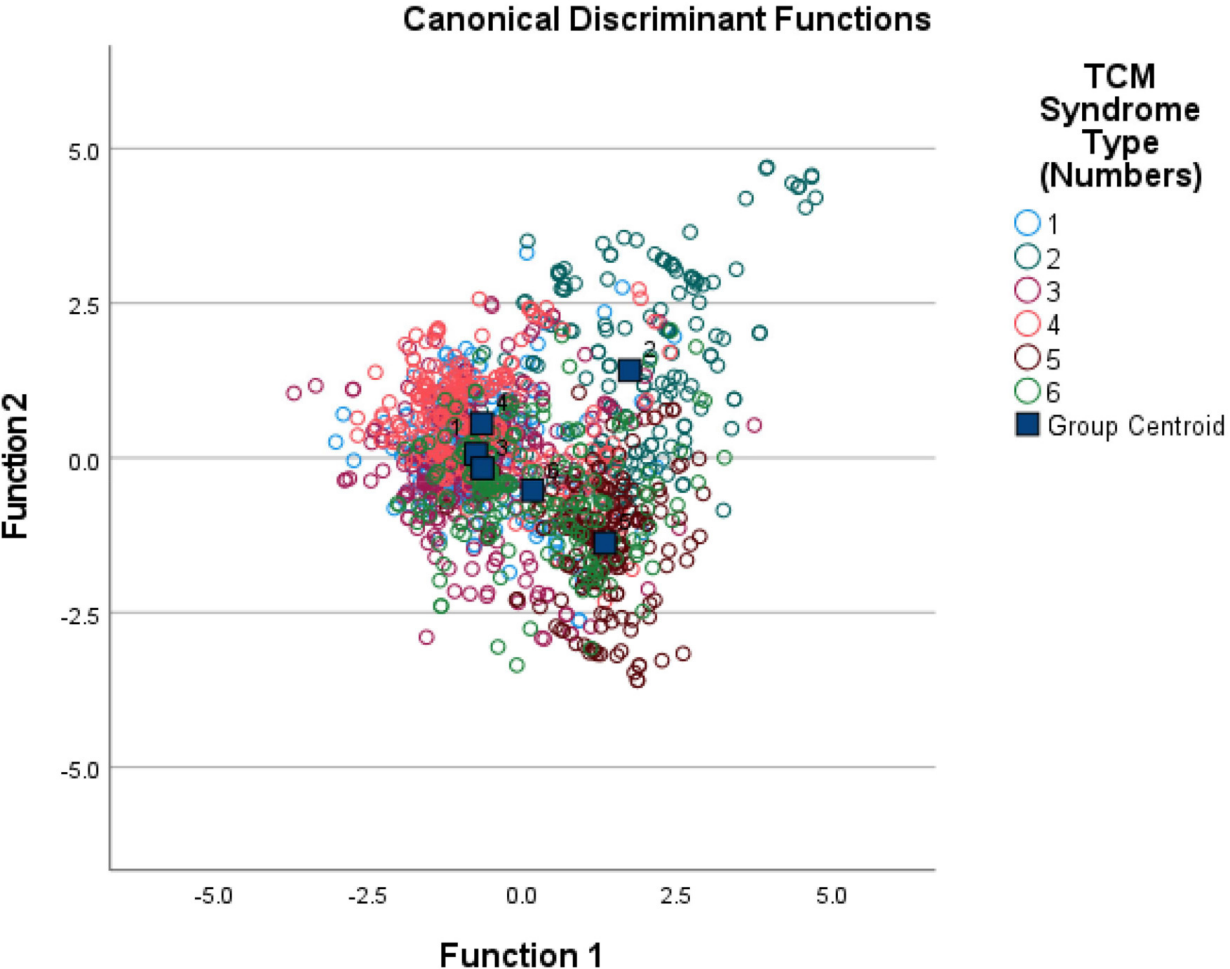
Overall classification diagram of canonical functions.

The predictive results of the discriminant analysis function show that the overall coincidence rate of discriminating the TCM syndrome types of CHD patients using TCM symptom indicators in this study is 62.8%, as detailed in Figure 3. As shown in the results of Table 5, the specific accuracy rates of each TCM classification are as follows: phlegm blocking the heart meridian syndrome (54.2%), Qi and Yin deficiency syndrome (66.4%), heart and kidney Yin deficiency syndrome (61.9%), Qi deficiency and blood stasis syndrome (52.5%), Qi stagnation and blood stasis syndrome (61.0%), and heart blood stasis blocking syndrome (84.9%).

**Table 5:** Analysis table of five model validation sets (baseline + TCM symptoms + myocardial injury markers).

Model	Name	Precision	Recall	f1-score	Support
Logistic	1	1.0	1.0	1.0	13.0
	2	1.0	1.0	1.0	10.0
	3	1.0	1.0	1.0	18.0
	4	1.0	1.0	1.0	18.0
	5	1.0	1.0	1.0	12.0
	6	1.0	1.0	1.0	16.0
	Accuracy	1.0	1.0	1.0	1.0
	Macro avg	1.0	1.0	1.0	87.0
	Weighted avg	1.0	1.0	1.0	87.0
XGBoost	1	0.617	0.659	0.637	13.0
	2	0.762	0.762	0.762	13.0
	3	0.711	0.711	0.711	10.0
	4	0.7	0.622	0.659	16.0
	5	0.649	0.8	0.716	19.0
	6	0.643	0.545	0.59	16.0
	Accuracy	0.673	0.673	0.673	0.673
	Macro avg	0.68	0.683	0.679	87.0
	Weighted avg	0.675	0.673	0.671	87.0
LGBM	1	0.808	0.7	0.75	14.0
	2	0.895	0.81	0.85	6.0
	3	0.581	0.781	0.667	17.0
	4	0.774	0.615	0.686	22.0
	5	0.7	0.875	0.778	17.0
	6	0.667	0.686	0.676	11.0
	Accuracy	0.725	0.725	0.725	0.725
	Macro avg	0.737	0.744	0.734	87.0
	Weighted avg	0.74	0.725	0.726	87.0
Random Forest	1	0.765	0.929	0.839	14.0
	2	0.375	0.5	0.429	6.0
	3	0.625	0.588	0.606	17.0
	4	0.85	0.773	0.81	22.0
	5	0.7	0.824	0.757	17.0
	6	0.667	0.364	0.471	11.0
	Accuracy	0.701	0.701	0.701	0.701
	Macro avg	0.664	0.663	0.652	87.0
	Weighted avg	0.707	0.701	0.695	87.0
GBDT	1	0.765	0.929	0.839	14.0
	2	0.25	0.333	0.286	6.0
	3	0.667	0.588	0.625	17.0
	4	0.833	0.682	0.75	22.0
	5	0.684	0.765	0.722	17.0
	6	0.5	0.455	0.476	11.0
	Accuracy	0.667	0.667	0.667	0.667
	Macro avg	0.616	0.625	0.616	87.0
	Weighted avg	0.678	0.667	0.668	87.0

1 Qi and Yin deficiency syndrome.

2 Qi stagnation and blood stasis syndrome.

3 Phlegm turbidity obstruction syndrome.

4 Syndrome of Yin deficiency of heart and kidney.

5 Syndrome of blood stasis in the heart.

6 Qi deficiency and blood stasis syndrome.

### 3.3 Construction and interpretation of predictive models for different syndrome types of CHD patients based on baseline data, TCM symptoms, and myocardial injury markers

**Figure 4:**
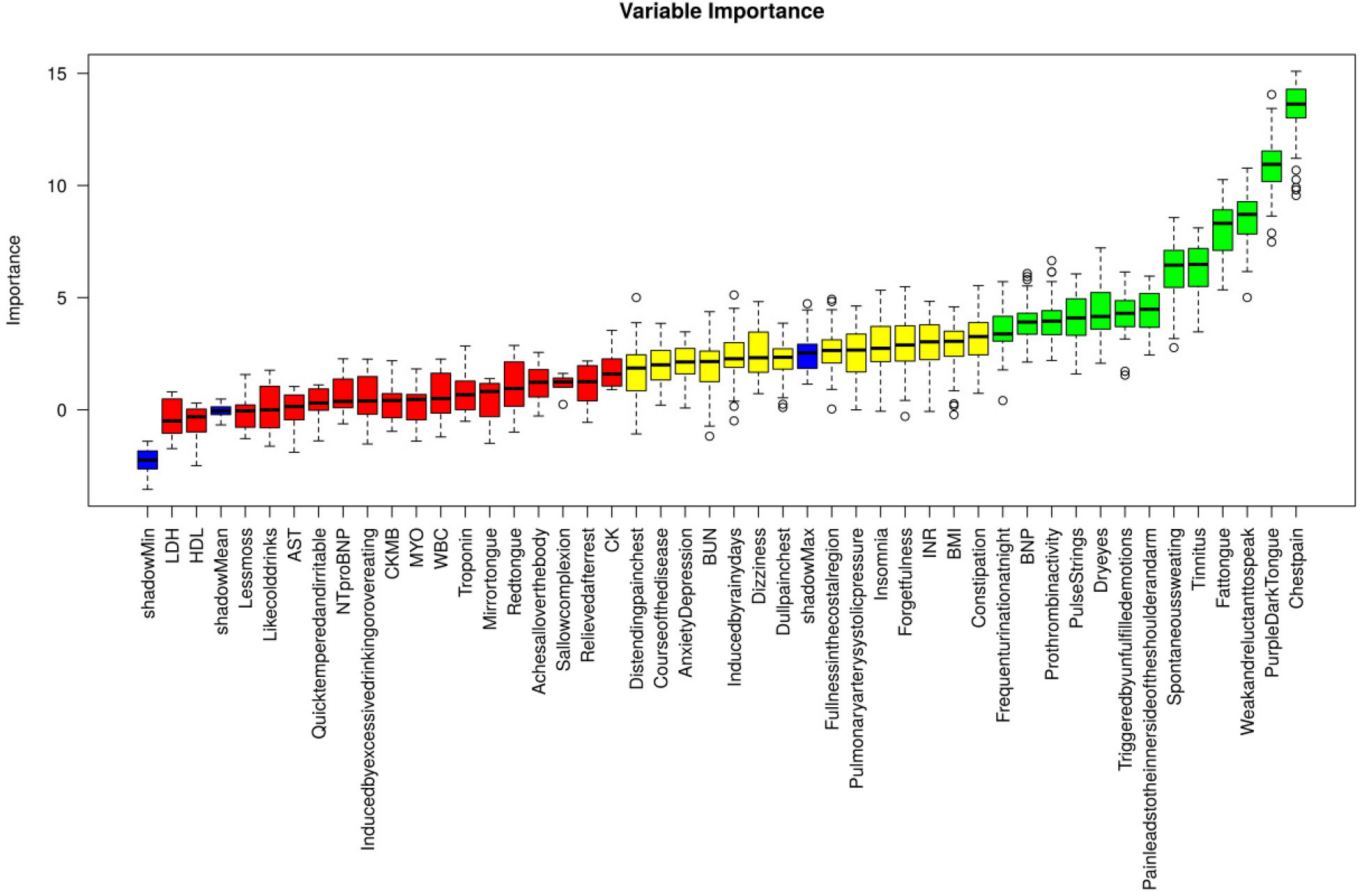
Feature screening of boruta.

The above baseline data with statistical significance, TCM symptoms, myocardial injury markers and other indicators were subjected to Boruta feature screening, and the following indicators were obtained and could be included in the final model establishment (Figure 4): Chest pain, Pain leads to the inner side of the shoulder and arm, Triggered by unfulfilled emotions, Weak and reluctant to speak, Spontaneous sweating, Dry eyes, Tinnitus, Frequent urination at night, Purple Dark Tongue, Fat tongue, Pulse Strings, BNP, Prothrombinactivity, BMI, Distending pain chest, Dull pain chest, Induced by rainy days, Forget fulness, Dizziness, Fullness in the costal region, Constipation, Insomnia, Anxiety Depression, BUN, INR, Course of the disease, Pulmonary artery systolic pressure.

Using 5-fold cross-validation, multiple machine learning models (Logistic, XGBoost, LGBM, Random Forest, GBDT) were used to predict the six syndrome types. Firstly, baseline data and TCM symptoms were included to establish the prediction model, training set and validation set (Supplementary Tables S2, S3), and then myocardial injury markers were included on this basis to establish the model. Compare the results of the two models. The results showed that the accuracy of the model established by incorporating baseline data, TCM symptoms and myocardial injury markers was significantly better than that of the model established by incorporating baseline data and TCM symptoms.

**Figure 5:**
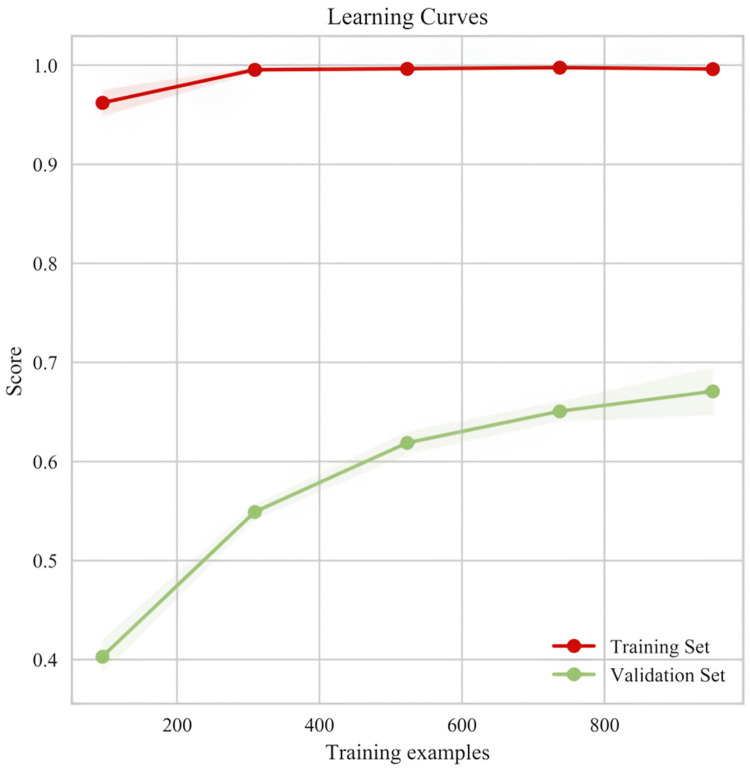
Learning curves of the training set and validation set of the LGBM model.

**Figure 6:**
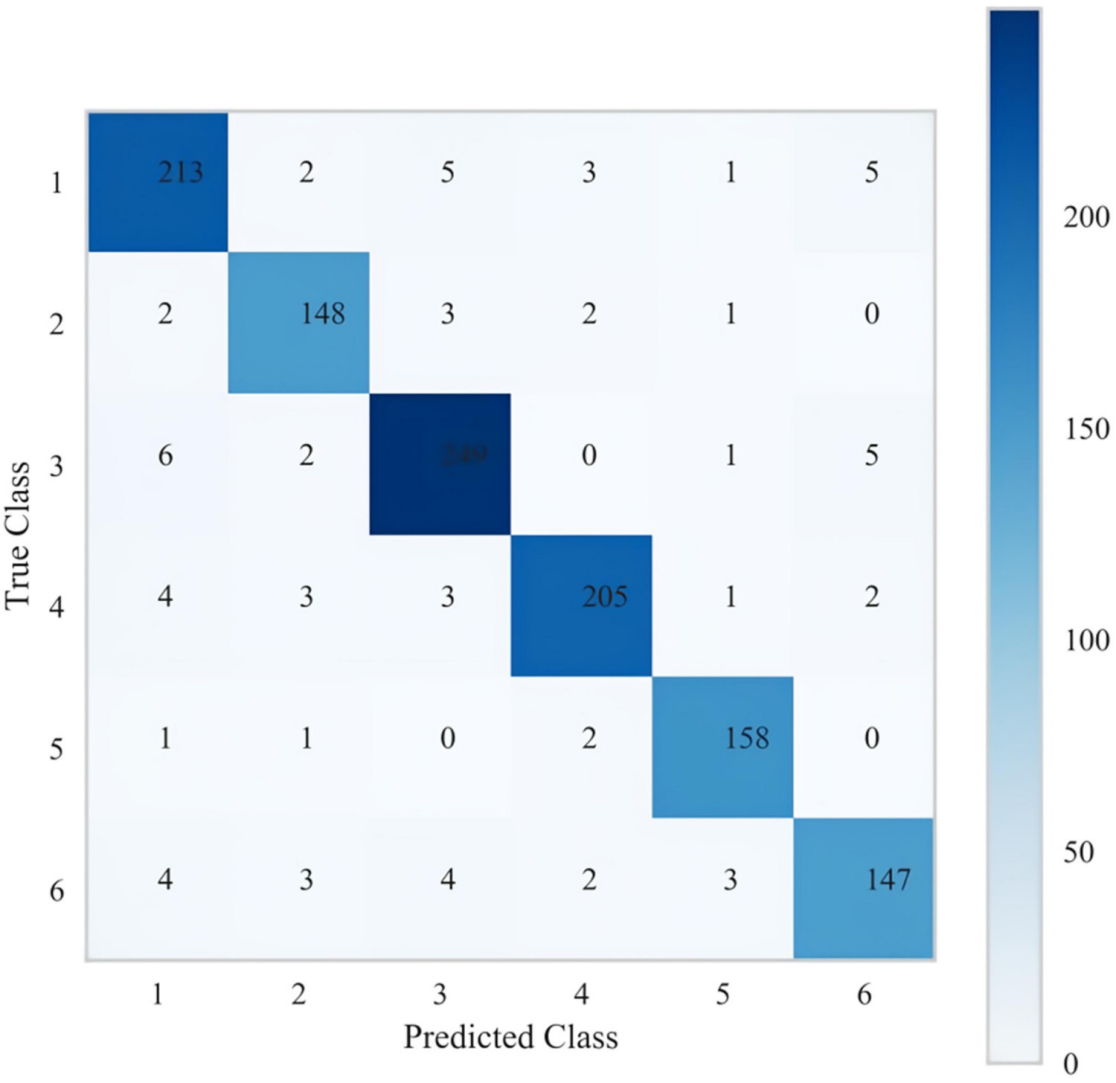
Heat map of the confusion matrix of the LGBM model training set.

**Figure 7:**
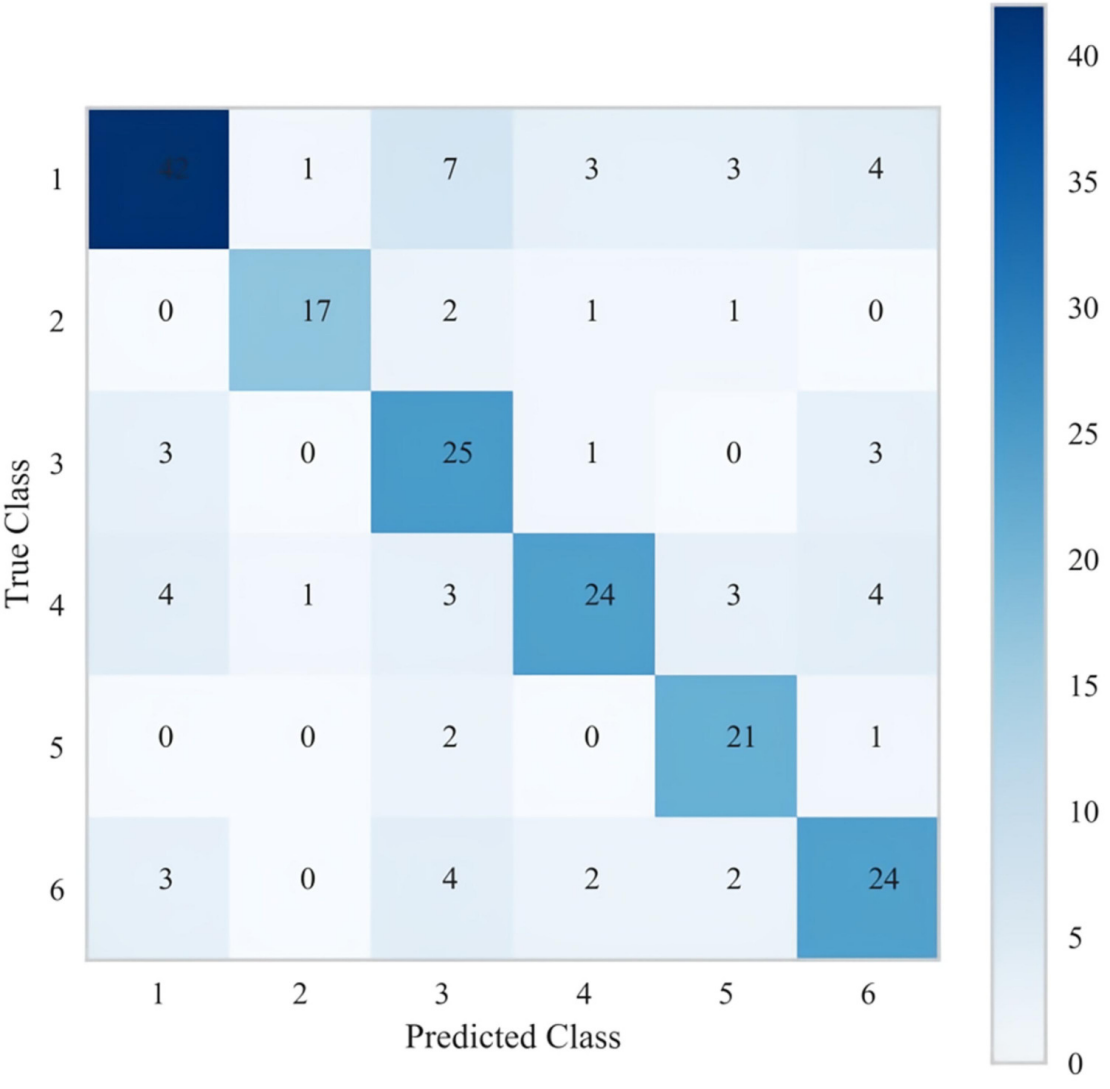
Heat map of the confusion matrix of the validation set for the LGBM model.

**Figure 8:**
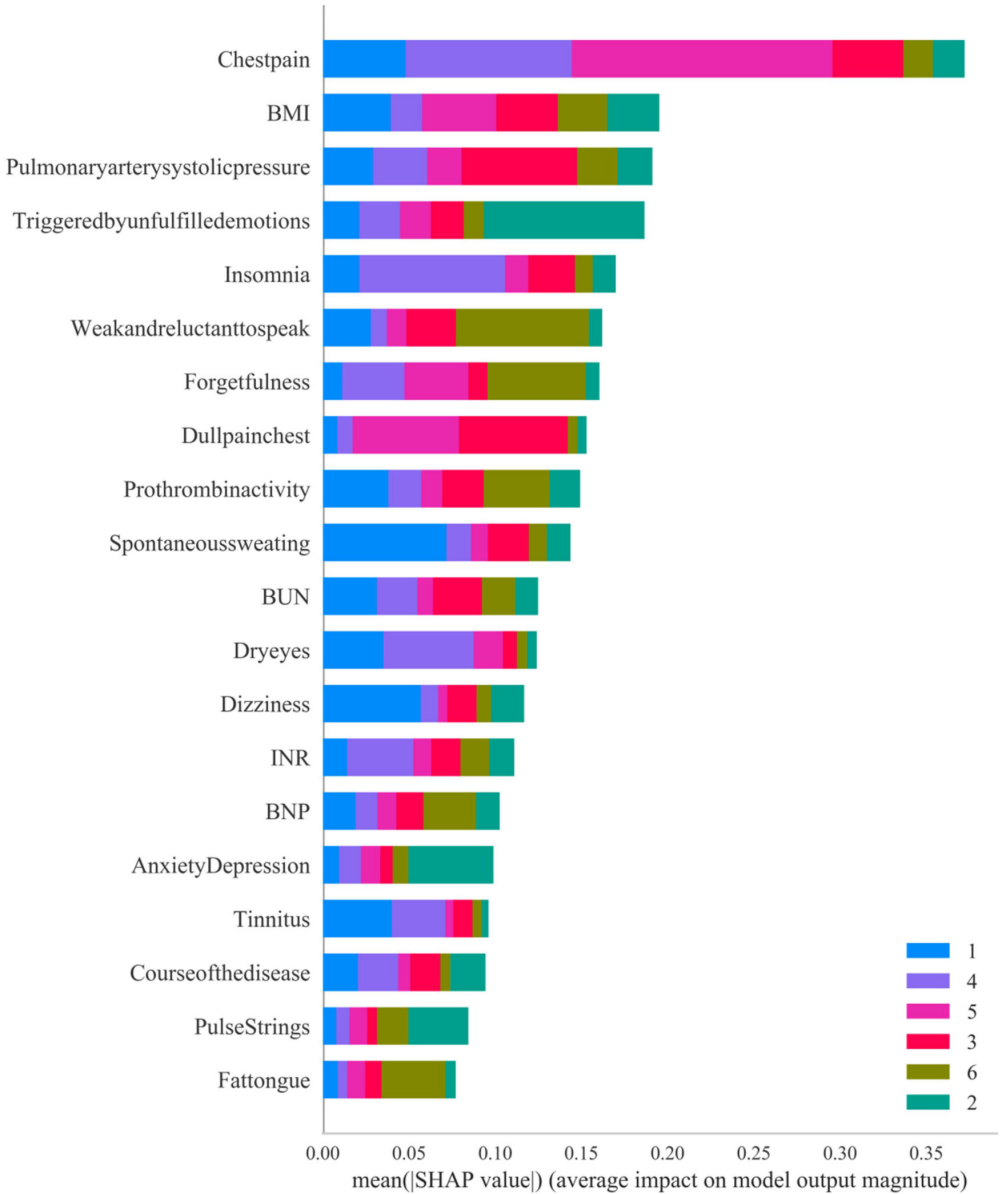
Summary of the contribution of SHAP values in the LGBM model.

Due to overfitting in the Logistic model, therefore the final results showed that LGBM machine learning performed the best (validation set = 72.5%). The specific results of the training set and validation set are shown in Supplementary Tables S4, S5 respectively. Figure 5 below shows the learning curves of the training set and validation set of the LGBM model. Figure 6 is the heat map of the confusion matrix of the training set of the LGBM model. Figure 7 is the heat map of the confusion matrix of the validation set of the LGBM model. Finally, the contribution of the feature factors included in the LGBM model is summarized using the SHAP model interpreter (Figure 8).

## 4 Discussion

This study reveals a significant correlation between TCM syndrome types and myocardial injury markers in patients with CHD, and uses TCM symptoms and myocardial injury markers to construct discriminative and predictive models for CHD syndrome types. Our research results are consistent with the development model of integrating myocardial injury markers with TCM syndrome differentiation, resolving the previous reliance on subjective clinical symptoms (11).

### 4.1 Distribution patterns of TCM syndrome types

The syndrome of phlegm blocking the heart pulse (295, 21%) and the syndrome of deficiency of both Qi and Yin (289, 20%) dominated in this study, which is in line with the contemporary epidemiological trend. Early multicenter studies (*N* = 7,512) determined that Qi deficiency and blood stasis was the most common pattern (12), while regional surveys in eastern China emphasized the high incidence of phlegm blocking the heart vessels, which might reflect the influence of diet and environment. The proportion of patients with phlegm-dampness obstruction syndrome is relatively high in South China, while in northern China, qi deficiency and blood stasis syndrome is predominant. Studies in different provinces have shown that Qi deficiency, phlegm turbidity, blood stasis and other syndrome types are distributed in all regions, but the specific proportions vary (13). Previous studies have indicated that the BMI values of patients with phlegm blocking the heart pulse syndrome are significantly different from those of other syndrome types, and the BMI value of patients with phlegm blocking the heart pulse syndrome is the highest (14), which is consistent with the results of this study.Other studies have shown that the average disease course of patients with phlegm blocking the heart and pulse syndrome (about 5–6 years) is significantly shorter than that of patients with Yang Qi deficiency, heart and kidney Yin deficiency, and Qi and Yin deficiency (6.5–7 years or more) (15), which is inconsistent with the results of this study. However, some research reports indicate that the average disease course of patients with phlegm blocking the heart and pulse syndrome is slightly longer than that of patients with heart blood stasis and Qi deficiency and blood stasis. It may reflect the specific pathological tendencies of this syndrome type in terms of metabolic abnormalities and arteriosclerosis (16).

When CHD is complicated with other diseases (such as hypertension, diabetes, and hyperlipidemia), the distribution of syndrome types will change. For instance, some studies have found that among patients with CHD complicated with hypertension, Qi deficiency and blood stasis syndrome and Qi stagnation and blood stasis syndrome are more common (17), while among patients with CHD complicated with diabetes, the proportion of Qi and Yin deficiency syndrome and phlegm and blood stasis intermingled syndrome is relatively high (18). However, there was no statistical difference in this study. Relevant studies have found that the distribution of syndrome types varies among patients of different age groups. For instance, young patients are mainly characterized by heart and liver syndrome, while middle-aged patients are mainly characterized by heart and kidney syndrome. Female patients have a higher proportion of Qi deficiency syndrome and phlegm-dampness syndrome, while male patients mainly have Qi deficiency and blood stasis syndrome (19). However, in this study, there was no difference in age or gender among patients with different syndrome types. With the passage of time, the distribution of TCM syndrome types of CHD shows certain changes. For instance, from the 1990s to the 2010s, the proportions of Qi deficiency and Yin deficiency gradually decreased, while the proportions of blood stasis and phlegm turbidity gradually increased (20).

In conclusion, the distribution pattern of TCM syndrome types for CHD mainly consists of Qi deficiency, blood stasis, phlegm turbidity, etc., and is influenced by factors such as BMI. These studies provide theoretical basis and practical guidance for the TCM syndrome differentiation and treatment of CHD.

### 4.2 Correlation between myocardial injury markers and TCM syndrome types

Myocardial injury markers such as cTnI, CK-MB, BNP, etc. have significant value in the diagnosis of myocardial injury. These markers can reflect the degree and type of myocardial damage and provide a basis for clinical treatment. For instance, cTnI is regarded as the “gold standard” for assessing myocardial injury, and an increase in its level is positively correlated with the severity of myocardial injury (21). Previous studies have shown that there is a certain correlation between different TCM syndrome types and myocardial injury markers. For instance, among patients with heart failure, the syndrome of Qi deficiency and blood stasis is significantly correlated with the level of NT-proBNP (22). This is consistent with the results of this study, confirming that as the patient's cardiac function declines and energy metabolism disorders occur, Qi deficiency further worsens, with weak propulsion, causing blood stasis and water retention, activation of the coagulation system, and excessive production of inflammatory factors, leading to disorders in the body's coagulation function and cytokines. TCM theory emphasizes syndrome differentiation and treatment. Research has found that certain TCM syndrome types may be related to specific mechanisms of myocardial injury. For instance, patients with Yang deficiency syndrome have a higher incidence of acute left heart failure (23), while those with Qi deficiency syndrome have a higher classification of cardiac function (24). These studies provide theoretical support for the application of TCM syndrome types in myocardial injury. In conclusion, certain progress has been made in the research on the correlation between myocardial injury markers and TCM syndrome types. However, it is still necessary to further improve research methods and standardize data to promote the application and development of integrated TCM and Western medicine in the field of myocardial injury.

#### 4.2.1 Correlation between BNP and TCM syndrome types

BNP is a neurohormone secreted by the heart and mainly participates in the regulation of the cardiovascular system. In patients with CHD, due to pathophysiological processes such as myocardial ischemia and ventricular remodeling, the secretion of BNP often increases. High levels of BNP not only reflect the decline of cardiac function but may also further aggravate the degree of myocardial ischemia by promoting mechanisms such as myocardial cell apoptosis and fibrosis (25). When myocardial cell damage occurs in CHD, neutrophils are activated, promoting inflammatory infiltration and granulocyte apoptosis, and the level of AnxA1 increases. When the BNP level rises in the later stage of cardiac load and enters the bloodstream, BNP is also one of the markers reflecting cardiac function (26).BNP was significantly positively correlated with New York Heart Association Functional Classification(NYHA) functional classification, and the BNP level increased with the increase of classification. The correlation coefficients were mostly between 0.3 and 0.8, all reaching statistical significance (*P* < 0.05) (27–30). They are important biomarkers for evaluating the cardiac function status of patients with coronary heart disease, guiding treatment decisions, and predicting prognosis.

This study found that among patients with CHD, there were significant differences in BNP levels among different TCM syndrome types. Among them, patients with Qi deficiency and blood stasis syndrome had the highest BNP levels, the left ventricular function of patients with this syndrome is more significantly impaired. The blood circulation block (blood stasis) leads to an increase in myocardial load, which in turn promotes an increase in ventricular wall tension and myocardial cell stress, and the secretion of BNP rises accordingly. In contrast, patients with Qi and Yin deficiency syndrome had the lowest BNP levels, it may reflect that the cardiac function is relatively stable, or that the body has formed a strong compensatory mechanism through the nourishment of Yin fluid, resulting in no significant increase in ventricular pressure and volume load. This provides an important basis for the precise treatment of integrated traditional Chinese and Western medicine. In the future, through large-scale sample verification and mechanism research, such biomarkers can be more systematically integrated into clinical syndrome differentiation and treatment decisions.

Relevant studies (31) have shown that among patients with heart failure, the average BNP value of Qi deficiency and blood stasis syndrome is 3,188.97 pg/mL, significantly higher than that of Qi and Yin deficiency syndrome (994.03 pg/mL). This data directly supports the conclusion that patients with qi deficiency and blood stasis syndrome have the highest BNP level. This study also found that BNP levels are positively correlated with the classification of cardiac function. The syndrome of Qi and Yin deficiency is mainly characterized by grade II cardiac function, while the syndrome of Qi deficiency and blood stasis is mainly characterized by grade III and IV cardiac function, further supporting the pathological mechanism of elevated BNP. Qi deficiency and blood stasis syndrome is the core pathogenesis of CHD. Its “deficiency at the root and excess at the symptoms” feature (Qi deficiency as the root and blood stasis as the symptoms) may lead to insufficient compensation of cardiac function, thereby causing an increase in BNP (32). In the early stage of heart failure, Qi deficiency syndrome is the main symptom. As the disease progresses, it may develop into Yang deficiency or Yin deficiency. The BNP level of Qi and Yin deficiency syndrome is relatively low, which may be related to the relatively stable heart function (33).

#### 4.2.2 Correlation between NT-proBNP and TCM syndrome types

NT-pro BNP is a cardiogenic neurohormone with the effect of dilating blood vessels. When the myocardium is damaged, the pressure of the ventricular wall increases, stimulating the secretion of NT-pro BNP. It is an important indicator for clinical assessment of cardiac function in patients with heart failure. Meanwhile, NT-proBNP can be used as a biomarker for evaluating the cardiac function classification of patients with coronary heart disease and monitoring the disease progression, helping doctors conduct more objective risk stratification based on the NYHA classification. Existing multiple clinical studies have confirmed that NT-proBNP is significantly positively correlated with the NYHA function classification of patients with coronary heart disease. Its correlation coefficient is approximately between 0.4 and 0.65, indicating reliable clinical application value (34).Therefore, The level of NT-proBNP in serum is closely related to the degree of myocardial ischemia and the severity of the disease in patients with CHD (35, 36).

This study found that among patients with CHD, there were significant differences in NT-proBNP levels among different TCM syndrome types. Among them, the NT-proBNP level was the highest in patients with Qi deficiency and blood stasis syndrome, the most obvious reflection is myocardial stress, with a relatively severe deterioration of cardiac function (such as grade III or IV cardiac function), and the degree of blood stasis may be even deeper (37), while the NT-proBNP level was the lowest in patients with phlegm blocking the heart vein syndrome. It may be related to the fact that this syndrome type is mainly characterized by “phlegm turbidity”, and the compensatory mechanism of cardiac function is relatively strong (38).This discovery provides quantifiable biological basis for syndrome differentiation and typing in TCM, which is conducive to achieving precise treatment through the integration of TCM and Western medicine.

The previous research results (38) have certain differences from the current results: The NT-proBNP among different syndrome types, from high to low, is Yang deficiency with water retention syndrome, phlegm turbidity blocking the lung syndrome, Qi and Yin deficiency syndrome, heart and lung Qi deficiency syndrome, and Qi deficiency with blood stasis syndrome, and the differences are statistically significant (*P* < 0.05). This may be related to the sample size, inclusion criteria and detection methods of different studies.

### 4.3 The correlation between TCM symptoms and TCM syndrome types

#### 4.3.1 Deficiency of both Qi and Yin syndrome

In the discriminant, spontaneous sweating (coefficient = 2.167) and dizziness (coefficient = 2.368) are most closely related to the syndrome of Qi and Yin deficiency, indicating that spontaneous sweating, dizziness and vertigo are closely associated with Qi and Yin deficiency. In the SHAP interpreter of the predictive model, symptoms such as dizziness, spontaneous sweating, and relief after rest are closely related to deficiency of both Qi and Yin.

Qi and Yin deficiency syndrome holds an important position in the TCM diagnosis of CHD. Its clinical manifestations include palpitations, chest tightness and dull pain, dizziness, spontaneous sweating or night sweats, hot palms, soles and chest, dry mouth, insomnia, etc (10). ① Spontaneous sweating and deficiency of both Qi and Yin syndrome. The key pathogenesis of spontaneous sweating lies in the imbalance of Yin and Yang and the loss of control over the opening and closing of skin, that is, Qi deficiency fails to retain sweat.

② Dizziness, vertigo and deficiency of both Qi and Yin syndrome. Due to Qi deficiency, one cannot nourish the head and eyes, while Yin deficiency causes false fire to be disturbed, leading to symptoms such as dizziness, tinnitus and vertigo.

In conclusion, symptoms such as spontaneous sweating and dizziness in patients with CHD are closely related to the syndrome of deficiency of both Qi and Yin. The syndrome of Qi and Yin deficiency is caused by the combined effect of Qi deficiency and Yin deficiency, leading to the failure of the heart meridian to be nourished and resulting in a series of symptoms. In terms of treatment, the focus should be on tonifying Qi and nourishing Yin, promoting blood circulation and unblocking meridians. Commonly used TCM include Codonopsis pilosula, Astragalus membranaceus, Ophiopogon japonicus and Salvia miltiorrhiza, etc. At the same time, patients should pay attention to dietary, lifestyle and psychological care to improve symptoms and enhance the quality of life.

#### 4.3.2 Qi stagnation and blood stasis syndrome

Among the discriminative criteria, distension and pain (chest) (coefficient >4), induction by emotional distress (coefficient >4), distension and fullness in the chest and hypochondrium (coefficient >3), irritability and anger (coefficient >2), scan-like coating on the tongue (coefficient >3), tauted pulse (coefficient = 2.831), and mirror tongue (coefficient >4) have the highest correlation coefficients with Qi stagnation and blood stasis syndrome. In the SHAP interpreter of the predictive model, taut pulse, emotional distress, anxiety and depression are closely related to the syndrome of Qi stagnation and blood stasis.

The chapter on Chest pain in “Internal Medicine of Traditional Chinese Medicine” mentions that the characteristics of chest pain in the syndrome of Qi stagnation and blood stasis are “stabbing pain, aggravated at night, accompanied by distension and oppression in the chest and hypochondrium, induced or aggravated by emotional fluctuations, dark purple tongue, and taut or sluggish pulse” (39). ① Distending pain and Qi stagnation and blood stasis: Distending pain in the chest and hypochondrium is an external manifestation of Qi stagnation and blood stasis. Qi stagnation leads to distension and fullness in the chest, hypochondrium, epigastrium and abdomen, while blood stasis is manifested as stabbing pain, dark purple tongue, taut and sluggish pulse, etc. In the scoring criteria of the “International Clinical Practice Guidelines for Traditional Chinese Medicine—Stable Angina Pectoris in Coronary Heart Disease”, hypochondriac distension or hypochondriac pain (3 points) is one of the key indicators for diagnosing Qi stagnation and blood stasis (40). ② Emotional distress, distension and fullness in the chest and hypochondrium, irritability and anger, and Qi stagnation: Emotional fluctuations are important triggers for Qi stagnation and blood stasis. Long-term negative emotions such as depression, anxiety, irritability and anger can damage liver Qi, leading to liver Qi stagnation and poor Qi circulation. The Huangdineijing states that “anger causes the Qi to rise”, which directly leads to the disorder of Qi circulation and aggravates blood stasis. ③ Scanty coating and Qi stagnation: In patients with CHD, Qi stagnation itself does not directly cause scanty coating. However, if Qi stagnation persists for a long time and depletes Qi and blood, or if it interacts with phlegm-dampness and blood stasis, it may indirectly affect the metabolism of body fluids, leading to scanty coating. ④ Taut pulse and Qi stagnation: Taut pulse is often related to the obstruction of Qi movement and the tension of the pulse channels. Modern medical research indicates that pathological taut pulses are associated with factors such as arteriosclerosis, increased blood viscosity, and increased peripheral resistance of the circulatory system. According to TCM, Qi stagnation and blood stasis can lead to poor circulation of Qi and blood, constricting and rapid pulse pathways, and thus manifest as taut pulse (41). ⑤ Mirror-like tongue and Qi stagnation: Mirror-like tongue (completely without coating) is more commonly seen in Qi deficiency or Yin deficiency syndrome types, rather than simply Qi stagnation and blood stasis. If patients with coronary heart disease suffer from poor circulation of Qi and blood due to Qi stagnation and blood stasis, it may further deplete Qi and blood, indirectly leading to insufficient body fluids and thus resulting in mirror-like tongue. ⑥ The unity of pathological mechanism and clinical manifestations: The formation mechanism of Qi stagnation and blood stasis syndrome is a vicious cycle of “Qi stagnation → blood stasis”. Qi stagnation leads to poor blood circulation, and blood stasis further aggravates the stagnation of Qi movement, resulting in a pathological state of mixed deficiency and excess. Qi stagnation and blood stasis is an excess syndrome, while long-term emotional stress may develop into “Qi deficiency and blood stasis” (deficiency syndrome). However, both are characterized by common features such as chest pain, chest tightness, dark purple tongue, and taut and sluggish pulse (42). ⑦ Correspondence between treatment and symptoms: Promoting blood circulation and removing blood stasis, as well as regulating Qi and relieving pain, are the core strategies for treating Qi stagnation and blood stasis syndrome. If the patient has “significant chest pain and obvious blood stasis”, drugs such as Corydalis yanhusuo and turmeric that promote blood circulation and remove blood stasis can be added. If there is “significant distension and fullness with obvious Qi stagnation”, then drugs such as agarwood that regulate Qi should be added. This indicates that the relief of symptoms needs to target the dual pathogenesis of Qi stagnation and blood stasis.

In conclusion, Qi stagnation and blood stasis syndrome is an important TCM syndrome type of CHD, and its core symptoms (stabbing pain, distending pain) are closely related to emotions such as irritability and anger. Emotional fluctuations cause Qi stagnation by damaging liver Qi, which in turn leads to blood stasis, creating a vicious cycle of “Qi stagnation → blood stasis → intensified pain”. The clinical manifestations include fixed chest pain, distension in the hypochondrium, dark purple tongue, taut and sluggish pulse, etc. The treatment should focus on promoting blood circulation and removing blood stasis, regulating Qi and relieving pain, while also paying attention to emotional management.

#### 4.3.3 Phlegm blocking the heart pulse syndrome

In the discriminant formula, dull pain and alcohol and overeating induction are most closely related to the syndrome of phlegm blocking the heart pulse (coefficient >5), while in the predictive model SHAP interpreter, dull pain, pulmonary artery systolic pressure are closely associated with the syndrome of phlegm blocking the heart pulse.

Multiple studies have clearly pointed out that the core symptoms of phlegm blocking the heart and pulse syndrome include chest tightness, excessive phlegm, obesity, white and greasy or slippery tongue coating, and slippery pulse, etc (34). For instance, it is mentioned that “phlegm blocking the heart meridian is mainly characterized by dull pain, and symptoms of excessive phlegm turbidity are also observed” (43); it is further emphasized that “severe chest tightness and mild heart pain” is a typical manifestation of phlegm turbidity and obstruction (44). These descriptions indicate that chest tightness and pain are highly correlated with the syndrome of phlegm blocking the heart pulse in terms of symptoms. This study also confirmed that patients with phlegm blocking the heart pulse syndrome of CHD have a higher BMI than those with other syndrome types. Drinking alcohol and overeating directly induce the syndrome of phlegm blocking the heart pulse by damaging the spleen, promoting the generation of phlegm and dampness, and causing lipid disorders, thereby aggravating the pathological process of CHD.

In clinical syndrome differentiation, the diagnosis of phlegm blocking the heart and pulse syndrome requires the combination of tongue appearance (white and greasy coating on the tongue), pulse appearance (slippery pulse), and physical signs (obesity, excessive phlegm). For instance, some studies have detailed the diagnostic criteria for the syndrome of phlegm blocking the heart pulse, including “chest tightness and shortness of breath, or distension and pain; pain in the back or left arm; shortness of breath and wheezing excessive phlegm; fat body; the coating on the tongue is turbid and greasy or slippery. the pulse is slippery or taut and slippery” (45). The treatment should focus on promoting Yang circulation, eliminating turbidity, clearing phlegm and resolving nodules. These characteristics further support the close connection between the syndrome of phlegm blocking the heart pulse and dull pain.

#### 4.3.4 Syndrome of Yin deficiency of heart and kidney

Among the discriminant, red tongue, tinnitus and insomnia are most closely related to the syndrome of heart and kidney Yin deficiency (coefficient >2), while in the predictive model SHAP interpreter, insomnia, dry eyes, forgetfulness and tinnitus are closely associated with the syndrome of heart and kidney Yin deficiency.

Relevant guidelines for CHD (10) indicate that patients with heart and kidney Yin deficiency syndrome present with symptoms such as soreness and weakness in the waist and knees, palpitations, insomnia, hot flushes in the palms, soles and chest, dry mouth and throat, night sweats, red tongue with little coating, and fine and rapid pulse. Red tongue (with a red tongue body) is a typical manifestation of Yin deficiency and hyperactivity of internal heat. Insufficiency of kidney Yin prevents essence and qi from ascending to the ear orifices, leading to tinnitus. Patients with heart and kidney Yin deficiency syndrome often have tinnitus, which worsens especially after fatigue or hunger, and is mostly high-pitched tinnitus accompanied by a feeling of ear blockage. When kidney Yin is insufficient and false fire rises to disturb the heart spirit, it leads to restlessness of the heart spirit, which is manifested as insomnia. When the heart and kidney are not in harmony, the heart fire becomes excessive, disturbing the mind and aggravating insomnia.

The therapeutic principle centers on nourishing Yin and benefiting the kidneys, as well as nourishing the heart and calming the mind. It is recommended to use prescriptions such as Liuwei Dihuang Pills, Tianwang Buxin Dan, and Qiju Dihuang Pills. Modern research has found that the method of nourishing kidney Yin can reduce the levels of serum ET(endothelin) and MDA(Malondialdehyde) in patients with CHD, increase SOD (Superoxide Dismutase) activity, and improve myocardial ischemia (46).

#### 4.3.5 Syndrome of blood stasis in the heart

Among the discriminants, chest pain (coefficient >5) and pain leading to the inner side of the shoulder and back (coefficient >3) are most associated with the syndrome of cardiovascular blood stasis, while in the predictive model SHAP interpreter, chest pain and others are closely related to the syndrome of cardiovascular blood stasis.

The typical symptoms of blood stasis syndrome are stabbing or colic pain in the chest and heart, with a fixed location, aggravated at night, and often radiating to the shoulder and back or the inner side of the left arm (10). The syndrome of blood stasis in the heart leads to the obstruction of the heart meridian, and the circulation of Qi and blood is not smooth. When there is no smooth flow, pain occurs. The Hand Shaoyin Heart Meridian “runs from the inner side of the shoulder to the lower chest, passes through the diaphragm, and spreads to the heart envelope”, following the inner side of the shoulder and back. Therefore, pain can be transmitted along the meridians to the shoulder and back. When the heart meridian is blocked, the stimulation of Qi and blood circulation will be transmitted along the meridians to these areas, presenting as radiating pain. Many studies have emphasized that chest pain radiating to the shoulder, back or left arm is a key point for differentiating the syndrome of blood stasis in the heart.

#### 4.3.6 Qi deficiency and blood stasis syndrome

Among the discriminants, a plump tongue (coefficient >2), fatigue and reluctance to speak (coefficient >4), and a sallow complexion (coefficient >5) are most closely related to the syndrome of Qi deficiency and blood stasis. However, in the predictive model SHAP interpreter, fatigue and reluctance to speak, forgetfulness, BNP and a plump tongue are closely associated with the syndrome of Qi deficiency and blood stasis.

The typical symptoms of Qi deficiency and blood stasis syndrome include chest pain, which is characterized by chest pain and tightness, and is triggered by physical exertion. The symptoms include shortness of breath, fatigue, listlessness, reluctance to speak, palpitations, spontaneous sweating, pale or dull complexion, plump and pale tongue, and deep and sluggish pulse (10). Qi deficiency fails to warm the spleen Yang, leading to internal retention of water and dampness and swelling of the tongue. At the same time, Qi deficiency leads to weak propulsion and blood circulation is blocked, resulting in blood stasis syndrome, with Qi deficiency as the root cause and blood stasis as the symptom. Deficiency of heart Qi fails to promote the circulation of Qi and blood, leading to blood stasis and obstruction, which is manifested as symptoms of deficiency such as fatigue and laziness in speech. Qi deficiency fails to warm and nourish the internal organs, leading to the internal generation of cold and dampness. Cold coagulation causes blood stasis, which is manifested as dull complexion and rough skin.Qi deficiency leads to insufficient blood circulation, and blood stasis further blocks the cerebral blood vessels, which is the main pathogenesis of forgetfulness in patients with CHD in TCM. The “Classification of Syndromes and Treatment of Forgetfulness” points out that blood stasis is an important factor causing forgetfulness. When Qi deficiency aggravates blood stasis, it is easy to cause memory disorders. Studies have shown that TCM intervention (such as Qihong Powder, Yiqi Fumai Injection, etc.) can significantly reduce BNP levels, and the extent of BNP reduction is consistent with the improvement degree of Qi deficiency and blood stasis syndrome, suggesting that BNP can be used for therapeutic effect monitoring (47). In conclusion, Qi deficiency and blood stasis syndrome is significantly positively correlated with serum BNP levels, and BNP can be used as an objective biomarker for this syndrome. Help assess cardiac function, stratify risks and monitor the therapeutic effects of TCM.

## 5 Limitations and prospects

As there is no unified standard for the TCM syndrome types of CHD at present, the clinical data collection this time found that the syndrome of phlegm blocking the heart pulse accounted for the largest proportion (20.5%), and the syndrome of phlegm turbidity and blood stasis occurred most frequently among the 17 guidelines/concoms (*N* = 16). Meanwhile, this study has the problem that the probability of some unordered multiple logistic regression is unstable due to the scarcity of categories, so it should be interpreted with caution. In addition, the lack of detailed clinical medication data may lead to the association between the myocardial injury markers observed in the study and the TCM syndrome types and symptoms being affected by unmeasured confounding factors. Therefore, future research should focus on establishing a unified classification standard for TCM syndrome types, and combine modern biomarker technology to deeply explore the causal relationship between myocardial injury markers and TCM syndrome types. At the same time, systematic medication information should be integrated to more accurately assess the independent association between biomarkers and TCM syndrome differentiation. Finally, research on the integration of TCM and Western medicine should be strengthened. To improve the diagnosis and treatment effect of myocardial injury (48).

## 6 Summary

This study constructed a prediction model for TCM syndrome types. The modeling plan was divided into principal analysis and exploratory analysis. Among them, the establishment of the prediction model was the principal analysis, while unordered multiple logistic regression and discriminant analysis were exploratory analyses. This study provides empirical evidence for the verification of myocardial injury markers in the TCM syndrome differentiation of CHD, builds a biochemical bridge between myocardial injury indicators and TCM pathophysiology as well as modern cardiology, and lays the foundation for the formulation of personalized comprehensive treatment strategies. Future research should further verify the role of the above-mentioned biomarkers in predicting treatment outcomes and deeply explore their application potential in guiding the combination of TCM.

## Data Availability

The raw data supporting the conclusions of this article will be made available by the authors, without undue reservation.
