# Supplementary material for: A study on the correlation between TCM syndrome types, TCM symptoms and myocardial injury markers in patients with coronary heart disease
Source: Front Cardiovasc Med. 2026 Jan 12;12:1669239. doi: 10.3389/fcvm.2025.1669239 (PMC12833428; doi:10.3389/fcvm.2025.1669239)
Supplement: Supplementary file 1 [file Datasheet1.docx]

**Table S1 Unordered multi-classification logistic regression analysis of patients with different syndrome types of coronary heart disease**

| TCM syndrome types^a^ | | B | Standard error | Wald | Significance | Exp(B) | Exp(B) 95% CI | |
| --- | --- | --- | --- | --- | --- | --- | --- | --- |
|  |  |  |  |  |  |  | Lower limit | Upper limit |
| Qi deficiency and blood stasis syndrome | Intercept | -232.379 | 1573.604 | .022 | .883 |  |  |  |
|  | [Palpitations =0] | 7.290 | 3.913 | 3.470 | .062 | 1465.236 | .684 | 3140554.277 |
|  | [Palpitations =1] | 0^b^ | . | . | . | . | . | . |
|  | [Chest tightness =0] | 5.591 | 4.094 | 1.865 | .172 | 267.925 | .088 | 817963.928 |
|  | [Chest tightness =1] | 0^b^ | . | . | . | . | . | . |
|  | [Shortness of breath =0] | 5.497 | 4.129 | 1.772 | .183 | 243.954 | .075 | 797837.736 |
|  | [Shortness of breath =1] | 0^b^ | . | . | . | . | . | . |
|  | [Chest pain =0] | 18.038 | 14.170 | 1.621 | .203 | 68191374.450 | 5.924E-5 | 78497600833496300000.000 |
|  | [Chest pain =1] | 0^b^ | . | . | . | . | . | . |
|  | [Precordial area =0] | -6.575 | 7.822 | .707 | .401 | .001 | 3.069E-10 | 6343.521 |
|  | [Precordial area =1] | 0^b^ | . | . | . | . | . | . |
|  | [Zhongfu =0] | -.058 | 9.670 | .000 | .995 | .944 | 5.549E-9 | 160603028.487 |
|  | [Zhongfu =1] | 0^b^ | . | . | . | . | . | . |
|  | [Chest flap =0] | -2.722 | 7.977 | .116 | .733 | .066 | 1.067E-8 | 405249.258 |
|  | [Chest flap =1] | 0^b^ | . | . | . | . | . | . |
|  | [Pain on the inner side of the shoulder and arm =0] | 33.122 | 8.639 | 14.698 | .000 | 242467639329760.720 | 10735378.607 | 5476337470157912000000.000 |
|  | [Pain on the inner side of the shoulder and arm =1] | 0^b^ | . | . | . | . | . | . |
|  | [Pain has no fixed location =0] | 10.368 | 14.956 | .481 | .488 | 31820.241 | 5.912E-9 | 171272888733345984.000 |
|  | [Pain has no fixed location =1] | 0^b^ | . | . | . | . | . | . |
|  | [The pain has a fixed location =0] | 15.385 | 11.057 | 1.936 | .164 | 4802164.051 | .002 | 12400796490127918.000 |
|  | [The pain has a fixed location =1] | 0^b^ | . | . | . | . | . | . |
|  | [Stabbing pain =0] | 10.278 | 7.995 | 1.652 | .199 | 29071.573 | .005 | 185719568924.424 |
|  | [Stabbing pain =1] | 0^b^ | . | . | . | . | . | . |
|  | [Dull pain =0] | -10.094 | 8.588 | 1.381 | .240 | 4.131E-5 | 2.021E-12 | 844.465 |
|  | [Dull pain =1] | 0^b^ | . | . | . | . | . | . |
|  | [Distending pain =0] | -27.131 | 22.426 | 1.464 | .226 | 1.649E-12 | 1.344E-31 | 20238374.215 |
|  | [Distending pain =1] | 0^b^ | . | . | . | . | . | . |
|  | [Colic =0] | 6.462 | 10.730 | .363 | .547 | 640.128 | 4.705E-7 | 870974749913.421 |
|  | [Colic =1] | 0^b^ | . | . | . | . | . | . |
|  | [Dull pain =0] | -28.593 | 13.100 | 4.764 | .029 | 3.821E-13 | 2.700E-24 | .054 |
|  | [Dull pain =1] | 0^b^ | . | . | . | . | . | . |
|  | [Post-activity increase =0] | -4.120 | 5.116 | .648 | .421 | .016 | 7.178E-7 | 367.982 |
|  | [Post-activity increase =1] | 0^b^ | . | . | . | . | . | . |
|  | [Induced by unfulfilled emotions =0] | -10.371 | 8.243 | 1.583 | .208 | 3.133E-5 | 3.015E-12 | 325.611 |
|  | [Induced by unfulfilled emotions =1] | 0^b^ | . | . | . | . | . | . |
|  | [Induced by drinking and overeating =0]] | -59.867 | 26.093 | 5.264 | .022 | 1.001E-26 | 6.161E-49 | .000 |
|  | [Induced by drinking and overeating =1] | 0^b^ | . | . | . | . | . | . |
|  | [Induced by rainy days =0] | 22.256 | 8.384 | 7.047 | .008 | 4632866615.869 | 338.501 | 63407445950178104.000 |
|  | [Induced by rainy days =1] | 0^b^ | . | . | . | . | . | . |
|  | [Sudden cold weather or sudden exposure to cold wind triggers =0] | -9.144 | 14.145 | .418 | .518 | .000 | 9.746E-17 | 117249930.566 |
|  | [Sudden cold weather or sudden exposure to cold wind triggers =1] | 0^b^ | . | . | . | . | . | . |
|  | [Relief after rest =0] | -3.637 | 1.995 | 3.325 | .068 | .026 | .001 | 1.313 |
|  | [Relief after rest =1] | 0^b^ | . | . | . | . | . | . |
|  | [Edema =0] | 3.245 | 7.681 | .178 | .673 | 25.663 | 7.434E-6 | 88583565.486 |
|  | [Edema =1] | 0^b^ | . | . | . | . | . | . |
|  | [Weak and talkative =0] | -12.587 | 3.833 | 10.781 | .001 | 3.416E-6 | 1.864E-9 | .006 |
|  | [Weak and talkative =1] | 0^b^ | . | . | . | . | . | . |
|  | [Feeling annoyed =0] | -7.904 | 5.837 | 1.834 | .176 | .000 | 3.976E-9 | 34.325 |
|  | [Feeling annoyed =1] | 0^b^ | . | . | . | . | . | . |
|  | [Forgetfulness =0] | -3.034 | 5.572 | .296 | .586 | .048 | 8.706E-7 | 2662.011 |
|  | [Forgetfulness =1] | 0^b^ | . | . | . | . | . | . |
|  | [Heat intolerance =0] | -4.315 | 4.894 | .777 | .378 | .013 | 9.136E-7 | 195.681 |
|  | [Heat intolerance =1] | 0^b^ | . | . | . | . | . | . |
|  | [Fear of cold =0] | -12.300 | 7.405 | 2.759 | .097 | 4.550E-6 | 2.263E-12 | 9.147 |
|  | [Fear of cold =1] | 0^b^ | . | . | . | . | . | . |
|  | [Cold limbs =0] | -6.010 | 6.303 | .909 | .340 | .002 | 1.059E-8 | 568.666 |
|  | [Cold limbs =1] | 0^b^ | . | . | . | . | . | . |
|  | [Hot and humid =0] | -1.457 | 9.940 | .021 | .883 | .233 | 8.059E-10 | 67322191.016 |
|  | [Hot and humid =1] | 0^b^ | . | . | . | . | . | . |
|  | [Hot palms and soles =0] | 2.852 | 14.935 | .036 | .849 | 17.317 | 3.354E-12 | 89419072455397.380 |
|  | [Hot palms and soles =1] | 0^b^ | . | . | . | . | . | . |
|  | [Spontaneous sweating =0] | -15.414 | 4.067 | 14.367 | .000 | 2.021E-7 | 6.983E-11 | .001 |
|  | [Spontaneous sweating =1] | 0^b^ | . | . | . | . | . | . |
|  | [Night sweats =0] | 2.387 | 6.661 | .128 | .720 | 10.881 | 2.326E-5 | 5089776.821 |
|  | [Night sweats =1] | 0^b^ | . | . | . | . | . | . |
|  | [Dizziness =0] | -6.894 | 4.560 | 2.285 | .131 | .001 | 1.330E-7 | 7.721 |
|  | [Dizziness =1] | 0^b^ | . | . | . | . | . | . |
|  | [Dry eyes =0] | -4.552 | 4.267 | 1.138 | .286 | .011 | 2.462E-6 | 45.218 |
|  | [Dry eyes =1] | 0^b^ | . | . | . | . | . | . |
|  | [Tinnitus =0] | 8.154 | 4.477 | 3.316 | .069 | 3476.865 | .537 | 22511495.498 |
|  | [Tinnitus =1] | 0^b^ | . | . | . | . | . | . |
|  | [Mouth ulcers =0] | 9.466 | 5.527 | 2.933 | .087 | 12912.044 | .255 | 654529766.994 |
|  | [Mouth ulcers =1] | 0^b^ | . | . | . | . | . | . |
|  | [Swollen and painful gums =0] | 16.226 | 13.105 | 1.533 | .216 | 11141285.500 | 7.792E-5 | 1593054543376661760.000 |
|  | [Swollen and painful gums =1] | 0^b^ | . | . | . | . | . | . |
|  | [Cough =0] | -9.995 | 5.633 | 3.149 | .076 | 4.561E-5 | 7.322E-10 | 2.841 |
|  | [Cough =1] | 0^b^ | . | . | . | . | . | . |
|  | [White and thin phlegm =0] | -1.745 | 8.088 | .047 | .829 | .175 | 2.278E-8 | 1338734.232 |
|  | [White and thin phlegm =1] | 0^b^ | . | . | . | . | . | . |
|  | [White thick phlegm =0] | 3.877 | 10.760 | .130 | .719 | 48.303 | 3.350E-8 | 69639950716.761 |
|  | [White thick phlegm =1] | 0^b^ | . | . | . | . | . | . |
|  | [Yellow sticky phlegm =0] | -14.142 | 16.575 | .728 | .394 | 7.214E-7 | 5.620E-21 | 92583216.178 |
|  | [Yellow sticky phlegm =1] | 0^b^ | . | . | . | . | . | . |
|  | Frothy sputum =0] | 64.872 | 17.989 | 13.005 | .000 | 14906719830276116000000000000.000 | 7264341212273.756 | 3.059E+43 |
|  | [Frothy sputum =1] | 0^b^ | . | . | . | . | . | . |
|  | [Stomach pain prefers warmth and pressure =0] | -9.769 | 12.333 | .627 | .428 | 5.722E-5 | 1.820E-15 | 1799459.124 |
|  | [Stomach pain prefers warmth and pressure =1] | 0^b^ | . | . | . | . | . | . |
|  | [Good Sigh =0] | 8.849 | 12.491 | .502 | .479 | 6968.856 | 1.626E-7 | 298611108963802.100 |
|  | [Good Sigh =1] | 0^b^ | . | . | . | . | . | . |
|  | [Costal distension =0] | -19.378 | 12.847 | 2.275 | .131 | 3.838E-9 | 4.454E-20 | 330.730 |
|  | [Costal distension =1] | 0^b^ | . | . | . | . | . | . |
|  | [Weakness in the waist and knees =0] | 2.378 | 4.064 | .342 | .558 | 10.782 | .004 | 31016.928 |
|  | [Weakness in the waist and knees =1] | 0^b^ | . | . | . | . | . | . |
|  | [Numbness in hands and feet =0] | 4.079 | 4.385 | .866 | .352 | 59.105 | .011 | 318989.777 |
|  | [Numbness in hands and feet =1] | 0^b^ | . | . | . | . | . | . |
|  | [Body aches all over =0] | -16.352 | 6.022 | 7.373 | .007 | 7.912E-8 | 5.916E-13 | .011 |
|  | [Body aches all over =1] | 0^b^ | . | . | . | . | . | . |
|  | [Thirsty and fond of drinking =0] | 4.426 | 4.706 | .885 | .347 | 83.614 | .008 | 847063.987 |
|  | [Thirsty and fond of drinking =1] | 0^b^ | . | . | . | . | . | . |
|  | [Preference for cold drinks =0] | -23.102 | 11.946 | 3.740 | .053 | 9.269E-11 | 6.294E-21 | 1.365 |
|  | [Preference for cold drinks =1] | 0^b^ | . | . | . | . | . | . |
|  | [Preference for hot drinks =0] | 1.183 | 4.659 | .064 | .800 | 3.264 | .000 | 30148.081 |
|  | [Preference for hot drinks =1] | 0^b^ | . | . | . | . | . | . |
|  | [Feeling thirsty but not wanting to drink or drinking very little =0] | .940 | 7.137 | .017 | .895 | 2.561 | 2.153E-6 | 3046170.376 |
|  | [Feeling thirsty but not wanting to drink or drinking very little =1] | 0^b^ | . | . | . | . | . | . |
|  | [Poor appetite =0] | -.659 | 6.023 | .012 | .913 | .518 | 3.868E-6 | 69257.515 |
|  | [Poor appetite =1] | 0^b^ | . | . | . | . | . | . |
|  | [Bitter taste in the mouth =0] | -4.644 | 5.559 | .698 | .404 | .010 | 1.782E-7 | 519.384 |
|  | [Bitter taste in the mouth =1] | 0^b^ | . | . | . | . | . | . |
|  | [Dry mouth =0] | 6.786 | 5.437 | 1.558 | .212 | 885.241 | .021 | 37577478.810 |
|  | [Dry mouth =1] | 0^b^ | . | . | . | . | . | . |
|  | [Sweet tooth =0] | -.103 | 5.129 | .000 | .984 | .902 | 3.885E-5 | 20947.399 |
|  | [Sweet tooth =1] | 0^b^ | . | . | . | . | . | . |
|  | [Preference for salty food =0] | 4.405 | 6.802 | .419 | .517 | 81.863 | .000 | 50482172.216 |
|  | [Preference for salty food =1] | 0^b^ | . | . | . | . | . | . |
|  | [Preference for strong tea =0] | -1.867 | 4.892 | .146 | .703 | .155 | 1.059E-5 | 2255.652 |
|  | [Preference for strong tea =1] | 0^b^ | . | . | . | . | . | . |
|  | [Love meat =0] | 1.937 | 6.389 | .092 | .762 | 6.937 | 2.530E-5 | 1901753.802 |
|  | [Love meat =1] | 0^b^ | . | . | . | . | . | . |
|  | [Long-term vegetarianism =0] | 7.662 | 10.334 | .550 | .458 | 2125.493 | 3.401E-6 | 1328515581359.559 |
|  | [Long-term vegetarianism =1] | 0^b^ | . | . | . | . | . | . |
|  | [Constipation =0] | -8.102 | 4.302 | 3.546 | .060 | .000 | 6.590E-8 | 1.392 |
|  | [Constipation =1] | 0^b^ | . | . | . | . | . | . |
|  | [Loose stools =0] | -3.815 | 11.762 | .105 | .746 | .022 | 2.144E-12 | 226679470.112 |
|  | [Loose stools =1] | 0^b^ | . | . | . | . | . | . |
|  | [Complete valley Unmelted =0] | -28.802 | 34.793 | .685 | .408 | 3.100E-13 | 7.504E-43 | 128047949285681744.000 |
|  | [Complete valley Unmelted =1] | 0^b^ | . | . | . | . | . | . |
|  | [Morning diarrhea =0] | 43.763 | 145.116 | .091 | .763 | 10138669702800474000.000 | 3.042E-105 | 3.379E+142 |
|  | [Morning diarrhea =1] | 0^b^ | . | . | . | . | . | . |
|  | [Loose and unregulated knots =0] | -28.829 | 26.697 | 1.166 | .280 | 3.019E-13 | 5.690E-36 | 16022707190.247 |
|  | [Loose and unregulated knots =1] | 0^b^ | . | . | . | . | . | . |
|  | [Clear and long urine =0] | 29.847 | 174.288 | .029 | .864 | 9172959773147.229 | 4.059E-136 | 2.073E+161 |
|  | [Clear and long urine =1] | 0^b^ | . | . | . | . | . | . |
|  | [Yellowish urine =0] | .487 | 15.858 | .001 | .975 | 1.628 | 5.173E-14 | 51252677111947.695 |
|  | [Yellowish urine =1] | 0^b^ | . | . | . | . | . | . |
|  | [Frequency of urination =0] | 8.602 | 7.351 | 1.369 | .242 | 5440.229 | .003 | 9834712648.736 |
|  | [Frequency of urination =1] | 0^b^ | . | . | . | . | . | . |
|  | [Burning urine =0] | 30.760 | 88.440 | .121 | .728 | 22847615527320.195 | 1.198E-62 | 4.356E+88 |
|  | [Burning urine =1] | 0^b^ | . | . | . | . | . | . |
|  | [Incomplete urination =0] | -1.559 | 8.472 | .034 | .854 | .210 | 1.293E-8 | 3422605.827 |
|  | [Incomplete urination =1] | 0^b^ | . | . | . | . | . | . |
|  | [Frequent nocturia =0] | -9.370 | 4.437 | 4.460 | .035 | 8.526E-5 | 1.426E-8 | .510 |
|  | [Frequent nocturia =1] | 0^b^ | . | . | . | . | . | . |
|  | [Insomnia=0] | -3.916 | 5.034 | .605 | .437 | .020 | 1.033E-6 | 384.345 |
|  | [Insomnia=1] | 0^b^ | . | . | . | . | . | . |
|  | [Excessive sleepiness =0] | 7.680 | 14.119 | .296 | .587 | 2163.912 | 2.074E-9 | 2257861245225359.000 |
|  | [Excessive sleepiness =1] | 0^b^ | . | . | . | . | . | . |
|  | [Irritable and quick-tempered =0] | 4.190 | 4.980 | .708 | .400 | 66.038 | .004 | 1144617.498 |
|  | [Irritable and quick-tempered =1] | 0^b^ | . | . | . | . | . | . |
|  | [Easy concern =0] | -10.111 | 11.330 | .796 | .372 | 4.061E-5 | 9.217E-15 | 178960.733 |
|  | [Easy concern =1] | 0^b^ | . | . | . | . | . | . |
|  | [Easily nervous =0] | 3.917 | 13.443 | .085 | .771 | 50.254 | 1.813E-10 | 13932786680537.781 |
|  | [Easily nervous =1] | 0^b^ | . | . | . | . | . | . |
|  | [Anxiety/Depression=0] | -4.374 | 8.119 | .290 | .590 | .013 | 1.547E-9 | 102641.564 |
|  | [Anxiety/Depression=1] | 0^b^ | . | . | . | . | . | . |
|  | [Prone to catching a cold =0] | -5.232 | 7.143 | .537 | .464 | .005 | 4.445E-9 | 6421.746 |
|  | [Prone to catching a cold =1] | 0^b^ | . | . | . | . | . | . |
|  | [Pale complexion =0] | -3.441 | 396.814 | .000 | .993 | .032 | .000 | .^c^ |
|  | [Pale complexion =1] | 0^b^ | . | . | . | . | . | . |
|  | [Red complexion (cheeks) =0] | 1.355 | 13.872 | .010 | .922 | 3.876 | 6.031E-12 | 2490891839549.771 |
|  | [Red complexion (cheeks) =1] | 0^b^ | . | . | . | . | . | . |
|  | [Pale red complexion =0] | 15.974 | 13.215 | 1.461 | .227 | 8657036.668 | 4.886E-5 | 1533820813958856960.000 |
|  | [Pale red complexion =1] | 0^b^ | . | . | . | . | . | . |
|  | [Dark red complexion =0] | -1.654 | 5.937 | .078 | .781 | .191 | 1.690E-6 | 21642.000 |
|  | [Dark red complexion =1] | 0^b^ | . | . | . | . | . | . |
|  | [Redness on both cheeks =0] | 6.488 | 5.747 | 1.275 | .259 | 657.288 | .008 | 51244343.180 |
|  | [Redness on both cheeks =1] | 0^b^ | . | . | . | . | . | . |
|  | [Pale yellow complexion =0] | .819 | 4.590 | .032 | .858 | 2.267 | .000 | 18320.700 |
|  | [Pale yellow complexion =1] | 0^b^ | . | . | . | . | . | . |
|  | [Yellow complexion =0] | 6.612 | 4.307 | 2.357 | .125 | 744.238 | .161 | 3450024.553 |
|  | [Yellow complexion =1] | 0^b^ | . | . | . | . | . | . |
|  | [Sallow complexion =0] | .950 | 6.377 | .022 | .882 | 2.585 | 9.649E-6 | 692487.150 |
|  | [Sallow complexion =1] | 0^b^ | . | . | . | . | . | . |
|  | [Sallow complexion =0] | 20.066 | 8.126 | 6.098 | .014 | 518357161.805 | 62.762 | 4281147927192004.000 |
|  | [Sallow complexion =1] | 0^b^ | . | . | . | . | . | . |
|  | [Pale complexion =0] | 12.839 | 7.218 | 3.164 | .075 | 376542.460 | .271 | 524038542010.765 |
|  | [Pale complexion =1] | 0^b^ | . | . | . | . | . | . |
|  | [Dark complexion =0] | 19.556 | 10.334 | 3.581 | .058 | 311367634.920 | .498 | 194789795921009056.000 |
|  | [Dark complexion =1] | 0^b^ | . | . | . | . | . | . |
|  | [Sallow complexion =0] | 31.134 | 35.694 | .761 | .383 | 33229483403548.293 | 1.376E-17 | 8.023E+43 |
|  | [Sallow complexion =1] | 0^b^ | . | . | . | . | . | . |
|  | [Radiant complexion =0] | -14.406 | 6.592 | 4.775 | .029 | 5.539E-7 | 1.355E-12 | .226 |
|  | [Radiant complexion =1] | 0^b^ | . | . | . | . | . | . |
|  | [Pale complexion =0] | -15.986 | 6.106 | 6.855 | .009 | 1.141E-7 | 7.248E-13 | .018 |
|  | [Pale complexion =1] | 0^b^ | . | . | . | . | . | . |
|  | [Pale complexion =0] | 6.562 | 1437.256 | .000 | .996 | 707.863 | .000 | .^c^ |
|  | [Pale complexion =1] | 0^b^ | . | . | . | . | . | . |
|  | [Dark eye orbit color =0] | -7.638 | 5.075 | 2.265 | .132 | .000 | 2.308E-8 | 10.062 |
|  | [Dark eye orbit color =1] | 0^b^ | . | . | . | . | . | . |
|  | [Pale lip nail =0] | -9.934 | 14.165 | .492 | .483 | 4.850E-5 | 4.251E-17 | 55346015.149 |
|  | [Pale lip nail =1] | 0^b^ | . | . | . | . | . | . |
|  | [Lip nail red =0] | -6.622 | 8.740 | .574 | .449 | .001 | 4.835E-11 | 36617.020 |
|  | [Lip nail red =1] | 0^b^ | . | . | . | . | . | . |
|  | [Light red lip nail =0] | -9.579 | 6.657 | 2.070 | .150 | 6.916E-5 | 1.490E-10 | 32.099 |
|  | [Light red lip nail =1] | 0^b^ | . | . | . | . | . | . |
|  | [Dark red lip nail =0] | -5.473 | 5.293 | 1.069 | .301 | .004 | 1.311E-7 | 134.444 |
|  | [Dark red lip nail =1] | 0^b^ | . | . | . | . | . | . |
|  | [Dark purple lip nail =0] | -9.905 | 7.159 | 1.914 | .167 | 4.993E-5 | 4.023E-11 | 61.974 |
|  | [Dark purple lip nail =1] | 0^b^ | . | . | . | . | . | . |
|  | [Lip and nail moisture =0] | 31.155 | 37.800 | .679 | .410 | 33927696448422.645 | 2.266E-19 | 5.080E+45 |
|  | [Lip and nail moisture =1] | 0^b^ | . | . | . | . | . | . |
|  | [Dry lips and nails =0] | 6.224 | 3.191 | 3.805 | .051 | 504.742 | .971 | 262485.508 |
|  | [Dry lips and nails =1] | 0^b^ | . | . | . | . | . | . |
|  | [Pale red tongue =0] | -7.046 | 9.940 | .503 | .478 | .001 | 3.013E-12 | 251621.115 |
|  | [Pale red tongue =1] | 0^b^ | . | . | . | . | . | . |
|  | [Pale tongue =0] | -6.924 | 28.291 | .060 | .807 | .001 | 8.147E-28 | 1187052491700182700000.000 |
|  | [Pale tongue =1] | 0^b^ | . | . | . | . | . | . |
|  | [Red tongue =0] | -19.997 | 9.787 | 4.175 | .041 | 2.067E-9 | 9.652E-18 | .443 |
|  | [Red tongue =1] | 0^b^ | . | . | . | . | . | . |
|  | [Purple Dark Tongue=0] | -16.861 | 9.513 | 3.142 | .076 | 4.756E-8 | 3.801E-16 | 5.952 |
|  | [Purple Dark Tongue=1] | 0^b^ | . | . | . | . | . | . |
|  | [Crimson tongue =0] | -19.223 | 11.435 | 2.826 | .093 | 4.485E-9 | 8.277E-19 | 24.300 |
|  | [Crimson tongue =1] | 0^b^ | . | . | . | . | . | . |
|  | [Red tongue tip =0] | 11.116 | 7.159 | 2.411 | .120 | 67221.271 | .054 | 83410287294.172 |
|  | [Red tongue tip =1] | 0^b^ | . | . | . | . | . | . |
|  | [Fat tongue =0] | -32.102 | 9.825 | 10.675 | .001 | 1.144E-14 | 4.959E-23 | 2.640E-6 |
|  | [Fat tongue =1] | 0^b^ | . | . | . | . | . | . |
|  | [Tooth mark tongue =0] | 1.885 | 2.781 | .459 | .498 | 6.583 | .028 | 1534.801 |
|  | [Tooth mark tongue =1] | 0^b^ | . | . | . | . | . | . |
|  | [Old tongue =0] | 3.310 | 2.820 | 1.378 | .240 | 27.397 | .109 | 6887.188 |
|  | [Old tongue =1] | 0^b^ | . | . | . | . | . | . |
|  | [Tender tongue =0] | -1.578 | 6.045 | .068 | .794 | .206 | 1.478E-6 | 28853.419 |
|  | [Tender tongue =1] | 0^b^ | . | . | . | . | . | . |
|  | [Cracked tongue =0] | 4.386 | 3.067 | 2.045 | .153 | 80.291 | .197 | 32730.550 |
|  | [Cracked tongue =1] | 0^b^ | . | . | . | . | . | . |
|  | [Ecchymosis tongue =0] | 4.865 | 5.081 | .917 | .338 | 129.621 | .006 | 2740601.043 |
|  | [Ecchymosis tongue =1] | 0^b^ | . | . | . | . | . | . |
|  | [Point the tongue =0] | -4.631 | 4.827 | .920 | .337 | .010 | 7.584E-7 | 125.318 |
|  | [Point the tongue =1] | 0^b^ | . | . | . | . | . | . |
|  | [Thin moss =0] | 6.920 | 8.883 | .607 | .436 | 1012.317 | 2.779E-5 | 36879461356.632 |
|  | [Thin moss =1] | 0^b^ | . | . | . | . | . | . |
|  | [Moss thickness =0] | 3.036 | 8.295 | .134 | .714 | 20.819 | 1.810E-6 | 239530565.283 |
|  | [Moss thickness =1] | 0^b^ | . | . | . | . | . | . |
|  | [Tai Ni =0] | -3.396 | 6.195 | .300 | .584 | .034 | 1.786E-7 | 6291.747 |
|  | [Tai Ni =1] | 0^b^ | . | . | . | . | . | . |
|  | [Moss decay =0] | 15.508 | 14.902 | 1.083 | .298 | 5433396.337 | 1.124E-6 | 26259513312857100000.000 |
|  | [Moss decay =1] | 0^b^ | . | . | . | . | . | . |
|  | [Tairun =0] | 6.368 | 9.581 | .442 | .506 | 582.971 | 4.076E-6 | 83372145580.078 |
|  | [Tairun =1] | 0^b^ | . | . | . | . | . | . |
|  | [Taizao =0] | -2.517 | 9.308 | .073 | .787 | .081 | 9.633E-10 | 6765125.213 |
|  | [Taizao =1] | 0^b^ | . | . | . | . | . | . |
|  | [Slippery tongue =0] | 26.056 | 46.535 | .314 | .576 | 206971192682.089 | 5.076E-29 | 8.438E+50 |
|  | [Slippery tongue =1] | 0^b^ | . | . | . | . | . | . |
|  | [Less moss =0] | -12.819 | 8.280 | 2.397 | .122 | 2.710E-6 | 2.425E-13 | 30.284 |
|  | [Less moss =1] | 0^b^ | . | . | . | . | . | . |
|  | [Mirror tongue =0] | 62.076 | 29.223 | 4.512 | .034 | 910524501291099800000000000.000 | 121.559 | 6.820E+51 |
|  | [Mirror tongue =1] | 0^b^ | . | . | . | . | . | . |
|  | [Full tongue coating =0] | -4.389 | 3.505 | 1.568 | .210 | .012 | 1.288E-5 | 11.950 |
|  | [Full tongue coating =1] | 0^b^ | . | . | . | . | . | . |
|  | [Moss peeling =0] | -18.781 | 4.154 | 20.438 | .000 | 6.974E-9 | 2.029E-12 | 2.397E-5 |
|  | [Moss peeling =1] | 0^b^ | . | . | . | . | . | . |
|  | [White coating on the tongue =0] | 30.366 | 30.919 | .965 | .326 | 15405208823214.102 | 7.404E-14 | 3.205E+39 |
|  | [White coating on the tongue =1] | 0^b^ | . | . | . | . | . | . |
|  | [Yellow coating on the tongue =0] | 29.529 | 31.054 | .904 | .342 | 6669474624856.311 | 2.458E-14 | 1.809E+39 |
|  | [Yellow coating on the tongue =1] | 0^b^ | . | . | . | . | . | . |
|  | [Yellow and white coating on the tongue =0] | 33.270 | 31.251 | 1.133 | .287 | 281284503530893.030 | 7.054E-13 | 1.122E+41 |
|  | [Yellow and white coating on the tongue =1] | 0^b^ | . | . | . | . | . | . |
|  | [Grayish-black coating on the tongue =0] | 36.364 | 23.108 | 2.476 | .116 | 6205086305256369.000 | .000 | 289850927581012270000000000000000000.000 |
|  | [Grayish-black coating on the tongue =1] | 0^b^ | . | . | . | . | . | . |
|  | [pulse float =0] | -6.376 | 5.166 | 1.523 | .217 | .002 | 6.811E-8 | 42.497 |
|  | [pulse float =1] | 0^b^ | . | . | . | . | . | . |
|  | [pulse depth =0] | -4.501 | 4.188 | 1.155 | .282 | .011 | 3.024E-6 | 40.712 |
|  | [pulse depth =1] | 0^b^ | . | . | . | . | . | . |
|  | [Pulse slow =0] | -5.147 | 10.519 | .239 | .625 | .006 | 6.467E-12 | 5229946.797 |
|  | [Pulse slow =1] | 0^b^ | . | . | . | . | . | . |
|  | [Pulse fat =0] | -3.017 | 3.762 | .643 | .423 | .049 | 3.076E-5 | 77.944 |
|  | [Pulse fat =1] | 0^b^ | . | . | . | . | . | . |
|  | [ Pulse flood =0] | 58.037 | 511.505 | .013 | .910 | 16043172906212792000000000.000 | .000 | .^c^ |
|  | [Pulse flood =1] | 0^b^ | . | . | . | . | . | . |
|  | [ pulse fineness =0] | -5.706 | 5.347 | 1.139 | .286 | .003 | 9.345E-8 | 118.469 |
|  | [ pulse fineness =1] | 0^b^ | . | . | . | . | . | . |
|  | [Weak pulse=0] | -7.114 | 9.607 | .548 | .459 | .001 | 5.407E-12 | 122363.380 |
|  | [Weak pulse=1] | 0^b^ | . | . | . | . | . | . |
|  | [pulse slippery =0] | 3.811 | 3.865 | .972 | .324 | 45.214 | .023 | 88126.449 |
|  | [pulse slippery =1] | 0^b^ | . | . | . | . | . | . |
|  | [pulse astringency =0] | 11.808 | 8.800 | 1.800 | .180 | 134342.917 | .004 | 4160607025050.794 |
|  | [pulse astringency =1] | 0^b^ | . | . | . | . | . | . |
|  | [pulse string =0] | -3.190 | 3.967 | .647 | .421 | .041 | 1.730E-5 | 98.003 |
|  | [pulse string =1] | 0^b^ | . | . | . | . | . | . |
|  | [pulse tightness =0] | 6.611 | 22.276 | .088 | .767 | 743.037 | 8.114E-17 | 6804383279492400000000.000 |
|  | [pulse tightness =1] | 0^b^ | . | . | . | . | . | . |
|  | [pulse knot representation =0] | 4.494 | 6.352 | .501 | .479 | 89.523 | .000 | 22860575.058 |
|  | [pulse knot representation =1] | 0^b^ | . | . | . | . | . | . |
| Qi and Yin deficiency syndrome | Intercept | -72.666 | 1475.876 | .002 | .961 |  |  |  |
|  | [Palpitations =0] | 7.354 | 3.867 | 3.617 | .057 | 1561.863 | .799 | 3054619.939 |
|  | [Palpitations =1] | 0^b^ | . | . | . | . | . | . |
|  | [Chest tightness =0] | 5.514 | 4.128 | 1.784 | .182 | 248.222 | .076 | 810189.541 |
|  | [Chest tightness =1] | 0^b^ | . | . | . | . | . | . |
|  | [Shortness of breath =0] | 3.905 | 4.089 | .912 | .340 | 49.660 | .016 | 150255.436 |
|  | [Shortness of breath =1] | 0^b^ | . | . | . | . | . | . |
|  | [Chest pain =0] | 35.719 | 13.793 | 6.706 | .010 | 3254447528653572.000 | 5916.978 | 1790006342160774500000000000.000 |
|  | [Chest pain =1] | 0^b^ | . | . | . | . | . | . |
|  | [Precordial area =0] | -11.284 | 7.731 | 2.130 | .144 | 1.258E-5 | 3.304E-12 | 47.890 |
|  | [Precordial area =1] | 0^b^ | . | . | . | . | . | . |
|  | [Zhongfu =0] | -3.503 | 9.537 | .135 | .713 | .030 | 2.296E-10 | 3950563.948 |
|  | [Zhongfu =1] | 0^b^ | . | . | . | . | . | . |
|  | [Chest flap =0] | -9.818 | 7.875 | 1.554 | .212 | 5.447E-5 | 1.079E-11 | 274.904 |
|  | [Chest flap =1] | 0^b^ | . | . | . | . | . | . |
|  | [Pain on the inner side of the shoulder and arm =0] | 34.953 | 8.731 | 16.025 | .000 | 1513566701530324.800 | 55942660.342 | 40950575928555760000000.000 |
|  | [Pain on the inner side of the shoulder and arm =1] | 0^b^ | . | . | . | . | . | . |
|  | [Pain has no fixed location =0] | 1.873 | 15.164 | .015 | .902 | 6.510 | 8.055E-13 | 52610966962474.020 |
|  | [Pain has no fixed location =1] | 0^b^ | . | . | . | . | . | . |
|  | [The pain has a fixed location =0] | 11.142 | 10.994 | 1.027 | .311 | 68990.365 | 3.027E-5 | 157239126155299.300 |
|  | [The pain has a fixed location =1] | 0^b^ | . | . | . | . | . | . |
|  | [Stabbing pain =0] | 13.982 | 8.099 | 2.980 | .084 | 1181250.684 | .151 | 9260513076890.361 |
|  | [Stabbing pain =1] | 0^b^ | . | . | . | . | . | . |
|  | [Dull pain =0] | -10.616 | 8.650 | 1.506 | .220 | 2.452E-5 | 1.063E-12 | 565.449 |
|  | [Dull pain =1] | 0^b^ | . | . | . | . | . | . |
|  | [Distending pain =0] | -29.155 | 22.458 | 1.685 | .194 | 2.178E-13 | 1.665E-32 | 2847762.417 |
|  | [Distending pain =1] | 0^b^ | . | . | . | . | . | . |
|  | [Colic =0] | 9.018 | 11.397 | .626 | .429 | 8252.059 | 1.643E-6 | 41446710081443.400 |
|  | [Colic =1] | 0^b^ | . | . | . | . | . | . |
|  | [Dull pain =0] | -25.996 | 13.103 | 3.936 | .047 | 5.131E-12 | 3.603E-23 | .731 |
|  | [Dull pain =1] | 0^b^ | . | . | . | . | . | . |
|  | [Post-activity increase =0] | -5.827 | 5.083 | 1.314 | .252 | .003 | 1.388E-7 | 62.573 |
|  | [Post-activity increase =1] | 0^b^ | . | . | . | . | . | . |
|  | [Induced by unfulfilled emotions =0] | -6.881 | 8.228 | .699 | .403 | .001 | 1.018E-10 | 10350.184 |
|  | [Induced by unfulfilled emotions =1] | 0^b^ | . | . | . | . | . | . |
|  | [Induced by drinking and overeating =0]] | -64.935 | 26.097 | 6.191 | .013 | 6.298E-29 | 3.852E-51 | 1.030E-6 |
|  | [Induced by drinking and overeating =1] | 0^b^ | . | . | . | . | . | . |
|  | [Induced by rainy days =0] | 19.169 | 8.354 | 5.265 | .022 | 211346488.979 | 16.358 | 2730535532045320.500 |
|  | [Induced by rainy days =1] | 0^b^ | . | . | . | . | . | . |
|  | [Sudden cold weather or sudden exposure to cold wind triggers =0] | -5.476 | 14.080 | .151 | .697 | .004 | 4.333E-15 | 4048013218.372 |
|  | [Sudden cold weather or sudden exposure to cold wind triggers =1] | 0^b^ | . | . | . | . | . | . |
|  | [Relief after rest =0] | -5.910 | 1.949 | 9.193 | .002 | .003 | 5.948E-5 | .124 |
|  | [Relief after rest =1] | 0^b^ | . | . | . | . | . | . |
|  | [Edema =0] | 4.068 | 7.723 | .277 | .598 | 58.419 | 1.560E-5 | 218799002.033 |
|  | [Edema =1] | 0^b^ | . | . | . | . | . | . |
|  | [Weak and talkative =0] | -4.205 | 3.764 | 1.248 | .264 | .015 | 9.325E-6 | 23.873 |
|  | [Weak and talkative =1] | 0^b^ | . | . | . | . | . | . |
|  | [Feeling annoyed =0] | -7.891 | 5.844 | 1.823 | .177 | .000 | 3.967E-9 | 35.274 |
|  | [Feeling annoyed =1] | 0^b^ | . | . | . | . | . | . |
|  | [Forgetfulness =0] | -10.393 | 5.554 | 3.501 | .061 | 3.065E-5 | 5.734E-10 | 1.638 |
|  | [Forgetfulness =1] | 0^b^ | . | . | . | . | . | . |
|  | [Heat intolerance =0] | -.735 | 4.901 | .022 | .881 | .479 | 3.228E-5 | 7123.313 |
|  | [Heat intolerance =1] | 0^b^ | . | . | . | . | . | . |
|  | [Fear of cold =0] | -8.036 | 7.419 | 1.173 | .279 | .000 | 1.566E-10 | 668.562 |
|  | [Fear of cold =1] | 0^b^ | . | . | . | . | . | . |
|  | [Cold limbs =0] | -5.586 | 6.356 | .772 | .379 | .004 | 1.458E-8 | 964.076 |
|  | [Cold limbs =1] | 0^b^ | . | . | . | . | . | . |
|  | [Hot and humid =0] | -5.351 | 9.761 | .301 | .584 | .005 | 2.331E-11 | 965199.891 |
|  | [Hot and humid =1] | 0^b^ | . | . | . | . | . | . |
|  | [Hot palms and soles =0] | -17.741 | 11.436 | 2.407 | .121 | 1.973E-8 | 3.641E-18 | 106.966 |
|  | [Hot palms and soles =1] | 0^b^ | . | . | . | . | . | . |
|  | [Spontaneous sweating =0] | -15.346 | 4.050 | 14.356 | .000 | 2.165E-7 | 7.726E-11 | .001 |
|  | [Spontaneous sweating =1] | 0^b^ | . | . | . | . | . | . |
|  | [Night sweats =0] | -6.587 | 6.638 | .985 | .321 | .001 | 3.082E-9 | 615.811 |
|  | [Night sweats =1] | 0^b^ | . | . | . | . | . | . |
|  | [Dizziness =0] | -12.155 | 4.606 | 6.964 | .008 | 5.262E-6 | 6.316E-10 | .044 |
|  | [Dizziness =1] | 0^b^ | . | . | . | . | . | . |
|  | [Dry eyes =0] | -4.417 | 4.212 | 1.100 | .294 | .012 | 3.138E-6 | 46.403 |
|  | [Dry eyes =1] | 0^b^ | . | . | . | . | . | . |
|  | [Tinnitus =0] | 11.545 | 4.527 | 6.503 | .011 | 103269.537 | 14.468 | 737133974.230 |
|  | [Tinnitus =1] | 0^b^ | . | . | . | . | . | . |
|  | [Mouth ulcers =0] | 2.695 | 5.525 | .238 | .626 | 14.805 | .000 | 746923.847 |
|  | [Mouth ulcers =1] | 0^b^ | . | . | . | . | . | . |
|  | [Swollen and painful gums =0] | .850 | 11.193 | .006 | .939 | 2.341 | 6.946E-10 | 7887017330.628 |
|  | [Swollen and painful gums =1] | 0^b^ | . | . | . | . | . | . |
|  | [Cough =0] | -8.577 | 5.643 | 2.310 | .129 | .000 | 2.964E-9 | 11.985 |
|  | [Cough =1] | 0^b^ | . | . | . | . | . | . |
|  | [White and thin phlegm =0] | -4.132 | 7.908 | .273 | .601 | .016 | 2.978E-9 | 86430.765 |
|  | [White and thin phlegm =1] | 0^b^ | . | . | . | . | . | . |
|  | [White thick phlegm =0] | 5.518 | 10.763 | .263 | .608 | 249.056 | 1.716E-7 | 361506759304.328 |
|  | [White thick phlegm =1] | 0^b^ | . | . | . | . | . | . |
|  | [Yellow sticky phlegm =0] | -18.062 | 16.558 | 1.190 | .275 | 1.431E-8 | 1.151E-22 | 1779179.117 |
|  | [Yellow sticky phlegm =1] | 0^b^ | . | . | . | . | . | . |
|  | Frothy sputum =0] | 43.581 | 17.748 | 6.029 | .014 | 8449063806448689200.000 | 6600.422 | 10815472163603683000000000000000000.000 |
|  | [Frothy sputum =1] | 0^b^ | . | . | . | . | . | . |
|  | [Stomach pain prefers warmth and pressure =0] | -10.036 | 12.335 | .662 | .416 | 4.379E-5 | 1.385E-15 | 1384771.766 |
|  | [Stomach pain prefers warmth and pressure =1] | 0^b^ | . | . | . | . | . | . |
|  | [Good Sigh =0] | 1.307 | 12.398 | .011 | .916 | 3.694 | 1.033E-10 | 132091661520.967 |
|  | [Good Sigh =1] | 0^b^ | . | . | . | . | . | . |
|  | [Costal distension =0] | -15.770 | 12.930 | 1.487 | .223 | 1.417E-7 | 1.396E-18 | 14371.633 |
|  | [Costal distension =1] | 0^b^ | . | . | . | . | . | . |
|  | [Weakness in the waist and knees =0] | 4.129 | 4.065 | 1.032 | .310 | 62.120 | .022 | 179103.929 |
|  | [Weakness in the waist and knees =1] | 0^b^ | . | . | . | . | . | . |
|  | [Numbness in hands and feet =0] | .914 | 4.394 | .043 | .835 | 2.495 | .000 | 13724.540 |
|  | [Numbness in hands and feet =1] | 0^b^ | . | . | . | . | . | . |
|  | [Body aches all over =0] | -10.099 | 6.068 | 2.770 | .096 | 4.111E-5 | 2.813E-10 | 6.008 |
|  | [Body aches all over =1] | 0^b^ | . | . | . | . | . | . |
|  | [Thirsty and fond of drinking =0] | -3.191 | 4.636 | .474 | .491 | .041 | 4.661E-6 | 363.144 |
|  | [Thirsty and fond of drinking =1] | 0^b^ | . | . | . | . | . | . |
|  | [Preference for cold drinks =0] | -23.416 | 11.869 | 3.892 | .049 | 6.766E-11 | 5.338E-21 | .858 |
|  | [Preference for cold drinks =1] | 0^b^ | . | . | . | . | . | . |
|  | [Preference for hot drinks =0] | 3.294 | 4.634 | .505 | .477 | 26.943 | .003 | 237163.959 |
|  | [Preference for hot drinks =1] | 0^b^ | . | . | . | . | . | . |
|  | [Feeling thirsty but not wanting to drink or drinking very little =0] | 1.596 | 7.132 | .050 | .823 | 4.935 | 4.190E-6 | 5812453.608 |
|  | [Feeling thirsty but not wanting to drink or drinking very little =1] | 0^b^ | . | . | . | . | . | . |
|  | [Poor appetite =0] | -1.213 | 6.015 | .041 | .840 | .297 | 2.256E-6 | 39198.498 |
|  | [Poor appetite =1] | 0^b^ | . | . | . | . | . | . |
|  | [Bitter taste in the mouth =0] | -1.095 | 5.509 | .039 | .842 | .335 | 6.848E-6 | 16352.961 |
|  | [Bitter taste in the mouth =1] | 0^b^ | . | . | . | . | . | . |
|  | [Dry mouth =0] | 5.022 | 5.421 | .858 | .354 | 151.747 | .004 | 6249673.008 |
|  | [Dry mouth =1] | 0^b^ | . | . | . | . | . | . |
|  | [Sweet tooth =0] | .822 | 5.129 | .026 | .873 | 2.275 | 9.797E-5 | 52811.962 |
|  | [Sweet tooth =1] | 0^b^ | . | . | . | . | . | . |
|  | [Preference for salty food =0] | -2.332 | 6.701 | .121 | .728 | .097 | 1.922E-7 | 49073.607 |
|  | [Preference for salty food =1] | 0^b^ | . | . | . | . | . | . |
|  | [Preference for strong tea =0] | -1.771 | 4.918 | .130 | .719 | .170 | 1.109E-5 | 2613.931 |
|  | [Preference for strong tea =1] | 0^b^ | . | . | . | . | . | . |
|  | [Love meat =0] | 3.182 | 6.437 | .244 | .621 | 24.088 | 7.985E-5 | 7266410.522 |
|  | [Love meat =1] | 0^b^ | . | . | . | . | . | . |
|  | [Long-term vegetarianism =0] | 8.099 | 10.390 | .608 | .436 | 3291.207 | 4.713E-6 | 2298503153156.435 |
|  | [Long-term vegetarianism =1] | 0^b^ | . | . | . | . | . | . |
|  | [Constipation =0] | -8.192 | 4.310 | 3.613 | .057 | .000 | 5.944E-8 | 1.290 |
|  | [Constipation =1] | 0^b^ | . | . | . | . | . | . |
|  | [Loose stools =0] | .090 | 11.711 | .000 | .994 | 1.094 | 1.177E-10 | 10178489244.243 |
|  | [Loose stools =1] | 0^b^ | . | . | . | . | . | . |
|  | [Complete valley Unmelted =0] | -32.643 | 34.552 | .893 | .345 | 6.655E-15 | 2.583E-44 | 1714342696026448.000 |
|  | [Complete valley Unmelted =1] | 0^b^ | . | . | . | . | . | . |
|  | [Morning diarrhea =0] | 16.737 | 141.646 | .014 | .906 | 18572838.019 | 5.003E-114 | 6.895E+127 |
|  | [Morning diarrhea =1] | 0^b^ | . | . | . | . | . | . |
|  | [Loose and unregulated knots =0] | -40.554 | 26.613 | 2.322 | .128 | 2.441E-18 | 5.428E-41 | 109818.342 |
|  | [Loose and unregulated knots =1] | 0^b^ | . | . | . | . | . | . |
|  | [Clear and long urine =0] | 42.468 | 148.834 | .081 | .775 | 2776610395280778800.000 | 5.696E-109 | 1.354E+145 |
|  | [Clear and long urine =1] | 0^b^ | . | . | . | . | . | . |
|  | [Yellowish urine =0] | -2.485 | 15.472 | .026 | .872 | .083 | 5.635E-15 | 1231249905273.113 |
|  | [Yellowish urine =1] | 0^b^ | . | . | . | . | . | . |
|  | [Frequency of urination =0] | 4.076 | 7.369 | .306 | .580 | 58.887 | 3.144E-5 | 110282782.605 |
|  | [Frequency of urination =1] | 0^b^ | . | . | . | . | . | . |
|  | [Burning urine =0] | 13.270 | 89.307 | .022 | .882 | 579813.550 | 5.562E-71 | 6.044E+81 |
|  | [Burning urine =1] | 0^b^ | . | . | . | . | . | . |
|  | [Incomplete urination =0] | 4.516 | 8.460 | .285 | .593 | 91.479 | 5.754E-6 | 1454371324.550 |
|  | [Incomplete urination =1] | 0^b^ | . | . | . | . | . | . |
|  | [Frequent nocturia =0] | -7.168 | 4.450 | 2.594 | .107 | .001 | 1.256E-7 | 4.731 |
|  | [Frequent nocturia =1] | 0^b^ | . | . | . | . | . | . |
|  | [Insomnia=0] | -5.447 | 5.066 | 1.156 | .282 | .004 | 2.100E-7 | 88.433 |
|  | [Insomnia=1] | 0^b^ | . | . | . | . | . | . |
|  | [Excessive sleepiness =0] | .417 | 13.961 | .001 | .976 | 1.517 | 1.985E-12 | 1159696071421.933 |
|  | [Excessive sleepiness =1] | 0^b^ | . | . | . | . | . | . |
|  | [Irritable and quick-tempered =0] | 5.270 | 4.991 | 1.115 | .291 | 194.452 | .011 | 3441843.068 |
|  | [Irritable and quick-tempered =1] | 0^b^ | . | . | . | . | . | . |
|  | [Easy concern =0] | -8.686 | 11.404 | .580 | .446 | .000 | 3.315E-14 | 861308.702 |
|  | [Easy concern =1] | 0^b^ | . | . | . | . | . | . |
|  | [Easily nervous =0] | 10.048 | 13.484 | .555 | .456 | 23104.302 | 7.697E-8 | 6934867190456546.000 |
|  | [Easily nervous =1] | 0^b^ | . | . | . | . | . | . |
|  | [Anxiety/Depression=0] | -.586 | 8.108 | .005 | .942 | .556 | 6.985E-8 | 4432019.356 |
|  | [Anxiety/Depression=1] | 0^b^ | . | . | . | . | . | . |
|  | [Prone to catching a cold =0] | -4.318 | 7.047 | .375 | .540 | .013 | 1.338E-8 | 13284.877 |
|  | [Prone to catching a cold =1] | 0^b^ | . | . | . | . | . | . |
|  | [Pale complexion =0] | -8.738 | 452.875 | .000 | .985 | .000 | .000 | .^c^ |
|  | [Pale complexion =1] | 0^b^ | . | . | . | . | . | . |
|  | [Red complexion (cheeks) =0] | 4.377 | 14.141 | .096 | .757 | 79.564 | 7.314E-11 | 86556770060168.720 |
|  | [Red complexion (cheeks) =1] | 0^b^ | . | . | . | . | . | . |
|  | [Pale red complexion =0] | 1.714 | 12.866 | .018 | .894 | 5.550 | 6.208E-11 | 496273040871.095 |
|  | [Pale red complexion =1] | 0^b^ | . | . | . | . | . | . |
|  | [Dark red complexion =0] | -3.041 | 5.656 | .289 | .591 | .048 | 7.327E-7 | 3117.000 |
|  | [Dark red complexion =1] | 0^b^ | . | . | . | . | . | . |
|  | [Redness on both cheeks =0] | 5.064 | 5.729 | .781 | .377 | 158.268 | .002 | 11907269.180 |
|  | [Redness on both cheeks =1] | 0^b^ | . | . | . | . | . | . |
|  | [Pale yellow complexion =0] | -4.329 | 4.552 | .904 | .342 | .013 | 1.759E-6 | 98.830 |
|  | [Pale yellow complexion =1] | 0^b^ | . | . | . | . | . | . |
|  | [Yellow complexion =0] | 5.245 | 4.223 | 1.543 | .214 | 189.596 | .048 | 745270.148 |
|  | [Yellow complexion =1] | 0^b^ | . | . | . | . | . | . |
|  | [Sallow complexion =0] | 2.814 | 6.519 | .186 | .666 | 16.676 | 4.708E-5 | 5906131.230 |
|  | [Sallow complexion =1] | 0^b^ | . | . | . | . | . | . |
|  | [Sallow complexion =0] | 15.514 | 8.117 | 3.653 | .056 | 5466708.511 | .674 | 44330230995219.310 |
|  | [Sallow complexion =1] | 0^b^ | . | . | . | . | . | . |
|  | [Pale complexion =0] | 1.570 | 7.050 | .050 | .824 | 4.806 | 4.799E-6 | 4811713.367 |
|  | [Pale complexion =1] | 0^b^ | . | . | . | . | . | . |
|  | [Dark complexion =0] | 20.935 | 11.456 | 3.339 | .068 | 1235474893.315 | .219 | 6974706507618765800.000 |
|  | [Dark complexion =1] | 0^b^ | . | . | . | . | . | . |
|  | [Sallow complexion =0] | 33.964 | 65.258 | .271 | .603 | 562712648747142.600 | 1.595E-41 | 1.986E+70 |
|  | [Sallow complexion =1] | 0^b^ | . | . | . | . | . | . |
|  | [Radiant complexion =0] | -7.541 | 6.247 | 1.457 | .227 | .001 | 2.558E-9 | 110.248 |
|  | [Radiant complexion =1] | 0^b^ | . | . | . | . | . | . |
|  | [Pale complexion =0] | -5.454 | 5.820 | .878 | .349 | .004 | 4.755E-8 | 384.810 |
|  | [Pale complexion =1] | 0^b^ | . | . | . | . | . | . |
|  | [Pale complexion =0] | 2.808 | 1324.748 | .000 | .998 | 16.584 | .000 | .^c^ |
|  | [Pale complexion =1] | 0^b^ | . | . | . | . | . | . |
|  | [Dark eye orbit color =0] | -3.885 | 5.115 | .577 | .447 | .021 | 9.099E-7 | 463.757 |
|  | [Dark eye orbit color =1] | 0^b^ | . | . | . | . | . | . |
|  | [Pale lip nail =0] | -6.388 | 14.246 | .201 | .654 | .002 | 1.258E-15 | 2246539747.344 |
|  | [Pale lip nail =1] | 0^b^ | . | . | . | . | . | . |
|  | [Lip nail red =0] | -9.819 | 8.784 | 1.250 | .264 | 5.440E-5 | 1.816E-12 | 1629.874 |
|  | [Lip nail red =1] | 0^b^ | . | . | . | . | . | . |
|  | [Light red lip nail =0] | -6.501 | 6.671 | .950 | .330 | .002 | 3.150E-9 | 715.886 |
|  | [Light red lip nail =1] | 0^b^ | . | . | . | . | . | . |
|  | [Dark red lip nail =0] | -4.471 | 5.331 | .704 | .402 | .011 | 3.317E-7 | 394.132 |
|  | [Dark red lip nail =1] | 0^b^ | . | . | . | . | . | . |
|  | [Dark purple lip nail =0] | -7.987 | 7.215 | 1.226 | .268 | .000 | 2.454E-10 | 470.515 |
|  | [Dark purple lip nail =1] | 0^b^ | . | . | . | . | . | . |
|  | [Lip and nail moisture =0] | 24.052 | 37.990 | .401 | .527 | 27893263849.682 | 1.283E-22 | 6.066E+42 |
|  | [Lip and nail moisture =1] | 0^b^ | . | . | . | . | . | . |
|  | [Dry lips and nails =0] | 4.776 | 2.936 | 2.645 | .104 | 118.577 | .375 | 37451.662 |
|  | [Dry lips and nails =1] | 0^b^ | . | . | . | . | . | . |
|  | [Pale red tongue =0] | -7.857 | 9.831 | .639 | .424 | .000 | 1.660E-12 | 90273.111 |
|  | [Pale red tongue =1] | 0^b^ | . | . | . | . | . | . |
|  | [Pale tongue =0] | 8.884 | 27.887 | .101 | .750 | 7216.546 | 1.321E-20 | 3941123101706286000000000000.000 |
|  | [Pale tongue =1] | 0^b^ | . | . | . | . | . | . |
|  | [Red tongue =0] | -15.476 | 9.645 | 2.575 | .109 | 1.900E-7 | 1.173E-15 | 30.792 |
|  | [Red tongue =1] | 0^b^ | . | . | . | . | . | . |
|  | [Purple Dark Tongue=0] | -8.417 | 9.384 | .804 | .370 | .000 | 2.275E-12 | 21506.535 |
|  | [Purple Dark Tongue=1] | 0^b^ | . | . | . | . | . | . |
|  | [Crimson tongue =0] | -12.806 | 11.106 | 1.330 | .249 | 2.743E-6 | 9.648E-16 | 7797.740 |
|  | [Crimson tongue =1] | 0^b^ | . | . | . | . | . | . |
|  | [Red tongue tip =0] | 7.924 | 7.035 | 1.269 | .260 | 2763.121 | .003 | 2687996433.210 |
|  | [Red tongue tip =1] | 0^b^ | . | . | . | . | . | . |
|  | [Fat tongue =0] | -24.618 | 9.766 | 6.355 | .012 | 2.035E-11 | 9.913E-20 | .004 |
|  | [Fat tongue =1] | 0^b^ | . | . | . | . | . | . |
|  | [Tooth mark tongue =0] | 4.957 | 2.740 | 3.274 | .070 | 142.143 | .662 | 30524.893 |
|  | [Tooth mark tongue =1] | 0^b^ | . | . | . | . | . | . |
|  | [Old tongue =0] | -.292 | 2.776 | .011 | .916 | .747 | .003 | 172.306 |
|  | [Old tongue =1] | 0^b^ | . | . | . | . | . | . |
|  | [Tender tongue =0] | -3.313 | 5.975 | .307 | .579 | .036 | 2.987E-7 | 4440.539 |
|  | [Tender tongue =1] | 0^b^ | . | . | . | . | . | . |
|  | [Cracked tongue =0] | 2.717 | 2.983 | .829 | .363 | 15.128 | .044 | 5237.694 |
|  | [Cracked tongue =1] | 0^b^ | . | . | . | . | . | . |
|  | [Ecchymosis tongue =0] | 3.608 | 4.967 | .527 | .468 | 36.875 | .002 | 623330.875 |
|  | [Ecchymosis tongue =1] | 0^b^ | . | . | . | . | . | . |
|  | [Point the tongue =0] | -4.550 | 4.800 | .899 | .343 | .011 | 8.679E-7 | 128.701 |
|  | [Point the tongue =1] | 0^b^ | . | . | . | . | . | . |
|  | [Thin moss =0] | -6.304 | 9.022 | .488 | .485 | .002 | 3.828E-11 | 87395.362 |
|  | [Thin moss =1] | 0^b^ | . | . | . | . | . | . |
|  | [Moss thickness =0] | -8.195 | 8.478 | .934 | .334 | .000 | 1.678E-11 | 4542.725 |
|  | [Moss thickness =1] | 0^b^ | . | . | . | . | . | . |
|  | [Tai Ni =0] | .264 | 6.205 | .002 | .966 | 1.302 | 6.801E-6 | 249267.112 |
|  | [Tai Ni =1] | 0^b^ | . | . | . | . | . | . |
|  | [Moss decay =0] | 4.165 | 14.200 | .086 | .769 | 64.392 | 5.268E-11 | 78715487401211.000 |
|  | [Moss decay =1] | 0^b^ | . | . | . | . | . | . |
|  | [Tairun =0] | 2.929 | 9.492 | .095 | .758 | 18.709 | 1.558E-7 | 2247026402.507 |
|  | [Tairun =1] | 0^b^ | . | . | . | . | . | . |
|  | [Taizao =0] | -.771 | 9.226 | .007 | .933 | .462 | 6.477E-9 | 32998720.437 |
|  | [Taizao =1] | 0^b^ | . | . | . | . | . | . |
|  | [Slippery tongue =0] | 41.640 | 49.827 | .698 | .403 | 1213180520472308220.000 | 4.689E-25 | 3.139E+60 |
|  | [Slippery tongue =1] | 0^b^ | . | . | . | . | . | . |
|  | [Less moss =0] | -11.086 | 8.206 | 1.825 | .177 | 1.532E-5 | 1.585E-12 | 148.063 |
|  | [Less moss =1] | 0^b^ | . | . | . | . | . | . |
|  | [Mirror tongue =0] | 44.568 | 28.831 | 2.390 | .122 | 22668875353094090000.000 | 6.520E-6 | 7.881E+43 |
|  | [Mirror tongue =1] | 0^b^ | . | . | . | . | . | . |
|  | [Full tongue coating =0] | -2.431 | 3.459 | .494 | .482 | .088 | .000 | 77.258 |
|  | [Full tongue coating =1] | 0^b^ | . | . | . | . | . | . |
|  | [Moss peeling =0] | -30.322 | 4.350 | 48.586 | .000 | 6.781E-14 | 1.344E-17 | 3.421E-10 |
|  | [Moss peeling =1] | 0^b^ | . | . | . | . | . | . |
|  | [White coating on the tongue =0] | 25.200 | 30.942 | .663 | .415 | 87985467321.843 | 4.045E-16 | 1.914E+37 |
|  | [White coating on the tongue =1] | 0^b^ | . | . | . | . | . | . |
|  | [Yellow coating on the tongue =0] | 27.579 | 31.107 | .786 | .375 | 948874517487.933 | 3.151E-15 | 2.857E+38 |
|  | [Yellow coating on the tongue =1] | 0^b^ | . | . | . | . | . | . |
|  | [Yellow and white coating on the tongue =0] | 30.483 | 31.293 | .949 | .330 | 17320623115747.568 | 3.998E-14 | 7.504E+39 |
|  | [Yellow and white coating on the tongue =1] | 0^b^ | . | . | . | . | . | . |
|  | [Grayish-black coating on the tongue =0] | 47.875 | 33.211 | 2.078 | .149 | 619303972354939800000.000 | 3.333E-8 | 1.151E+49 |
|  | [Grayish-black coating on the tongue =1] | 0^b^ | . | . | . | . | . | . |
|  | [pulse float =0] | -8.718 | 5.098 | 2.924 | .087 | .000 | 7.486E-9 | 3.576 |
|  | [pulse float =1] | 0^b^ | . | . | . | . | . | . |
|  | [pulse depth =0] | -5.698 | 4.227 | 1.817 | .178 | .003 | 8.463E-7 | 13.287 |
|  | [pulse depth =1] | 0^b^ | . | . | . | . | . | . |
|  | [Pulse slow =0] | -8.693 | 10.381 | .701 | .402 | .000 | 2.445E-13 | 115210.516 |
|  | [Pulse slow =1] | 0^b^ | . | . | . | . | . | . |
|  | [Pulse fat =0] | -6.265 | 3.754 | 2.785 | .095 | .002 | 1.212E-6 | 2.984 |
|  | [Pulse fat =1] | 0^b^ | . | . | . | . | . | . |
|  | [ Pulse flood =0] | 37.915 | 471.565 | .006 | .936 | 29260548484251336.000 | .000 | .^c^ |
|  | [Pulse flood =1] | 0^b^ | . | . | . | . | . | . |
|  | [ pulse fineness =0] | -7.897 | 5.395 | 2.143 | .143 | .000 | 9.501E-9 | 14.546 |
|  | [ pulse fineness =1] | 0^b^ | . | . | . | . | . | . |
|  | [Weak pulse=0] | -6.928 | 9.501 | .532 | .466 | .001 | 8.013E-12 | 119934.358 |
|  | [Weak pulse=1] | 0^b^ | . | . | . | . | . | . |
|  | [pulse slippery =0] | .659 | 3.807 | .030 | .863 | 1.933 | .001 | 3360.742 |
|  | [pulse slippery =1] | 0^b^ | . | . | . | . | . | . |
|  | [pulse astringency =0] | 12.081 | 9.267 | 1.700 | .192 | 176455.461 | .002 | 13627823805406.701 |
|  | [pulse astringency =1] | 0^b^ | . | . | . | . | . | . |
|  | [pulse string =0] | -5.329 | 3.939 | 1.830 | .176 | .005 | 2.151E-6 | 10.938 |
|  | [pulse string =1] | 0^b^ | . | . | . | . | . | . |
|  | [pulse tightness =0] | 30.599 | 29.176 | 1.100 | .294 | 19446517211965.160 | 2.846E-12 | 1.329E+38 |
|  | [pulse tightness =1] | 0^b^ | . | . | . | . | . | . |
|  | [pulse knot representation =0] | 3.376 | 6.350 | .283 | .595 | 29.256 | .000 | 7440618.370 |
|  | [pulse knot representation =1] | 0^b^ | . | . | . | . | . | . |
| Qi stagnation and blood stasis syndrome | Intercept | -196.311 | 1447.848 | .018 | .892 |  |  |  |
|  | [Palpitations =0] | 5.471 | 4.415 | 1.536 | .215 | 237.769 | .041 | 1362786.617 |
|  | [Palpitations =1] | 0^b^ | . | . | . | . | . | . |
|  | [Chest tightness =0] | 4.831 | 4.710 | 1.052 | .305 | 125.294 | .012 | 1280540.984 |
|  | [Chest tightness =1] | 0^b^ | . | . | . | . | . | . |
|  | [Shortness of breath =0] | 3.489 | 5.268 | .439 | .508 | 32.754 | .001 | 997735.490 |
|  | [Shortness of breath =1] | 0^b^ | . | . | . | . | . | . |
|  | [Chest pain =0] | 33.191 | 16.860 | 3.875 | .049 | 259741125757749.340 | 1.157 | 58319032050352625000000000000.000 |
|  | [Chest pain =1] | 0^b^ | . | . | . | . | . | . |
|  | [Precordial area =0] | -7.809 | 9.501 | .676 | .411 | .000 | 3.325E-12 | 49605.031 |
|  | [Precordial area =1] | 0^b^ | . | . | . | . | . | . |
|  | [Zhongfu =0] | -4.218 | 11.484 | .135 | .713 | .015 | 2.471E-12 | 87811312.198 |
|  | [Zhongfu =1] | 0^b^ | . | . | . | . | . | . |
|  | [Chest flap =0] | -.713 | 9.823 | .005 | .942 | .490 | 2.133E-9 | 112670752.630 |
|  | [Chest flap =1] | 0^b^ | . | . | . | . | . | . |
|  | [Pain on the inner side of the shoulder and arm =0] | 45.850 | 12.405 | 13.661 | .000 | 81742516535527570000.000 | 2254594735.397 | 2963654134667775500000000000000.000 |
|  | [Pain on the inner side of the shoulder and arm =1] | 0^b^ | . | . | . | . | . | . |
|  | [Pain has no fixed location =0] | -.652 | 19.030 | .001 | .973 | .521 | 3.301E-17 | 8217411251366766.000 |
|  | [Pain has no fixed location =1] | 0^b^ | . | . | . | . | . | . |
|  | [The pain has a fixed location =0] | 9.656 | 14.117 | .468 | .494 | 15616.305 | 1.504E-8 | 16217880201321698.000 |
|  | [The pain has a fixed location =1] | 0^b^ | . | . | . | . | . | . |
|  | [Stabbing pain =0] | -11.615 | 9.853 | 1.390 | .238 | 9.029E-6 | 3.707E-14 | 2199.190 |
|  | [Stabbing pain =1] | 0^b^ | . | . | . | . | . | . |
|  | [Dull pain =0] | -1.702 | 9.949 | .029 | .864 | .182 | 6.195E-10 | 53655735.739 |
|  | [Dull pain =1] | 0^b^ | . | . | . | . | . | . |
|  | [Distending pain =0] | -57.463 | 24.333 | 5.577 | .018 | 1.107E-25 | 2.145E-46 | 5.711E-5 |
|  | [Distending pain =1] | 0^b^ | . | . | . | . | . | . |
|  | [Colic =0] | -8.899 | 13.791 | .416 | .519 | .000 | 2.491E-16 | 74790132.190 |
|  | [Colic =1] | 0^b^ | . | . | . | . | . | . |
|  | [Dull pain =0] | -14.503 | 14.537 | .995 | .318 | 5.028E-7 | 2.126E-19 | 1189261.567 |
|  | [Dull pain =1] | 0^b^ | . | . | . | . | . | . |
|  | [Post-activity increase =0] | 2.654 | 6.096 | .190 | .663 | 14.209 | 9.193E-5 | 2195957.930 |
|  | [Post-activity increase =1] | 0^b^ | . | . | . | . | . | . |
|  | [Induced by unfulfilled emotions =0] | -29.397 | 9.530 | 9.516 | .002 | 1.711E-13 | 1.323E-21 | 2.211E-5 |
|  | [Induced by unfulfilled emotions =1] | 0^b^ | . | . | . | . | . | . |
|  | [Induced by drinking and overeating =0]] | -37.973 | 33.080 | 1.318 | .251 | 3.226E-17 | 2.242E-45 | 464058419562.478 |
|  | [Induced by drinking and overeating =1] | 0^b^ | . | . | . | . | . | . |
|  | [Induced by rainy days =0] | 12.944 | 9.598 | 1.819 | .177 | 418448.381 | .003 | 61898586071017.220 |
|  | [Induced by rainy days =1] | 0^b^ | . | . | . | . | . | . |
|  | [Sudden cold weather or sudden exposure to cold wind triggers =0] | 8.002 | 17.323 | .213 | .644 | 2987.127 | 5.367E-12 | 1662412738722214140.000 |
|  | [Sudden cold weather or sudden exposure to cold wind triggers =1] | 0^b^ | . | . | . | . | . | . |
|  | [Relief after rest =0] | 7.687 | 3.640 | 4.459 | .035 | 2178.893 | 1.736 | 2734003.519 |
|  | [Relief after rest =1] | 0^b^ | . | . | . | . | . | . |
|  | [Edema =0] | 2.966 | 8.396 | .125 | .724 | 19.422 | 1.386E-6 | 272113714.638 |
|  | [Edema =1] | 0^b^ | . | . | . | . | . | . |
|  | [Weak and talkative =0] | 2.475 | 4.717 | .275 | .600 | 11.880 | .001 | 123123.948 |
|  | [Weak and talkative =1] | 0^b^ | . | . | . | . | . | . |
|  | [Feeling annoyed =0] | -10.982 | 6.174 | 3.164 | .075 | 1.701E-5 | 9.460E-11 | 3.060 |
|  | [Feeling annoyed =1] | 0^b^ | . | . | . | . | . | . |
|  | [Forgetfulness =0] | -6.468 | 7.257 | .794 | .373 | .002 | 1.033E-9 | 2332.329 |
|  | [Forgetfulness =1] | 0^b^ | . | . | . | . | . | . |
|  | [Heat intolerance =0] | -5.290 | 7.000 | .571 | .450 | .005 | 5.543E-9 | 4582.344 |
|  | [Heat intolerance =1] | 0^b^ | . | . | . | . | . | . |
|  | [Fear of cold =0] | -4.184 | 8.423 | .247 | .619 | .015 | 1.031E-9 | 225313.791 |
|  | [Fear of cold =1] | 0^b^ | . | . | . | . | . | . |
|  | [Cold limbs =0] | -9.848 | 7.848 | 1.575 | .210 | 5.284E-5 | 1.102E-11 | 253.252 |
|  | [Cold limbs =1] | 0^b^ | . | . | . | . | . | . |
|  | [Hot and humid =0] | -.508 | 13.324 | .001 | .970 | .601 | 2.741E-12 | 131975935323.870 |
|  | [Hot and humid =1] | 0^b^ | . | . | . | . | . | . |
|  | [Hot palms and soles =0] | -8.907 | 16.226 | .301 | .583 | .000 | 2.090E-18 | 8779028179.830 |
|  | [Hot palms and soles =1] | 0^b^ | . | . | . | . | . | . |
|  | [Spontaneous sweating =0] | -3.273 | 5.503 | .354 | .552 | .038 | 7.834E-7 | 1832.502 |
|  | [Spontaneous sweating =1] | 0^b^ | . | . | . | . | . | . |
|  | [Night sweats =0] | .184 | 9.636 | .000 | .985 | 1.202 | 7.543E-9 | 191447204.211 |
|  | [Night sweats =1] | 0^b^ | . | . | . | . | . | . |
|  | [Dizziness =0] | -2.960 | 6.177 | .230 | .632 | .052 | 2.862E-7 | 9381.461 |
|  | [Dizziness =1] | 0^b^ | . | . | . | . | . | . |
|  | [Dry eyes =0] | -5.965 | 5.182 | 1.325 | .250 | .003 | 9.976E-8 | 66.079 |
|  | [Dry eyes =1] | 0^b^ | . | . | . | . | . | . |
|  | [Tinnitus =0] | 6.967 | 5.300 | 1.728 | .189 | 1061.139 | .033 | 34426825.819 |
|  | [Tinnitus =1] | 0^b^ | . | . | . | . | . | . |
|  | [Mouth ulcers =0] | 9.695 | 7.926 | 1.496 | .221 | 16234.530 | .003 | 90619008744.268 |
|  | [Mouth ulcers =1] | 0^b^ | . | . | . | . | . | . |
|  | [Swollen and painful gums =0] | -2.286 | 15.560 | .022 | .883 | .102 | 5.787E-15 | 1785779004408.656 |
|  | [Swollen and painful gums =1] | 0^b^ | . | . | . | . | . | . |
|  | [Cough =0] | -7.938 | 8.308 | .913 | .339 | .000 | 3.023E-11 | 4212.034 |
|  | [Cough =1] | 0^b^ | . | . | . | . | . | . |
|  | [White and thin phlegm =0] | -4.279 | 11.629 | .135 | .713 | .014 | 1.750E-12 | 109704772.839 |
|  | [White and thin phlegm =1] | 0^b^ | . | . | . | . | . | . |
|  | [White thick phlegm =0] | 28.035 | 18.370 | 2.329 | .127 | 1498197487260.078 | .000 | 6492108395923610000000000000.000 |
|  | [White thick phlegm =1] | 0^b^ | . | . | . | . | . | . |
|  | [Yellow sticky phlegm =0] | -4.911 | 22.868 | .046 | .830 | .007 | 2.523E-22 | 215174847045700096.000 |
|  | [Yellow sticky phlegm =1] | 0^b^ | . | . | . | . | . | . |
|  | Frothy sputum =0] | 40.258 | 19.127 | 4.430 | .035 | 304776982166019650.000 | 15.952 | 5823142133188270000000000000000000.000 |
|  | [Frothy sputum =1] | 0^b^ | . | . | . | . | . | . |
|  | [Stomach pain prefers warmth and pressure =0] | -7.067 | 16.276 | .189 | .664 | .001 | 1.193E-17 | 60964851250.901 |
|  | [Stomach pain prefers warmth and pressure =1] | 0^b^ | . | . | . | . | . | . |
|  | [Good Sigh =0] | -6.756 | 12.748 | .281 | .596 | .001 | 1.640E-14 | 82648037.030 |
|  | [Good Sigh =1] | 0^b^ | . | . | . | . | . | . |
|  | [Costal distension =0] | -29.739 | 13.974 | 4.529 | .033 | 1.215E-13 | 1.548E-25 | .095 |
|  | [Costal distension =1] | 0^b^ | . | . | . | . | . | . |
|  | [Weakness in the waist and knees =0] | 2.282 | 4.919 | .215 | .643 | 9.799 | .001 | 150689.971 |
|  | [Weakness in the waist and knees =1] | 0^b^ | . | . | . | . | . | . |
|  | [Numbness in hands and feet =0] | 3.242 | 5.694 | .324 | .569 | 25.578 | .000 | 1796768.598 |
|  | [Numbness in hands and feet =1] | 0^b^ | . | . | . | . | . | . |
|  | [Body aches all over =0] | -6.983 | 8.400 | .691 | .406 | .001 | 6.560E-11 | 13104.608 |
|  | [Body aches all over =1] | 0^b^ | . | . | . | . | . | . |
|  | [Thirsty and fond of drinking =0] | 10.427 | 6.793 | 2.356 | .125 | 33765.535 | .056 | 20434839941.058 |
|  | [Thirsty and fond of drinking =1] | 0^b^ | . | . | . | . | . | . |
|  | [Preference for cold drinks =0] | -16.158 | 13.229 | 1.492 | .222 | 9.608E-8 | 5.272E-19 | 17509.404 |
|  | [Preference for cold drinks =1] | 0^b^ | . | . | . | . | . | . |
|  | [Preference for hot drinks =0] | -.081 | 5.543 | .000 | .988 | .922 | 1.766E-5 | 48155.631 |
|  | [Preference for hot drinks =1] | 0^b^ | . | . | . | . | . | . |
|  | [Feeling thirsty but not wanting to drink or drinking very little =0] | 5.728 | 8.497 | .454 | .500 | 307.224 | 1.798E-5 | 5248922431.256 |
|  | [Feeling thirsty but not wanting to drink or drinking very little =1] | 0^b^ | . | . | . | . | . | . |
|  | [Poor appetite =0] | 5.863 | 7.708 | .579 | .447 | 351.811 | 9.660E-5 | 1281209311.822 |
|  | [Poor appetite =1] | 0^b^ | . | . | . | . | . | . |
|  | [Bitter taste in the mouth =0] | -1.427 | 6.493 | .048 | .826 | .240 | 7.142E-7 | 80670.044 |
|  | [Bitter taste in the mouth =1] | 0^b^ | . | . | . | . | . | . |
|  | [Dry mouth =0] | 5.083 | 6.229 | .666 | .415 | 161.261 | .001 | 32359708.075 |
|  | [Dry mouth =1] | 0^b^ | . | . | . | . | . | . |
|  | [Sweet tooth =0] | 5.620 | 7.107 | .625 | .429 | 275.953 | .000 | 309204461.973 |
|  | [Sweet tooth =1] | 0^b^ | . | . | . | . | . | . |
|  | [Preference for salty food =0] | 8.349 | 9.399 | .789 | .374 | 4226.971 | 4.222E-5 | 423198428519.440 |
|  | [Preference for salty food =1] | 0^b^ | . | . | . | . | . | . |
|  | [Preference for strong tea =0] | 1.427 | 6.345 | .051 | .822 | 4.168 | 1.656E-5 | 1048931.406 |
|  | [Preference for strong tea =1] | 0^b^ | . | . | . | . | . | . |
|  | [Love meat =0] | 6.943 | 8.829 | .618 | .432 | 1036.280 | 3.163E-5 | 33954484257.993 |
|  | [Love meat =1] | 0^b^ | . | . | . | . | . | . |
|  | [Long-term vegetarianism =0] | 16.103 | 13.459 | 1.432 | .232 | 9852657.540 | 3.446E-5 | 2816933554189237200.000 |
|  | [Long-term vegetarianism =1] | 0^b^ | . | . | . | . | . | . |
|  | [Constipation =0] | -14.653 | 5.391 | 7.389 | .007 | 4.326E-7 | 1.116E-11 | .017 |
|  | [Constipation =1] | 0^b^ | . | . | . | . | . | . |
|  | [Loose stools =0] | 5.291 | 13.940 | .144 | .704 | 198.563 | 2.703E-10 | 145889655465148.440 |
|  | [Loose stools =1] | 0^b^ | . | . | . | . | . | . |
|  | [Complete valley Unmelted =0] | -21.907 | 37.245 | .346 | .556 | 3.061E-10 | 6.067E-42 | 15441649647078326000000.000 |
|  | [Complete valley Unmelted =1] | 0^b^ | . | . | . | . | . | . |
|  | [Morning diarrhea =0] | 7.612 | 103.889 | .005 | .942 | 2021.383 | 7.503E-86 | 5.446E+91 |
|  | [Morning diarrhea =1] | 0^b^ | . | . | . | . | . | . |
|  | [Loose and unregulated knots =0] | -43.131 | 31.148 | 1.917 | .166 | 1.856E-19 | 5.697E-46 | 60459216.996 |
|  | [Loose and unregulated knots =1] | 0^b^ | . | . | . | . | . | . |
|  | [Clear and long urine =0] | 28.711 | 370.591 | .006 | .938 | 2943263819272.930 | 1.051E-303 | .^c^ |
|  | [Clear and long urine =1] | 0^b^ | . | . | . | . | . | . |
|  | [Yellowish urine =0] | 38.726 | 28.738 | 1.816 | .178 | 65830835539982960.000 | 2.273E-8 | 1.906E+41 |
|  | [Yellowish urine =1] | 0^b^ | . | . | . | . | . | . |
|  | [Frequency of urination =0] | 5.191 | 8.062 | .415 | .520 | 179.682 | 2.468E-5 | 1308054019.040 |
|  | [Frequency of urination =1] | 0^b^ | . | . | . | . | . | . |
|  | [Burning urine =0] | -13.059 | 91.519 | .020 | .887 | 2.130E-6 | 2.676E-84 | 1.695E+72 |
|  | [Burning urine =1] | 0^b^ | . | . | . | . | . | . |
|  | [Incomplete urination =0] | 21.361 | 13.559 | 2.482 | .115 | 1891582732.916 | .005 | 657470476997846900000.000 |
|  | [Incomplete urination =1] | 0^b^ | . | . | . | . | . | . |
|  | [Frequent nocturia =0] | -9.038 | 5.355 | 2.848 | .091 | .000 | 3.284E-9 | 4.300 |
|  | [Frequent nocturia =1] | 0^b^ | . | . | . | . | . | . |
|  | [Insomnia=0] | -8.776 | 5.444 | 2.599 | .107 | .000 | 3.589E-9 | 6.647 |
|  | [Insomnia=1] | 0^b^ | . | . | . | . | . | . |
|  | [Excessive sleepiness =0] | -4.740 | 18.783 | .064 | .801 | .009 | 8.977E-19 | 85079798366992.250 |
|  | [Excessive sleepiness =1] | 0^b^ | . | . | . | . | . | . |
|  | [Irritable and quick-tempered =0] | -16.536 | 6.051 | 7.468 | .006 | 6.582E-8 | 4.651E-13 | .009 |
|  | [Irritable and quick-tempered =1] | 0^b^ | . | . | . | . | . | . |
|  | [Easy concern =0] | -13.058 | 12.235 | 1.139 | .286 | 2.132E-6 | 8.203E-17 | 55428.446 |
|  | [Easy concern =1] | 0^b^ | . | . | . | . | . | . |
|  | [Easily nervous =0] | -5.254 | 14.631 | .129 | .720 | .005 | 1.836E-15 | 14880245000.589 |
|  | [Easily nervous =1] | 0^b^ | . | . | . | . | . | . |
|  | [Anxiety/Depression=0] | -14.997 | 9.348 | 2.574 | .109 | 3.067E-7 | 3.385E-15 | 27.792 |
|  | [Anxiety/Depression=1] | 0^b^ | . | . | . | . | . | . |
|  | [Prone to catching a cold =0] | 2.038 | 10.487 | .038 | .846 | 7.674 | 9.095E-9 | 6474448609.436 |
|  | [Prone to catching a cold =1] | 0^b^ | . | . | . | . | . | . |
|  | [Pale complexion =0] | -4.992 | 461.733 | .000 | .991 | .007 | .000 | .^c^ |
|  | [Pale complexion =1] | 0^b^ | . | . | . | . | . | . |
|  | [Red complexion (cheeks) =0] | -5.010 | 15.219 | .108 | .742 | .007 | 7.403E-16 | 60098681064.137 |
|  | [Red complexion (cheeks) =1] | 0^b^ | . | . | . | . | . | . |
|  | [Pale red complexion =0] | -.216 | 14.415 | .000 | .988 | .806 | 4.329E-13 | 1499426502042.734 |
|  | [Pale red complexion =1] | 0^b^ | . | . | . | . | . | . |
|  | [Dark red complexion =0] | -16.421 | 8.342 | 3.875 | .049 | 7.384E-8 | 5.850E-15 | .932 |
|  | [Dark red complexion =1] | 0^b^ | . | . | . | . | . | . |
|  | [Redness on both cheeks =0] | 6.266 | 7.250 | .747 | .387 | 526.204 | .000 | 779754487.883 |
|  | [Redness on both cheeks =1] | 0^b^ | . | . | . | . | . | . |
|  | [Pale yellow complexion =0] | 2.180 | 5.909 | .136 | .712 | 8.842 | 8.254E-5 | 947230.519 |
|  | [Pale yellow complexion =1] | 0^b^ | . | . | . | . | . | . |
|  | [Yellow complexion =0] | 6.636 | 5.445 | 1.485 | .223 | 761.936 | .018 | 32853400.412 |
|  | [Yellow complexion =1] | 0^b^ | . | . | . | . | . | . |
|  | [Sallow complexion =0] | .455 | 7.498 | .004 | .952 | 1.577 | 6.542E-7 | 3800031.364 |
|  | [Sallow complexion =1] | 0^b^ | . | . | . | . | . | . |
|  | [Sallow complexion =0] | 14.427 | 8.981 | 2.581 | .108 | 1843784.497 | .042 | 81313260375341.840 |
|  | [Sallow complexion =1] | 0^b^ | . | . | . | . | . | . |
|  | [Pale complexion =0] | 2.943 | 10.191 | .083 | .773 | 18.972 | 4.014E-8 | 8966511964.992 |
|  | [Pale complexion =1] | 0^b^ | . | . | . | . | . | . |
|  | [Dark complexion =0] | 43.034 | 667.990 | .004 | .949 | 4889568070453003300.000 | .000 | .^c^ |
|  | [Dark complexion =1] | 0^b^ | . | . | . | . | . | . |
|  | [Sallow complexion =0] | 26.287 | 36.862 | .509 | .476 | 260752756988.064 | 1.094E-20 | 6.218E+42 |
|  | [Sallow complexion =1] | 0^b^ | . | . | . | . | . | . |
|  | [Radiant complexion =0] | -7.480 | 9.095 | .676 | .411 | .001 | 1.023E-11 | 31112.674 |
|  | [Radiant complexion =1] | 0^b^ | . | . | . | . | . | . |
|  | [Pale complexion =0] | -10.777 | 8.010 | 1.811 | .178 | 2.087E-5 | 3.175E-12 | 137.162 |
|  | [Pale complexion =1] | 0^b^ | . | . | . | . | . | . |
|  | [Pale complexion =0] | 20.601 | 1033.456 | .000 | .984 | 884858990.518 | .000 | .^c^ |
|  | [Pale complexion =1] | 0^b^ | . | . | . | . | . | . |
|  | [Dark eye orbit color =0] | -11.214 | 5.952 | 3.550 | .060 | 1.348E-5 | 1.157E-10 | 1.569 |
|  | [Dark eye orbit color =1] | 0^b^ | . | . | . | . | . | . |
|  | [Pale lip nail =0] | 8.165 | 21.116 | .150 | .699 | 3516.639 | 3.731E-15 | 3314434355476756000000.000 |
|  | [Pale lip nail =1] | 0^b^ | . | . | . | . | . | . |
|  | [Lip nail red =0] | -7.432 | 17.177 | .187 | .665 | .001 | 1.417E-18 | 247554766319.077 |
|  | [Lip nail red =1] | 0^b^ | . | . | . | . | . | . |
|  | [Light red lip nail =0] | -3.075 | 16.650 | .034 | .853 | .046 | 3.105E-16 | 6868601849543.393 |
|  | [Light red lip nail =1] | 0^b^ | . | . | . | . | . | . |
|  | [Dark red lip nail =0] | -12.040 | 16.098 | .559 | .455 | 5.902E-6 | 1.170E-19 | 297790716.112 |
|  | [Dark red lip nail =1] | 0^b^ | . | . | . | . | . | . |
|  | [Dark purple lip nail =0] | -15.223 | 16.107 | .893 | .345 | 2.449E-7 | 4.775E-21 | 12557382.566 |
|  | [Dark purple lip nail =1] | 0^b^ | . | . | . | . | . | . |
|  | [Lip and nail moisture =0] | 51.867 | 60.760 | .729 | .393 | 33533745724419484000000.000 | 6.403E-30 | 1.756E+74 |
|  | [Lip and nail moisture =1] | 0^b^ | . | . | . | . | . | . |
|  | [Dry lips and nails =0] | 4.894 | 4.293 | 1.299 | .254 | 133.440 | .030 | 601852.953 |
|  | [Dry lips and nails =1] | 0^b^ | . | . | . | . | . | . |
|  | [Pale red tongue =0] | -1.935 | 10.767 | .032 | .857 | .144 | 9.889E-11 | 211095318.300 |
|  | [Pale red tongue =1] | 0^b^ | . | . | . | . | . | . |
|  | [Pale tongue =0] | 9.342 | 35.457 | .069 | .792 | 11408.533 | 7.524E-27 | 17299380277434817000000000000000000.000 |
|  | [Pale tongue =1] | 0^b^ | . | . | . | . | . | . |
|  | [Red tongue =0] | -7.242 | 10.710 | .457 | .499 | .001 | 5.476E-13 | 935093.922 |
|  | [Red tongue =1] | 0^b^ | . | . | . | . | . | . |
|  | [Purple Dark Tongue=0] | -4.447 | 10.497 | .179 | .672 | .012 | 1.361E-11 | 10088652.064 |
|  | [Purple Dark Tongue=1] | 0^b^ | . | . | . | . | . | . |
|  | [Crimson tongue =0] | -23.512 | 13.150 | 3.197 | .074 | 6.147E-11 | 3.942E-22 | 9.585 |
|  | [Crimson tongue =1] | 0^b^ | . | . | . | . | . | . |
|  | [Red tongue tip =0] | 4.287 | 8.246 | .270 | .603 | 72.712 | 6.963E-6 | 759363845.065 |
|  | [Red tongue tip =1] | 0^b^ | . | . | . | . | . | . |
|  | [Fat tongue =0] | -19.684 | 10.871 | 3.278 | .070 | 2.826E-9 | 1.575E-18 | 5.069 |
|  | [Fat tongue =1] | 0^b^ | . | . | . | . | . | . |
|  | [Tooth mark tongue =0] | 6.167 | 3.874 | 2.535 | .111 | 476.981 | .240 | 946365.910 |
|  | [Tooth mark tongue =1] | 0^b^ | . | . | . | . | . | . |
|  | [Old tongue =0] | 2.436 | 3.994 | .372 | .542 | 11.432 | .005 | 28686.413 |
|  | [Old tongue =1] | 0^b^ | . | . | . | . | . | . |
|  | [Tender tongue =0] | -5.377 | 7.595 | .501 | .479 | .005 | 1.586E-9 | 13475.589 |
|  | [Tender tongue =1] | 0^b^ | . | . | . | . | . | . |
|  | [Cracked tongue =0] | -.304 | 4.045 | .006 | .940 | .738 | .000 | 2046.377 |
|  | [Cracked tongue =1] | 0^b^ | . | . | . | . | . | . |
|  | [Ecchymosis tongue =0] | 12.222 | 6.836 | 3.197 | .074 | 203262.416 | .308 | 133968091066.854 |
|  | [Ecchymosis tongue =1] | 0^b^ | . | . | . | . | . | . |
|  | [Point the tongue =0] | .562 | 6.196 | .008 | .928 | 1.754 | 9.327E-6 | 330029.425 |
|  | [Point the tongue =1] | 0^b^ | . | . | . | . | . | . |
|  | [Thin moss =0] | 9.151 | 10.336 | .784 | .376 | 9422.912 | 1.499E-5 | 5923549817177.631 |
|  | [Thin moss =1] | 0^b^ | . | . | . | . | . | . |
|  | [Moss thickness =0] | 4.072 | 10.421 | .153 | .696 | 58.647 | 7.909E-8 | 43486851600.537 |
|  | [Moss thickness =1] | 0^b^ | . | . | . | . | . | . |
|  | [Tai Ni =0] | 4.659 | 6.743 | .477 | .490 | 105.521 | .000 | 57956983.245 |
|  | [Tai Ni =1] | 0^b^ | . | . | . | . | . | . |
|  | [Moss decay =0] | 6.828 | 32.588 | .044 | .834 | 923.635 | 1.685E-25 | 5063444233462390000000000000000.000 |
|  | [Moss decay =1] | 0^b^ | . | . | . | . | . | . |
|  | [Tairun =0] | 7.648 | 10.911 | .491 | .483 | 2095.444 | 1.081E-6 | 4063458609947.018 |
|  | [Tairun =1] | 0^b^ | . | . | . | . | . | . |
|  | [Taizao =0] | -1.158 | 10.714 | .012 | .914 | .314 | 2.385E-10 | 413712514.205 |
|  | [Taizao =1] | 0^b^ | . | . | . | . | . | . |
|  | [Slippery tongue =0] | 22.867 | 46.008 | .247 | .619 | 8529016501.832 | 5.876E-30 | 1.238E+49 |
|  | [Slippery tongue =1] | 0^b^ | . | . | . | . | . | . |
|  | [Less moss =0] | -19.250 | 8.915 | 4.663 | .031 | 4.363E-9 | 1.126E-16 | .169 |
|  | [Less moss =1] | 0^b^ | . | . | . | . | . | . |
|  | [Mirror tongue =0] | 48.293 | 34.492 | 1.960 | .161 | 940417700827992200000.000 | 4.108E-9 | 2.153E+50 |
|  | [Mirror tongue =1] | 0^b^ | . | . | . | . | . | . |
|  | [Full tongue coating =0] | -.833 | 4.416 | .036 | .850 | .435 | 7.577E-5 | 2493.227 |
|  | [Full tongue coating =1] | 0^b^ | . | . | . | . | . | . |
|  | [Moss peeling =0] | -14.307 | 10.397 | 1.893 | .169 | 6.119E-7 | 8.645E-16 | 433.140 |
|  | [Moss peeling =1] | 0^b^ | . | . | . | . | . | . |
|  | [White coating on the tongue =0] | 25.504 | 33.176 | .591 | .442 | 119223018949.835 | 6.864E-18 | 2.071E+39 |
|  | [White coating on the tongue =1] | 0^b^ | . | . | . | . | . | . |
|  | [Yellow coating on the tongue =0] | 23.322 | 33.476 | .485 | .486 | 13448512514.356 | 4.302E-19 | 4.204E+38 |
|  | [Yellow coating on the tongue =1] | 0^b^ | . | . | . | . | . | . |
|  | [Yellow and white coating on the tongue =0] | 25.469 | 34.065 | .559 | .455 | 115050324260.283 | 1.160E-18 | 1.141E+40 |
|  | [Yellow and white coating on the tongue =1] | 0^b^ | . | . | . | . | . | . |
|  | [Grayish-black coating on the tongue =0] | 52.538 | 61.246 | .736 | .391 | 65588989720275400000000.000 | 4.836E-30 | 8.895E+74 |
|  | [Grayish-black coating on the tongue =1] | 0^b^ | . | . | . | . | . | . |
|  | [pulse float =0] | -5.081 | 5.501 | .853 | .356 | .006 | 1.292E-7 | 299.053 |
|  | [pulse float =1] | 0^b^ | . | . | . | . | . | . |
|  | [pulse depth =0] | -8.467 | 5.854 | 2.092 | .148 | .000 | 2.189E-9 | 20.205 |
|  | [pulse depth =1] | 0^b^ | . | . | . | . | . | . |
|  | [Pulse slow =0] | -14.844 | 12.423 | 1.428 | .232 | 3.575E-7 | 9.516E-18 | 13428.449 |
|  | [Pulse slow =1] | 0^b^ | . | . | . | . | . | . |
|  | [Pulse fat =0] | -2.291 | 4.459 | .264 | .607 | .101 | 1.621E-5 | 631.158 |
|  | [Pulse fat =1] | 0^b^ | . | . | . | . | . | . |
|  | [ Pulse flood =0] | 26.075 | 526.714 | .002 | .961 | 210980582549.843 | .000 | .^c^ |
|  | [Pulse flood =1] | 0^b^ | . | . | . | . | . | . |
|  | [ pulse fineness =0] | -3.295 | 6.354 | .269 | .604 | .037 | 1.447E-7 | 9492.056 |
|  | [ pulse fineness =1] | 0^b^ | . | . | . | . | . | . |
|  | [Weak pulse=0] | 3.835 | 12.957 | .088 | .767 | 46.293 | 4.327E-10 | 4952972796101.486 |
|  | [Weak pulse=1] | 0^b^ | . | . | . | . | . | . |
|  | [pulse slippery =0] | 3.688 | 5.019 | .540 | .462 | 39.950 | .002 | 747512.008 |
|  | [pulse slippery =1] | 0^b^ | . | . | . | . | . | . |
|  | [pulse astringency =0] | -.588 | 11.687 | .003 | .960 | .556 | 6.259E-11 | 4931301417.227 |
|  | [pulse astringency =1] | 0^b^ | . | . | . | . | . | . |
|  | [pulse string =0] | -18.163 | 4.675 | 15.094 | .000 | 1.294E-8 | 1.356E-12 | .000 |
|  | [pulse string =1] | 0^b^ | . | . | . | . | . | . |
|  | [pulse tightness =0] | 7.303 | 24.082 | .092 | .762 | 1484.130 | 4.704E-18 | 468270081763102700000000.000 |
|  | [pulse tightness =1] | 0^b^ | . | . | . | . | . | . |
|  | [pulse knot representation =0] | 4.907 | 7.042 | .486 | .486 | 135.219 | .000 | 133401644.836 |
|  | [pulse knot representation =1] | 0^b^ | . | . | . | . | . | . |
| Phlegm turbidity obstruction syndrome | Intercept | -87.196 | 1466.854 | .004 | .953 |  |  |  |
|  | [Palpitations =0] | 7.297 | 3.818 | 3.653 | .056 | 1476.369 | .830 | 2625769.584 |
|  | [Palpitations =1] | 0^b^ | . | . | . | . | . | . |
|  | [Chest tightness =0] | .437 | 4.097 | .011 | .915 | 1.548 | .001 | 4757.743 |
|  | [Chest tightness =1] | 0^b^ | . | . | . | . | . | . |
|  | [Shortness of breath =0] | 4.403 | 4.099 | 1.154 | .283 | 81.731 | .026 | 252260.040 |
|  | [Shortness of breath =1] | 0^b^ | . | . | . | . | . | . |
|  | [Chest pain =0] | 23.068 | 13.892 | 2.757 | .097 | 10427661418.361 | .016 | 6969273236461165000000.000 |
|  | [Chest pain =1] | 0^b^ | . | . | . | . | . | . |
|  | [Precordial area =0] | -.386 | 7.726 | .002 | .960 | .680 | 1.804E-7 | 2563478.954 |
|  | [Precordial area =1] | 0^b^ | . | . | . | . | . | . |
|  | [Zhongfu =0] | .492 | 9.555 | .003 | .959 | 1.635 | 1.204E-8 | 222053751.952 |
|  | [Zhongfu =1] | 0^b^ | . | . | . | . | . | . |
|  | [Chest flap =0] | 5.239 | 7.904 | .439 | .507 | 188.562 | 3.526E-5 | 1008510291.110 |
|  | [Chest flap =1] | 0^b^ | . | . | . | . | . | . |
|  | [Pain on the inner side of the shoulder and arm =0] | 34.791 | 8.664 | 16.126 | .000 | 1287380285991861.500 | 54336228.041 | 30501712402949977000000.000 |
|  | [Pain on the inner side of the shoulder and arm =1] | 0^b^ | . | . | . | . | . | . |
|  | [Pain has no fixed location =0] | -5.706 | 14.929 | .146 | .702 | .003 | 6.524E-16 | 16954412688.960 |
|  | [Pain has no fixed location =1] | 0^b^ | . | . | . | . | . | . |
|  | [The pain has a fixed location =0] | 10.590 | 10.975 | .931 | .335 | 39716.473 | 1.808E-5 | 87248271724434.950 |
|  | [The pain has a fixed location =1] | 0^b^ | . | . | . | . | . | . |
|  | [Stabbing pain =0] | 13.155 | 8.078 | 2.652 | .103 | 516359.566 | .069 | 3878826094798.614 |
|  | [Stabbing pain =1] | 0^b^ | . | . | . | . | . | . |
|  | [Dull pain =0] | -9.396 | 8.648 | 1.181 | .277 | 8.306E-5 | 3.618E-12 | 1907.073 |
|  | [Dull pain =1] | 0^b^ | . | . | . | . | . | . |
|  | [Distending pain =0] | -34.215 | 22.510 | 2.310 | .129 | 1.383E-15 | 9.550E-35 | 20020.601 |
|  | [Distending pain =1] | 0^b^ | . | . | . | . | . | . |
|  | [Colic =0] | 4.700 | 10.785 | .190 | .663 | 110.000 | 7.264E-8 | 166569681252.799 |
|  | [Colic =1] | 0^b^ | . | . | . | . | . | . |
|  | [Dull pain =0] | -35.244 | 13.241 | 7.084 | .008 | 4.941E-16 | 2.648E-27 | 9.221E-5 |
|  | [Dull pain =1] | 0^b^ | . | . | . | . | . | . |
|  | [Post-activity increase =0] | -.222 | 5.109 | .002 | .965 | .801 | 3.592E-5 | 17867.003 |
|  | [Post-activity increase =1] | 0^b^ | . | . | . | . | . | . |
|  | [Induced by unfulfilled emotions =0] | -11.246 | 8.275 | 1.847 | .174 | 1.306E-5 | 1.182E-12 | 144.341 |
|  | [Induced by unfulfilled emotions =1] | 0^b^ | . | . | . | . | . | . |
|  | [Induced by drinking and overeating =0]] | -70.375 | 25.965 | 7.346 | .007 | 2.733E-31 | 2.164E-53 | 3.452E-9 |
|  | [Induced by drinking and overeating =1] | 0^b^ | . | . | . | . | . | . |
|  | [Induced by rainy days =0] | 18.157 | 8.381 | 4.694 | .030 | 76820605.876 | 5.644 | 1045567493265029.600 |
|  | [Induced by rainy days =1] | 0^b^ | . | . | . | . | . | . |
|  | [Sudden cold weather or sudden exposure to cold wind triggers =0] | -4.500 | 14.139 | .101 | .750 | .011 | 1.025E-14 | 12036173704.632 |
|  | [Sudden cold weather or sudden exposure to cold wind triggers =1] | 0^b^ | . | . | . | . | . | . |
|  | [Relief after rest =0] | -2.196 | 1.902 | 1.333 | .248 | .111 | .003 | 4.626 |
|  | [Relief after rest =1] | 0^b^ | . | . | . | . | . | . |
|  | [Edema =0] | 3.327 | 7.711 | .186 | .666 | 27.854 | 7.613E-6 | 101910578.431 |
|  | [Edema =1] | 0^b^ | . | . | . | . | . | . |
|  | [Weak and talkative =0] | -4.427 | 3.777 | 1.374 | .241 | .012 | 7.287E-6 | 19.603 |
|  | [Weak and talkative =1] | 0^b^ | . | . | . | . | . | . |
|  | [Feeling annoyed =0] | -9.139 | 5.872 | 2.422 | .120 | .000 | 1.078E-9 | 10.708 |
|  | [Feeling annoyed =1] | 0^b^ | . | . | . | . | . | . |
|  | [Forgetfulness =0] | -8.476 | 5.535 | 2.345 | .126 | .000 | 4.051E-9 | 10.726 |
|  | [Forgetfulness =1] | 0^b^ | . | . | . | . | . | . |
|  | [Heat intolerance =0] | -1.172 | 4.883 | .058 | .810 | .310 | 2.162E-5 | 4435.135 |
|  | [Heat intolerance =1] | 0^b^ | . | . | . | . | . | . |
|  | [Fear of cold =0] | -7.843 | 7.436 | 1.112 | .292 | .000 | 1.838E-10 | 838.573 |
|  | [Fear of cold =1] | 0^b^ | . | . | . | . | . | . |
|  | [Cold limbs =0] | -6.072 | 6.367 | .910 | .340 | .002 | 8.773E-9 | 605.989 |
|  | [Cold limbs =1] | 0^b^ | . | . | . | . | . | . |
|  | [Hot and humid =0] | 1.035 | 9.794 | .011 | .916 | 2.816 | 1.297E-8 | 611456022.541 |
|  | [Hot and humid =1] | 0^b^ | . | . | . | . | . | . |
|  | [Hot palms and soles =0] | -11.564 | 11.412 | 1.027 | .311 | 9.502E-6 | 1.835E-15 | 49204.224 |
|  | [Hot palms and soles =1] | 0^b^ | . | . | . | . | . | . |
|  | [Spontaneous sweating =0] | -10.741 | 4.081 | 6.929 | .008 | 2.163E-5 | 7.273E-9 | .064 |
|  | [Spontaneous sweating =1] | 0^b^ | . | . | . | . | . | . |
|  | [Night sweats =0] | -2.513 | 6.667 | .142 | .706 | .081 | 1.714E-7 | 38293.498 |
|  | [Night sweats =1] | 0^b^ | . | . | . | . | . | . |
|  | [Dizziness =0] | -6.567 | 4.560 | 2.074 | .150 | .001 | 1.848E-7 | 10.692 |
|  | [Dizziness =1] | 0^b^ | . | . | . | . | . | . |
|  | [Dry eyes =0] | -3.865 | 4.265 | .821 | .365 | .021 | 4.906E-6 | 89.600 |
|  | [Dry eyes =1] | 0^b^ | . | . | . | . | . | . |
|  | [Tinnitus =0] | 6.423 | 4.502 | 2.036 | .154 | 615.939 | .091 | 4182291.020 |
|  | [Tinnitus =1] | 0^b^ | . | . | . | . | . | . |
|  | [Mouth ulcers =0] | 7.768 | 5.460 | 2.024 | .155 | 2363.832 | .053 | 104915962.487 |
|  | [Mouth ulcers =1] | 0^b^ | . | . | . | . | . | . |
|  | [Swollen and painful gums =0] | -4.163 | 11.399 | .133 | .715 | .016 | 3.086E-12 | 78434085.690 |
|  | [Swollen and painful gums =1] | 0^b^ | . | . | . | . | . | . |
|  | [Cough =0] | -6.193 | 5.671 | 1.193 | .275 | .002 | 3.042E-8 | 137.287 |
|  | [Cough =1] | 0^b^ | . | . | . | . | . | . |
|  | [White and thin phlegm =0] | -7.201 | 7.978 | .815 | .367 | .001 | 1.206E-10 | 4612.443 |
|  | [White and thin phlegm =1] | 0^b^ | . | . | . | . | . | . |
|  | [White thick phlegm =0] | 8.894 | 10.893 | .667 | .414 | 7284.810 | 3.896E-6 | 13622585241200.027 |
|  | [White thick phlegm =1] | 0^b^ | . | . | . | . | . | . |
|  | [Yellow sticky phlegm =0] | -21.234 | 16.730 | 1.611 | .204 | 6.001E-10 | 3.448E-24 | 104426.027 |
|  | [Yellow sticky phlegm =1] | 0^b^ | . | . | . | . | . | . |
|  | Frothy sputum =0] | 57.304 | 17.904 | 10.243 | .001 | 7703156538441946000000000.000 | 4429385208.859 | 1.340E+40 |
|  | [Frothy sputum =1] | 0^b^ | . | . | . | . | . | . |
|  | [Stomach pain prefers warmth and pressure =0] | -5.298 | 12.406 | .182 | .669 | .005 | 1.377E-13 | 181733371.978 |
|  | [Stomach pain prefers warmth and pressure =1] | 0^b^ | . | . | . | . | . | . |
|  | [Good Sigh =0] | 2.469 | 12.382 | .040 | .842 | 11.808 | 3.410E-10 | 408892522367.795 |
|  | [Good Sigh =1] | 0^b^ | . | . | . | . | . | . |
|  | [Costal distension =0] | -13.675 | 13.094 | 1.091 | .296 | 1.151E-6 | 8.236E-18 | 160758.824 |
|  | [Costal distension =1] | 0^b^ | . | . | . | . | . | . |
|  | [Weakness in the waist and knees =0] | 5.917 | 4.112 | 2.071 | .150 | 371.397 | .117 | 1175008.574 |
|  | [Weakness in the waist and knees =1] | 0^b^ | . | . | . | . | . | . |
|  | [Numbness in hands and feet =0] | 3.696 | 4.403 | .705 | .401 | 40.294 | .007 | 225512.016 |
|  | [Numbness in hands and feet =1] | 0^b^ | . | . | . | . | . | . |
|  | [Body aches all over =0] | -23.269 | 6.067 | 14.709 | .000 | 7.845E-11 | 5.375E-16 | 1.145E-5 |
|  | [Body aches all over =1] | 0^b^ | . | . | . | . | . | . |
|  | [Thirsty and fond of drinking =0] | 4.707 | 4.768 | .975 | .324 | 110.717 | .010 | 1266911.435 |
|  | [Thirsty and fond of drinking =1] | 0^b^ | . | . | . | . | . | . |
|  | [Preference for cold drinks =0] | -29.598 | 11.915 | 6.171 | .013 | 1.399E-13 | 1.009E-23 | .002 |
|  | [Preference for cold drinks =1] | 0^b^ | . | . | . | . | . | . |
|  | [Preference for hot drinks =0] | .796 | 4.682 | .029 | .865 | 2.217 | .000 | 21448.659 |
|  | [Preference for hot drinks =1] | 0^b^ | . | . | . | . | . | . |
|  | [Feeling thirsty but not wanting to drink or drinking very little =0] | -2.897 | 7.156 | .164 | .686 | .055 | 4.470E-8 | 68130.851 |
|  | [Feeling thirsty but not wanting to drink or drinking very little =1] | 0^b^ | . | . | . | . | . | . |
|  | [Poor appetite =0] | .352 | 5.984 | .003 | .953 | 1.422 | 1.147E-5 | 176309.350 |
|  | [Poor appetite =1] | 0^b^ | . | . | . | . | . | . |
|  | [Bitter taste in the mouth =0] | -2.990 | 5.543 | .291 | .590 | .050 | 9.611E-7 | 2628.630 |
|  | [Bitter taste in the mouth =1] | 0^b^ | . | . | . | . | . | . |
|  | [Dry mouth =0] | 4.258 | 5.411 | .619 | .431 | 70.650 | .002 | 2849335.883 |
|  | [Dry mouth =1] | 0^b^ | . | . | . | . | . | . |
|  | [Sweet tooth =0] | .319 | 5.104 | .004 | .950 | 1.375 | 6.222E-5 | 30394.914 |
|  | [Sweet tooth =1] | 0^b^ | . | . | . | . | . | . |
|  | [Preference for salty food =0] | -1.201 | 6.746 | .032 | .859 | .301 | 5.451E-7 | 166221.187 |
|  | [Preference for salty food =1] | 0^b^ | . | . | . | . | . | . |
|  | [Preference for strong tea =0] | -.036 | 4.910 | .000 | .994 | .965 | 6.379E-5 | 14587.962 |
|  | [Preference for strong tea =1] | 0^b^ | . | . | . | . | . | . |
|  | [Love meat =0] | 5.123 | 6.511 | .619 | .431 | 167.770 | .000 | 58423114.050 |
|  | [Love meat =1] | 0^b^ | . | . | . | . | . | . |
|  | [Long-term vegetarianism =0] | 10.573 | 10.486 | 1.017 | .313 | 39050.127 | 4.635E-5 | 32900005139828.844 |
|  | [Long-term vegetarianism =1] | 0^b^ | . | . | . | . | . | . |
|  | [Constipation =0] | -8.511 | 4.351 | 3.826 | .050 | .000 | 3.982E-8 | 1.018 |
|  | [Constipation =1] | 0^b^ | . | . | . | . | . | . |
|  | [Loose stools =0] | -8.410 | 11.729 | .514 | .473 | .000 | 2.311E-14 | 2146931.962 |
|  | [Loose stools =1] | 0^b^ | . | . | . | . | . | . |
|  | [Complete valley Unmelted =0] | -28.043 | 34.527 | .660 | .417 | 6.626E-13 | 2.700E-42 | 162596548047624320.000 |
|  | [Complete valley Unmelted =1] | 0^b^ | . | . | . | . | . | . |
|  | [Morning diarrhea =0] | 30.691 | 141.562 | .047 | .828 | 21328985488146.180 | 6.783E-108 | 6.707E+133 |
|  | [Morning diarrhea =1] | 0^b^ | . | . | . | . | . | . |
|  | [Loose and unregulated knots =0] | -41.523 | 26.468 | 2.461 | .117 | 9.266E-19 | 2.736E-41 | 31379.485 |
|  | [Loose and unregulated knots =1] | 0^b^ | . | . | . | . | . | . |
|  | [Clear and long urine =0] | 36.147 | 126.654 | .081 | .775 | 4994291777508833.000 | 7.773E-93 | 3.209E+123 |
|  | [Clear and long urine =1] | 0^b^ | . | . | . | . | . | . |
|  | [Yellowish urine =0] | 2.376 | 16.097 | .022 | .883 | 10.767 | 2.138E-13 | 542105667972354.800 |
|  | [Yellowish urine =1] | 0^b^ | . | . | . | . | . | . |
|  | [Frequency of urination =0] | 6.588 | 7.387 | .795 | .372 | 726.418 | .000 | 1409207976.577 |
|  | [Frequency of urination =1] | 0^b^ | . | . | . | . | . | . |
|  | [Burning urine =0] | 10.069 | 84.077 | .014 | .905 | 23596.800 | 6.408E-68 | 8.689E+75 |
|  | [Burning urine =1] | 0^b^ | . | . | . | . | . | . |
|  | [Incomplete urination =0] | 1.186 | 8.527 | .019 | .889 | 3.273 | 1.807E-7 | 59284326.125 |
|  | [Incomplete urination =1] | 0^b^ | . | . | . | . | . | . |
|  | [Frequent nocturia =0] | -7.327 | 4.475 | 2.681 | .102 | .001 | 1.022E-7 | 4.236 |
|  | [Frequent nocturia =1] | 0^b^ | . | . | . | . | . | . |
|  | [Insomnia=0] | -4.913 | 5.089 | .932 | .334 | .007 | 3.425E-7 | 157.705 |
|  | [Insomnia=1] | 0^b^ | . | . | . | . | . | . |
|  | [Excessive sleepiness =0] | 1.109 | 13.939 | .006 | .937 | 3.032 | 4.140E-12 | 2220212702632.300 |
|  | [Excessive sleepiness =1] | 0^b^ | . | . | . | . | . | . |
|  | [Irritable and quick-tempered =0] | 2.167 | 4.980 | .189 | .663 | 8.735 | .001 | 151380.396 |
|  | [Irritable and quick-tempered =1] | 0^b^ | . | . | . | . | . | . |
|  | [Easy concern =0] | -12.555 | 11.391 | 1.215 | .270 | 3.527E-6 | 7.096E-16 | 17532.392 |
|  | [Easy concern =1] | 0^b^ | . | . | . | . | . | . |
|  | [Easily nervous =0] | 8.976 | 13.476 | .444 | .505 | 7910.861 | 2.678E-8 | 2337267992312275.500 |
|  | [Easily nervous =1] | 0^b^ | . | . | . | . | . | . |
|  | [Anxiety/Depression=0] | 1.249 | 8.135 | .024 | .878 | 3.489 | 4.154E-7 | 29300896.456 |
|  | [Anxiety/Depression=1] | 0^b^ | . | . | . | . | . | . |
|  | [Prone to catching a cold =0] | -3.922 | 7.071 | .308 | .579 | .020 | 1.897E-8 | 20655.506 |
|  | [Prone to catching a cold =1] | 0^b^ | . | . | . | . | . | . |
|  | [Pale complexion =0] | 12.740 | 419.654 | .001 | .976 | 341139.999 | .000 | .^c^ |
|  | [Pale complexion =1] | 0^b^ | . | . | . | . | . | . |
|  | [Red complexion (cheeks) =0] | 6.685 | 14.095 | .225 | .635 | 800.025 | 8.043E-10 | 795793391050503.500 |
|  | [Red complexion (cheeks) =1] | 0^b^ | . | . | . | . | . | . |
|  | [Pale red complexion =0] | 4.176 | 12.817 | .106 | .745 | 65.111 | 8.010E-10 | 5292586882914.011 |
|  | [Pale red complexion =1] | 0^b^ | . | . | . | . | . | . |
|  | [Dark red complexion =0] | -5.062 | 5.673 | .796 | .372 | .006 | 9.384E-8 | 427.115 |
|  | [Dark red complexion =1] | 0^b^ | . | . | . | . | . | . |
|  | [Redness on both cheeks =0] | 8.786 | 5.796 | 2.298 | .130 | 6542.113 | .076 | 561816147.680 |
|  | [Redness on both cheeks =1] | 0^b^ | . | . | . | . | . | . |
|  | [Pale yellow complexion =0] | -2.186 | 4.576 | .228 | .633 | .112 | 1.431E-5 | 882.883 |
|  | [Pale yellow complexion =1] | 0^b^ | . | . | . | . | . | . |
|  | [Yellow complexion =0] | 6.483 | 4.283 | 2.291 | .130 | 653.641 | .148 | 2890079.123 |
|  | [Yellow complexion =1] | 0^b^ | . | . | . | . | . | . |
|  | [Sallow complexion =0] | .979 | 6.415 | .023 | .879 | 2.661 | 9.213E-6 | 768420.294 |
|  | [Sallow complexion =1] | 0^b^ | . | . | . | . | . | . |
|  | [Sallow complexion =0] | 15.769 | 8.129 | 3.763 | .052 | 7053531.850 | .850 | 58566534817325.100 |
|  | [Sallow complexion =1] | 0^b^ | . | . | . | . | . | . |
|  | [Pale complexion =0] | 8.864 | 7.149 | 1.537 | .215 | 7071.349 | .006 | 8604352197.632 |
|  | [Pale complexion =1] | 0^b^ | . | . | . | . | . | . |
|  | [Dark complexion =0] | 23.813 | 26.853 | .786 | .375 | 21981294502.667 | 3.052E-13 | 1583388536805660500000000000000000.000 |
|  | [Dark complexion =1] | 0^b^ | . | . | . | . | . | . |
|  | [Sallow complexion =0] | 39.053 | 55.280 | .499 | .480 | 91278819980308736.000 | 8.059E-31 | 1.034E+64 |
|  | [Sallow complexion =1] | 0^b^ | . | . | . | . | . | . |
|  | [Radiant complexion =0] | -6.974 | 6.447 | 1.170 | .279 | .001 | 3.046E-9 | 287.736 |
|  | [Radiant complexion =1] | 0^b^ | . | . | . | . | . | . |
|  | [Pale complexion =0] | -8.505 | 6.028 | 1.991 | .158 | .000 | 1.497E-9 | 27.373 |
|  | [Pale complexion =1] | 0^b^ | . | . | . | . | . | . |
|  | [Pale complexion =0] | 19.227 | 1319.462 | .000 | .988 | 224066058.927 | .000 | .^c^ |
|  | [Pale complexion =1] | 0^b^ | . | . | . | . | . | . |
|  | [Dark eye orbit color =0] | -2.522 | 5.131 | .242 | .623 | .080 | 3.445E-6 | 1871.024 |
|  | [Dark eye orbit color =1] | 0^b^ | . | . | . | . | . | . |
|  | [Pale lip nail =0] | -7.882 | 14.377 | .301 | .584 | .000 | 2.183E-16 | 652809902.066 |
|  | [Pale lip nail =1] | 0^b^ | . | . | . | . | . | . |
|  | [Lip nail red =0] | -7.996 | 9.103 | .772 | .380 | .000 | 6.012E-12 | 18874.278 |
|  | [Lip nail red =1] | 0^b^ | . | . | . | . | . | . |
|  | [Light red lip nail =0] | -8.974 | 7.052 | 1.619 | .203 | .000 | 1.258E-10 | 127.497 |
|  | [Light red lip nail =1] | 0^b^ | . | . | . | . | . | . |
|  | [Dark red lip nail =0] | -6.607 | 5.842 | 1.279 | .258 | .001 | 1.438E-8 | 126.892 |
|  | [Dark red lip nail =1] | 0^b^ | . | . | . | . | . | . |
|  | [Dark purple lip nail =0] | -8.466 | 7.551 | 1.257 | .262 | .000 | 7.863E-11 | 562.982 |
|  | [Dark purple lip nail =1] | 0^b^ | . | . | . | . | . | . |
|  | [Lip and nail moisture =0] | 20.223 | 37.824 | .286 | .593 | 606292354.017 | 3.864E-24 | 9.514E+40 |
|  | [Lip and nail moisture =1] | 0^b^ | . | . | . | . | . | . |
|  | [Dry lips and nails =0] | 3.880 | 2.991 | 1.682 | .195 | 48.420 | .138 | 17035.370 |
|  | [Dry lips and nails =1] | 0^b^ | . | . | . | . | . | . |
|  | [Pale red tongue =0] | -5.935 | 9.906 | .359 | .549 | .003 | 9.778E-12 | 715126.149 |
|  | [Pale red tongue =1] | 0^b^ | . | . | . | . | . | . |
|  | [Pale tongue =0] | 6.567 | 28.519 | .053 | .818 | 711.406 | 3.774E-22 | 1340944384821052700000000000.000 |
|  | [Pale tongue =1] | 0^b^ | . | . | . | . | . | . |
|  | [Red tongue =0] | -17.487 | 9.737 | 3.225 | .073 | 2.544E-8 | 1.310E-16 | 4.940 |
|  | [Red tongue =1] | 0^b^ | . | . | . | . | . | . |
|  | [Purple Dark Tongue=0] | -8.560 | 9.539 | .805 | .369 | .000 | 1.455E-12 | 25211.904 |
|  | [Purple Dark Tongue=1] | 0^b^ | . | . | . | . | . | . |
|  | [Crimson tongue =0] | -20.247 | 11.195 | 3.271 | .071 | 1.610E-9 | 4.757E-19 | 5.449 |
|  | [Crimson tongue =1] | 0^b^ | . | . | . | . | . | . |
|  | [Red tongue tip =0] | 8.491 | 7.058 | 1.447 | .229 | 4869.525 | .005 | 4960128276.666 |
|  | [Red tongue tip =1] | 0^b^ | . | . | . | . | . | . |
|  | [Fat tongue =0] | -27.476 | 9.767 | 7.914 | .005 | 1.167E-12 | 5.667E-21 | .000 |
|  | [Fat tongue =1] | 0^b^ | . | . | . | . | . | . |
|  | [Tooth mark tongue =0] | .327 | 2.738 | .014 | .905 | 1.387 | .006 | 296.683 |
|  | [Tooth mark tongue =1] | 0^b^ | . | . | . | . | . | . |
|  | [Old tongue =0] | -.922 | 2.802 | .108 | .742 | .398 | .002 | 96.547 |
|  | [Old tongue =1] | 0^b^ | . | . | . | . | . | . |
|  | [Tender tongue =0] | -5.680 | 5.988 | .900 | .343 | .003 | 2.729E-8 | 427.194 |
|  | [Tender tongue =1] | 0^b^ | . | . | . | . | . | . |
|  | [Cracked tongue =0] | 4.063 | 3.010 | 1.822 | .177 | 58.162 | .159 | 21240.797 |
|  | [Cracked tongue =1] | 0^b^ | . | . | . | . | . | . |
|  | [Ecchymosis tongue =0] | .468 | 4.924 | .009 | .924 | 1.597 | .000 | 24809.790 |
|  | [Ecchymosis tongue =1] | 0^b^ | . | . | . | . | . | . |
|  | [Point the tongue =0] | -7.779 | 4.844 | 2.580 | .108 | .000 | 3.153E-8 | 5.551 |
|  | [Point the tongue =1] | 0^b^ | . | . | . | . | . | . |
|  | [Thin moss =0] | 5.897 | 8.824 | .447 | .504 | 363.948 | 1.122E-5 | 11809860156.768 |
|  | [Thin moss =1] | 0^b^ | . | . | . | . | . | . |
|  | [Moss thickness =0] | -.081 | 8.289 | .000 | .992 | .922 | 8.117E-8 | 10479401.688 |
|  | [Moss thickness =1] | 0^b^ | . | . | . | . | . | . |
|  | [Tai Ni =0] | -1.633 | 6.198 | .069 | .792 | .195 | 1.034E-6 | 36876.170 |
|  | [Tai Ni =1] | 0^b^ | . | . | . | . | . | . |
|  | [Moss decay =0] | 2.458 | 14.105 | .030 | .862 | 11.676 | 1.151E-11 | 11847011385008.865 |
|  | [Moss decay =1] | 0^b^ | . | . | . | . | . | . |
|  | [Tairun =0] | 10.623 | 9.603 | 1.224 | .269 | 41059.767 | .000 | 6136066045260.483 |
|  | [Tairun =1] | 0^b^ | . | . | . | . | . | . |
|  | [Taizao =0] | -.654 | 9.252 | .005 | .944 | .520 | 6.929E-9 | 39030302.163 |
|  | [Taizao =1] | 0^b^ | . | . | . | . | . | . |
|  | [Slippery tongue =0] | 30.236 | 46.732 | .419 | .518 | 13534365438638.082 | 2.254E-27 | 8.128E+52 |
|  | [Slippery tongue =1] | 0^b^ | . | . | . | . | . | . |
|  | [Less moss =0] | -12.693 | 8.295 | 2.341 | .126 | 3.072E-6 | 2.669E-13 | 35.351 |
|  | [Less moss =1] | 0^b^ | . | . | . | . | . | . |
|  | [Mirror tongue =0] | 48.979 | 28.880 | 2.876 | .090 | 1867126206547483000000.000 | .000 | 7.138E+45 |
|  | [Mirror tongue =1] | 0^b^ | . | . | . | . | . | . |
|  | [Full tongue coating =0] | -2.833 | 3.482 | .662 | .416 | .059 | 6.394E-5 | 54.156 |
|  | [Full tongue coating =1] | 0^b^ | . | . | . | . | . | . |
|  | [Moss peeling =0] | -20.459 | 4.474 | 20.912 | .000 | 1.302E-9 | 2.026E-13 | 8.374E-6 |
|  | [Moss peeling =1] | 0^b^ | . | . | . | . | . | . |
|  | [White coating on the tongue =0] | 19.979 | 31.052 | .414 | .520 | 474888590.955 | 1.760E-18 | 128142577603007200000000000000000000.000 |
|  | [White coating on the tongue =1] | 0^b^ | . | . | . | . | . | . |
|  | [Yellow coating on the tongue =0] | 21.723 | 31.214 | .484 | .486 | 2718298564.891 | 7.333E-18 | 1.008E+36 |
|  | [Yellow coating on the tongue =1] | 0^b^ | . | . | . | . | . | . |
|  | [Yellow and white coating on the tongue =0] | 21.769 | 31.403 | .481 | .488 | 2844165105.164 | 5.289E-18 | 1.529E+36 |
|  | [Yellow and white coating on the tongue =1] | 0^b^ | . | . | . | . | . | . |
|  | [Grayish-black coating on the tongue =0] | 27.588 | 22.273 | 1.534 | .215 | 957592070964.331 | 1.053E-7 | 8712423375981336000000000000000.000 |
|  | [Grayish-black coating on the tongue =1] | 0^b^ | . | . | . | . | . | . |
|  | [pulse float =0] | -5.640 | 5.090 | 1.228 | .268 | .004 | 1.652E-7 | 76.364 |
|  | [pulse float =1] | 0^b^ | . | . | . | . | . | . |
|  | [pulse depth =0] | -5.992 | 4.222 | 2.014 | .156 | .002 | 6.369E-7 | 9.802 |
|  | [pulse depth =1] | 0^b^ | . | . | . | . | . | . |
|  | [Pulse slow =0] | -10.549 | 10.348 | 1.039 | .308 | 2.622E-5 | 4.074E-14 | 16879.294 |
|  | [Pulse slow =1] | 0^b^ | . | . | . | . | . | . |
|  | [Pulse fat =0] | -4.302 | 3.743 | 1.321 | .250 | .014 | 8.825E-6 | 20.791 |
|  | [Pulse fat =1] | 0^b^ | . | . | . | . | . | . |
|  | [ Pulse flood =0] | 52.162 | 469.687 | .012 | .912 | 45066084268789740000000.000 | .000 | .^c^ |
|  | [Pulse flood =1] | 0^b^ | . | . | . | . | . | . |
|  | [ pulse fineness =0] | -2.845 | 5.382 | .279 | .597 | .058 | 1.526E-6 | 2217.287 |
|  | [ pulse fineness =1] | 0^b^ | . | . | . | . | . | . |
|  | [Weak pulse=0] | -6.192 | 9.597 | .416 | .519 | .002 | 1.385E-11 | 301955.113 |
|  | [Weak pulse=1] | 0^b^ | . | . | . | . | . | . |
|  | [pulse slippery =0] | -1.707 | 3.810 | .201 | .654 | .181 | .000 | 317.518 |
|  | [pulse slippery =1] | 0^b^ | . | . | . | . | . | . |
|  | [pulse astringency =0] | 10.205 | 8.699 | 1.376 | .241 | 27043.251 | .001 | 685942122701.666 |
|  | [pulse astringency =1] | 0^b^ | . | . | . | . | . | . |
|  | [pulse string =0] | -4.071 | 3.931 | 1.073 | .300 | .017 | 7.687E-6 | 37.838 |
|  | [pulse string =1] | 0^b^ | . | . | . | . | . | . |
|  | [pulse tightness =0] | 9.250 | 22.215 | .173 | .677 | 10403.953 | 1.282E-15 | 84402941501037460000000.000 |
|  | [pulse tightness =1] | 0^b^ | . | . | . | . | . | . |
|  | [pulse knot representation =0] | 2.222 | 6.241 | .127 | .722 | 9.225 | 4.492E-5 | 1894580.565 |
|  | [pulse knot representation =1] | 0^b^ | . | . | . | . | . | . |
| Syndrome of Yin deficiency of heart and kidney | Intercept | -113.419 | 1475.555 | .006 | .939 |  |  |  |
|  | [Palpitations =0] | 4.965 | 3.879 | 1.639 | .200 | 143.374 | .072 | 287077.485 |
|  | [Palpitations =1] | 0^b^ | . | . | . | . | . | . |
|  | [Chest tightness =0] | 3.783 | 4.136 | .837 | .360 | 43.931 | .013 | 145533.268 |
|  | [Chest tightness =1] | 0^b^ | . | . | . | . | . | . |
|  | [Shortness of breath =0] | 6.134 | 4.148 | 2.187 | .139 | 461.240 | .136 | 1565577.343 |
|  | [Shortness of breath =1] | 0^b^ | . | . | . | . | . | . |
|  | [Chest pain =0] | 28.419 | 13.615 | 4.357 | .037 | 2198984129002.990 | 5.661 | 854153885890076700000000.000 |
|  | [Chest pain =1] | 0^b^ | . | . | . | . | . | . |
|  | [Precordial area =0] | 4.057 | 7.473 | .295 | .587 | 57.786 | 2.515E-5 | 132771148.648 |
|  | [Precordial area =1] | 0^b^ | . | . | . | . | . | . |
|  | [Zhongfu =0] | 8.287 | 9.512 | .759 | .384 | 3971.204 | 3.178E-5 | 496169271737.826 |
|  | [Zhongfu =1] | 0^b^ | . | . | . | . | . | . |
|  | [Chest flap =0] | 13.469 | 7.853 | 2.941 | .086 | 707046.627 | .146 | 3422592096758.916 |
|  | [Chest flap =1] | 0^b^ | . | . | . | . | . | . |
|  | [Pain on the inner side of the shoulder and arm =0] | 35.981 | 8.739 | 16.953 | .000 | 4229127416781325.000 | 154152590.257 | 116024769208078380000000.000 |
|  | [Pain on the inner side of the shoulder and arm =1] | 0^b^ | . | . | . | . | . | . |
|  | [Pain has no fixed location =0] | -6.598 | 15.119 | .190 | .663 | .001 | 1.842E-16 | 10098643397.375 |
|  | [Pain has no fixed location =1] | 0^b^ | . | . | . | . | . | . |
|  | [The pain has a fixed location =0] | 2.450 | 10.917 | .050 | .822 | 11.594 | 5.914E-9 | 22727138230.146 |
|  | [The pain has a fixed location =1] | 0^b^ | . | . | . | . | . | . |
|  | [Stabbing pain =0] | 10.752 | 8.024 | 1.796 | .180 | 46724.890 | .007 | 315895371851.332 |
|  | [Stabbing pain =1] | 0^b^ | . | . | . | . | . | . |
|  | [Dull pain =0] | -9.774 | 8.640 | 1.280 | .258 | 5.690E-5 | 2.517E-12 | 1286.282 |
|  | [Dull pain =1] | 0^b^ | . | . | . | . | . | . |
|  | [Distending pain =0] | -34.292 | 22.469 | 2.329 | .127 | 1.280E-15 | 9.587E-35 | 17084.932 |
|  | [Distending pain =1] | 0^b^ | . | . | . | . | . | . |
|  | [Colic =0] | 11.446 | 16.101 | .505 | .477 | 93560.538 | 1.843E-9 | 4749307527961694200.000 |
|  | [Colic =1] | 0^b^ | . | . | . | . | . | . |
|  | [Dull pain =0] | -22.720 | 13.171 | 2.976 | .085 | 1.358E-10 | 8.355E-22 | 22.060 |
|  | [Dull pain =1] | 0^b^ | . | . | . | . | . | . |
|  | [Post-activity increase =0] | -.078 | 5.130 | .000 | .988 | .925 | 3.976E-5 | 21521.034 |
|  | [Post-activity increase =1] | 0^b^ | . | . | . | . | . | . |
|  | [Induced by unfulfilled emotions =0] | -6.595 | 8.261 | .637 | .425 | .001 | 1.270E-10 | 14706.985 |
|  | [Induced by unfulfilled emotions =1] | 0^b^ | . | . | . | . | . | . |
|  | [Induced by drinking and overeating =0]] | -68.857 | 26.062 | 6.980 | .008 | 1.247E-30 | 8.163E-53 | 1.905E-8 |
|  | [Induced by drinking and overeating =1] | 0^b^ | . | . | . | . | . | . |
|  | [Induced by rainy days =0] | 18.482 | 8.358 | 4.890 | .027 | 106367872.387 | 8.174 | 1384084439980959.000 |
|  | [Induced by rainy days =1] | 0^b^ | . | . | . | . | . | . |
|  | [Sudden cold weather or sudden exposure to cold wind triggers =0] | -6.212 | 14.142 | .193 | .660 | .002 | 1.838E-15 | 2187458571.313 |
|  | [Sudden cold weather or sudden exposure to cold wind triggers =1] | 0^b^ | . | . | . | . | . | . |
|  | [Relief after rest =0] | 1.220 | 1.855 | .433 | .511 | 3.388 | .089 | 128.416 |
|  | [Relief after rest =1] | 0^b^ | . | . | . | . | . | . |
|  | [Edema =0] | 5.354 | 7.761 | .476 | .490 | 211.518 | 5.239E-5 | 853974014.309 |
|  | [Edema =1] | 0^b^ | . | . | . | . | . | . |
|  | [Weak and talkative =0] | -2.195 | 3.746 | .343 | .558 | .111 | 7.216E-5 | 171.990 |
|  | [Weak and talkative =1] | 0^b^ | . | . | . | . | . | . |
|  | [Feeling annoyed =0] | -6.545 | 5.813 | 1.268 | .260 | .001 | 1.620E-8 | 127.483 |
|  | [Feeling annoyed =1] | 0^b^ | . | . | . | . | . | . |
|  | [Forgetfulness =0] | -11.571 | 5.576 | 4.306 | .038 | 9.437E-6 | 1.692E-10 | .526 |
|  | [Forgetfulness =1] | 0^b^ | . | . | . | . | . | . |
|  | [Heat intolerance =0] | -2.800 | 4.918 | .324 | .569 | .061 | 3.959E-6 | 933.126 |
|  | [Heat intolerance =1] | 0^b^ | . | . | . | . | . | . |
|  | [Fear of cold =0] | -8.736 | 7.467 | 1.369 | .242 | .000 | 7.082E-11 | 364.262 |
|  | [Fear of cold =1] | 0^b^ | . | . | . | . | . | . |
|  | [Cold limbs =0] | -5.120 | 6.385 | .643 | .423 | .006 | 2.194E-8 | 1629.016 |
|  | [Cold limbs =1] | 0^b^ | . | . | . | . | . | . |
|  | [Hot and humid =0] | -5.171 | 9.733 | .282 | .595 | .006 | 2.946E-11 | 1094752.316 |
|  | [Hot and humid =1] | 0^b^ | . | . | . | . | . | . |
|  | [Hot palms and soles =0] | -20.840 | 11.466 | 3.303 | .069 | 8.902E-10 | 1.546E-19 | 5.126 |
|  | [Hot palms and soles =1] | 0^b^ | . | . | . | . | . | . |
|  | [Spontaneous sweating =0] | -10.163 | 4.067 | 6.247 | .012 | 3.855E-5 | 1.332E-8 | .112 |
|  | [Spontaneous sweating =1] | 0^b^ | . | . | . | . | . | . |
|  | [Night sweats =0] | -5.807 | 6.633 | .766 | .381 | .003 | 6.798E-9 | 1330.336 |
|  | [Night sweats =1] | 0^b^ | . | . | . | . | . | . |
|  | [Dizziness =0] | -5.744 | 4.590 | 1.566 | .211 | .003 | 3.961E-7 | 25.861 |
|  | [Dizziness =1] | 0^b^ | . | . | . | . | . | . |
|  | [Dry eyes =0] | -12.472 | 4.260 | 8.571 | .003 | 3.831E-6 | 9.057E-10 | .016 |
|  | [Dry eyes =1] | 0^b^ | . | . | . | . | . | . |
|  | [Tinnitus =0] | 6.447 | 4.493 | 2.059 | .151 | 630.889 | .094 | 4213087.915 |
|  | [Tinnitus =1] | 0^b^ | . | . | . | . | . | . |
|  | [Mouth ulcers =0] | 5.111 | 5.420 | .889 | .346 | 165.883 | .004 | 6813252.241 |
|  | [Mouth ulcers =1] | 0^b^ | . | . | . | . | . | . |
|  | [Swollen and painful gums =0] | -1.545 | 11.392 | .018 | .892 | .213 | 4.283E-11 | 1062631562.695 |
|  | [Swollen and painful gums =1] | 0^b^ | . | . | . | . | . | . |
|  | [Cough =0] | -10.789 | 5.612 | 3.695 | .055 | 2.063E-5 | 3.445E-10 | 1.236 |
|  | [Cough =1] | 0^b^ | . | . | . | . | . | . |
|  | [White and thin phlegm =0] | -5.097 | 7.952 | .411 | .521 | .006 | 1.042E-9 | 35866.530 |
|  | [White and thin phlegm =1] | 0^b^ | . | . | . | . | . | . |
|  | [White thick phlegm =0] | 9.703 | 10.754 | .814 | .367 | 16366.398 | 1.148E-5 | 23328980337313.960 |
|  | [White thick phlegm =1] | 0^b^ | . | . | . | . | . | . |
|  | [Yellow sticky phlegm =0] | -17.417 | 16.511 | 1.113 | .291 | 2.728E-8 | 2.406E-22 | 3093481.839 |
|  | [Yellow sticky phlegm =1] | 0^b^ | . | . | . | . | . | . |
|  | Frothy sputum =0] | 47.316 | 17.790 | 7.074 | .008 | 354061206055023400000.000 | 254591.547 | 492393950381034000000000000000000000.000 |
|  | [Frothy sputum =1] | 0^b^ | . | . | . | . | . | . |
|  | [Stomach pain prefers warmth and pressure =0] | -12.897 | 12.336 | 1.093 | .296 | 2.504E-6 | 7.908E-17 | 79319.053 |
|  | [Stomach pain prefers warmth and pressure =1] | 0^b^ | . | . | . | . | . | . |
|  | [Good Sigh =0] | 4.071 | 12.339 | .109 | .741 | 58.635 | 1.842E-9 | 1866143590136.501 |
|  | [Good Sigh =1] | 0^b^ | . | . | . | . | . | . |
|  | [Costal distension =0] | -15.628 | 12.915 | 1.464 | .226 | 1.633E-7 | 1.658E-18 | 16075.881 |
|  | [Costal distension =1] | 0^b^ | . | . | . | . | . | . |
|  | [Weakness in the waist and knees =0] | 1.728 | 4.087 | .179 | .672 | 5.631 | .002 | 16964.088 |
|  | [Weakness in the waist and knees =1] | 0^b^ | . | . | . | . | . | . |
|  | [Numbness in hands and feet =0] | 3.304 | 4.424 | .558 | .455 | 27.215 | .005 | 158743.238 |
|  | [Numbness in hands and feet =1] | 0^b^ | . | . | . | . | . | . |
|  | [Body aches all over =0] | -12.108 | 6.044 | 4.013 | .045 | 5.517E-6 | 3.952E-11 | .770 |
|  | [Body aches all over =1] | 0^b^ | . | . | . | . | . | . |
|  | [Thirsty and fond of drinking =0] | 3.309 | 4.668 | .502 | .478 | 27.353 | .003 | 257268.881 |
|  | [Thirsty and fond of drinking =1] | 0^b^ | . | . | . | . | . | . |
|  | [Preference for cold drinks =0] | -25.478 | 11.913 | 4.574 | .032 | 8.608E-12 | 6.228E-22 | .119 |
|  | [Preference for cold drinks =1] | 0^b^ | . | . | . | . | . | . |
|  | [Preference for hot drinks =0] | 3.095 | 4.618 | .449 | .503 | 22.083 | .003 | 188394.890 |
|  | [Preference for hot drinks =1] | 0^b^ | . | . | . | . | . | . |
|  | [Feeling thirsty but not wanting to drink or drinking very little =0] | .281 | 7.134 | .002 | .969 | 1.325 | 1.121E-6 | 1565740.942 |
|  | [Feeling thirsty but not wanting to drink or drinking very little =1] | 0^b^ | . | . | . | . | . | . |
|  | [Poor appetite =0] | -.425 | 5.990 | .005 | .943 | .654 | 5.213E-6 | 82046.379 |
|  | [Poor appetite =1] | 0^b^ | . | . | . | . | . | . |
|  | [Bitter taste in the mouth =0] | -1.772 | 5.505 | .104 | .748 | .170 | 3.507E-6 | 8247.325 |
|  | [Bitter taste in the mouth =1] | 0^b^ | . | . | . | . | . | . |
|  | [Dry mouth =0] | 3.565 | 5.432 | .431 | .512 | 35.324 | .001 | 1486333.195 |
|  | [Dry mouth =1] | 0^b^ | . | . | . | . | . | . |
|  | [Sweet tooth =0] | -1.124 | 5.105 | .049 | .826 | .325 | 1.466E-5 | 7195.138 |
|  | [Sweet tooth =1] | 0^b^ | . | . | . | . | . | . |
|  | [Preference for salty food =0] | -.460 | 6.749 | .005 | .946 | .631 | 1.137E-6 | 350273.592 |
|  | [Preference for salty food =1] | 0^b^ | . | . | . | . | . | . |
|  | [Preference for strong tea =0] | -2.402 | 4.939 | .237 | .627 | .090 | 5.655E-6 | 1448.151 |
|  | [Preference for strong tea =1] | 0^b^ | . | . | . | . | . | . |
|  | [Love meat =0] | 8.807 | 6.568 | 1.798 | .180 | 6680.193 | .017 | 2604034417.200 |
|  | [Love meat =1] | 0^b^ | . | . | . | . | . | . |
|  | [Long-term vegetarianism =0] | 9.731 | 10.434 | .870 | .351 | 16839.478 | 2.211E-5 | 12827654347127.436 |
|  | [Long-term vegetarianism =1] | 0^b^ | . | . | . | . | . | . |
|  | [Constipation =0] | -10.468 | 4.333 | 5.838 | .016 | 2.842E-5 | 5.832E-9 | .139 |
|  | [Constipation =1] | 0^b^ | . | . | . | . | . | . |
|  | [Loose stools =0] | -4.478 | 11.654 | .148 | .701 | .011 | 1.366E-12 | 94479742.813 |
|  | [Loose stools =1] | 0^b^ | . | . | . | . | . | . |
|  | [Complete valley Unmelted =0] | -26.059 | 34.997 | .554 | .457 | 4.817E-12 | 7.814E-42 | 2969106753955105300.000 |
|  | [Complete valley Unmelted =1] | 0^b^ | . | . | . | . | . | . |
|  | [Morning diarrhea =0] | 25.964 | 142.073 | .033 | .855 | 188857072619.872 | 2.204E-110 | 1.619E+132 |
|  | [Morning diarrhea =1] | 0^b^ | . | . | . | . | . | . |
|  | [Loose and unregulated knots =0] | -42.955 | 26.544 | 2.619 | .106 | 2.213E-19 | 5.628E-42 | 8699.934 |
|  | [Loose and unregulated knots =1] | 0^b^ | . | . | . | . | . | . |
|  | [Clear and long urine =0] | 30.863 | 159.946 | .037 | .847 | 25334307956834.645 | 1.810E-123 | 3.546E+149 |
|  | [Clear and long urine =1] | 0^b^ | . | . | . | . | . | . |
|  | [Yellowish urine =0] | .964 | 15.411 | .004 | .950 | 2.623 | 2.001E-13 | 34383802548535.082 |
|  | [Yellowish urine =1] | 0^b^ | . | . | . | . | . | . |
|  | [Frequency of urination =0] | 6.567 | 7.399 | .788 | .375 | 711.328 | .000 | 1412631254.719 |
|  | [Frequency of urination =1] | 0^b^ | . | . | . | . | . | . |
|  | [Burning urine =0] | 5.485 | 93.377 | .003 | .953 | 241.147 | 7.939E-78 | 7.325E+81 |
|  | [Burning urine =1] | 0^b^ | . | . | . | . | . | . |
|  | [Incomplete urination =0] | 10.852 | 8.548 | 1.612 | .204 | 51661.176 | .003 | 976325496912.255 |
|  | [Incomplete urination =1] | 0^b^ | . | . | . | . | . | . |
|  | [Frequent nocturia =0] | -8.429 | 4.445 | 3.596 | .058 | .000 | 3.597E-8 | 1.327 |
|  | [Frequent nocturia =1] | 0^b^ | . | . | . | . | . | . |
|  | [Insomnia=0] | -12.708 | 5.139 | 6.114 | .013 | 3.026E-6 | 1.277E-10 | .072 |
|  | [Insomnia=1] | 0^b^ | . | . | . | . | . | . |
|  | [Excessive sleepiness =0] | 4.878 | 13.989 | .122 | .727 | 131.420 | 1.626E-10 | 106220887391312.170 |
|  | [Excessive sleepiness =1] | 0^b^ | . | . | . | . | . | . |
|  | [Irritable and quick-tempered =0] | 3.255 | 4.958 | .431 | .512 | 25.907 | .002 | 430344.391 |
|  | [Irritable and quick-tempered =1] | 0^b^ | . | . | . | . | . | . |
|  | [Easy concern =0] | -9.783 | 11.386 | .738 | .390 | 5.641E-5 | 1.146E-14 | 277536.711 |
|  | [Easy concern =1] | 0^b^ | . | . | . | . | . | . |
|  | [Easily nervous =0] | 8.062 | 13.464 | .359 | .549 | 3171.326 | 1.099E-8 | 915372050419829.900 |
|  | [Easily nervous =1] | 0^b^ | . | . | . | . | . | . |
|  | [Anxiety/Depression=0] | -1.285 | 8.108 | .025 | .874 | .277 | 3.471E-8 | 2206915.111 |
|  | [Anxiety/Depression=1] | 0^b^ | . | . | . | . | . | . |
|  | [Prone to catching a cold =0] | -1.090 | 7.076 | .024 | .878 | .336 | 3.186E-7 | 354564.801 |
|  | [Prone to catching a cold =1] | 0^b^ | . | . | . | . | . | . |
|  | [Pale complexion =0] | -13.887 | 455.579 | .001 | .976 | 9.307E-7 | .000 | .^c^ |
|  | [Pale complexion =1] | 0^b^ | . | . | . | . | . | . |
|  | [Red complexion (cheeks) =0] | 3.756 | 14.154 | .070 | .791 | 42.779 | 3.831E-11 | 47774390466140.305 |
|  | [Red complexion (cheeks) =1] | 0^b^ | . | . | . | . | . | . |
|  | [Pale red complexion =0] | -1.347 | 12.829 | .011 | .916 | .260 | 3.126E-12 | 21629034406.174 |
|  | [Pale red complexion =1] | 0^b^ | . | . | . | . | . | . |
|  | [Dark red complexion =0] | -3.284 | 5.544 | .351 | .554 | .037 | 7.148E-7 | 1963.098 |
|  | [Dark red complexion =1] | 0^b^ | . | . | . | . | . | . |
|  | [Redness on both cheeks =0] | 7.569 | 5.782 | 1.714 | .190 | 1937.251 | .023 | 161601845.841 |
|  | [Redness on both cheeks =1] | 0^b^ | . | . | . | . | . | . |
|  | [Pale yellow complexion =0] | -1.876 | 4.588 | .167 | .683 | .153 | 1.906E-5 | 1232.389 |
|  | [Pale yellow complexion =1] | 0^b^ | . | . | . | . | . | . |
|  | [Yellow complexion =0] | 7.612 | 4.341 | 3.074 | .080 | 2022.856 | .408 | 10033227.738 |
|  | [Yellow complexion =1] | 0^b^ | . | . | . | . | . | . |
|  | [Sallow complexion =0] | .760 | 6.392 | .014 | .905 | 2.139 | 7.751E-6 | 590192.188 |
|  | [Sallow complexion =1] | 0^b^ | . | . | . | . | . | . |
|  | [Sallow complexion =0] | 14.721 | 8.126 | 3.282 | .070 | 2473922.302 | .299 | 20437189102691.637 |
|  | [Sallow complexion =1] | 0^b^ | . | . | . | . | . | . |
|  | [Pale complexion =0] | 6.301 | 7.068 | .795 | .373 | 545.056 | .001 | 566071592.222 |
|  | [Pale complexion =1] | 0^b^ | . | . | . | . | . | . |
|  | [Dark complexion =0] | 19.769 | 10.525 | 3.528 | .060 | 384939408.153 | .423 | 349958806260843200.000 |
|  | [Dark complexion =1] | 0^b^ | . | . | . | . | . | . |
|  | [Sallow complexion =0] | 34.310 | 45.011 | .581 | .446 | 795725629402655.600 | 3.864E-24 | 1.639E+53 |
|  | [Sallow complexion =1] | 0^b^ | . | . | . | . | . | . |
|  | [Radiant complexion =0] | -8.181 | 6.281 | 1.697 | .193 | .000 | 1.262E-9 | 62.083 |
|  | [Radiant complexion =1] | 0^b^ | . | . | . | . | . | . |
|  | [Pale complexion =0] | -7.246 | 5.845 | 1.537 | .215 | .001 | 7.554E-9 | 67.363 |
|  | [Pale complexion =1] | 0^b^ | . | . | . | . | . | . |
|  | [Pale complexion =0] | 23.007 | 1356.653 | .000 | .986 | 9810978498.441 | .000 | .^c^ |
|  | [Pale complexion =1] | 0^b^ | . | . | . | . | . | . |
|  | [Dark eye orbit color =0] | -3.831 | 5.142 | .555 | .456 | .022 | 9.112E-7 | 516.033 |
|  | [Dark eye orbit color =1] | 0^b^ | . | . | . | . | . | . |
|  | [Pale lip nail =0] | .514 | 14.422 | .001 | .972 | 1.672 | 8.849E-13 | 3160512886318.930 |
|  | [Pale lip nail =1] | 0^b^ | . | . | . | . | . | . |
|  | [Lip nail red =0] | -7.095 | 9.244 | .589 | .443 | .001 | 1.123E-11 | 61293.267 |
|  | [Lip nail red =1] | 0^b^ | . | . | . | . | . | . |
|  | [Light red lip nail =0] | -5.835 | 7.279 | .643 | .423 | .003 | 1.862E-9 | 4585.931 |
|  | [Light red lip nail =1] | 0^b^ | . | . | . | . | . | . |
|  | [Dark red lip nail =0] | -4.454 | 6.044 | .543 | .461 | .012 | 8.326E-8 | 1623.894 |
|  | [Dark red lip nail =1] | 0^b^ | . | . | . | . | . | . |
|  | [Dark purple lip nail =0] | -7.991 | 7.735 | 1.067 | .302 | .000 | 8.815E-11 | 1299.802 |
|  | [Dark purple lip nail =1] | 0^b^ | . | . | . | . | . | . |
|  | [Lip and nail moisture =0] | 33.666 | 38.055 | .783 | .376 | 417682432616029.500 | 1.693E-18 | 1.031E+47 |
|  | [Lip and nail moisture =1] | 0^b^ | . | . | . | . | . | . |
|  | [Dry lips and nails =0] | 1.952 | 2.712 | .518 | .472 | 7.044 | .035 | 1432.873 |
|  | [Dry lips and nails =1] | 0^b^ | . | . | . | . | . | . |
|  | [Pale red tongue =0] | -5.342 | 10.029 | .284 | .594 | .005 | 1.391E-11 | 1648153.167 |
|  | [Pale red tongue =1] | 0^b^ | . | . | . | . | . | . |
|  | [Pale tongue =0] | 9.704 | 27.946 | .121 | .728 | 16384.614 | 2.673E-20 | 10043318449622553000000000000.000 |
|  | [Pale tongue =1] | 0^b^ | . | . | . | . | . | . |
|  | [Red tongue =0] | -20.350 | 9.883 | 4.240 | .039 | 1.452E-9 | 5.615E-18 | .376 |
|  | [Red tongue =1] | 0^b^ | . | . | . | . | . | . |
|  | [Purple Dark Tongue=0] | -9.263 | 9.616 | .928 | .335 | 9.490E-5 | 6.191E-13 | 14547.373 |
|  | [Purple Dark Tongue=1] | 0^b^ | . | . | . | . | . | . |
|  | [Crimson tongue =0] | -15.677 | 11.440 | 1.878 | .171 | 1.554E-7 | 2.842E-17 | 849.647 |
|  | [Crimson tongue =1] | 0^b^ | . | . | . | . | . | . |
|  | [Red tongue tip =0] | 10.118 | 7.287 | 1.928 | .165 | 24785.007 | .016 | 39536261531.398 |
|  | [Red tongue tip =1] | 0^b^ | . | . | . | . | . | . |
|  | [Fat tongue =0] | -24.324 | 9.773 | 6.195 | .013 | 2.731E-11 | 1.312E-19 | .006 |
|  | [Fat tongue =1] | 0^b^ | . | . | . | . | . | . |
|  | [Tooth mark tongue =0] | 2.460 | 2.736 | .808 | .369 | 11.702 | .055 | 2494.184 |
|  | [Tooth mark tongue =1] | 0^b^ | . | . | . | . | . | . |
|  | [Old tongue =0] | -.676 | 2.775 | .059 | .808 | .509 | .002 | 117.013 |
|  | [Old tongue =1] | 0^b^ | . | . | . | . | . | . |
|  | [Tender tongue =0] | -5.591 | 6.040 | .857 | .355 | .004 | 2.698E-8 | 516.310 |
|  | [Tender tongue =1] | 0^b^ | . | . | . | . | . | . |
|  | [Cracked tongue =0] | .687 | 2.982 | .053 | .818 | 1.987 | .006 | 686.280 |
|  | [Cracked tongue =1] | 0^b^ | . | . | . | . | . | . |
|  | [Ecchymosis tongue =0] | .627 | 4.886 | .016 | .898 | 1.872 | .000 | 26977.322 |
|  | [Ecchymosis tongue =1] | 0^b^ | . | . | . | . | . | . |
|  | [Point the tongue =0] | -3.681 | 4.825 | .582 | .446 | .025 | 1.969E-6 | 322.733 |
|  | [Point the tongue =1] | 0^b^ | . | . | . | . | . | . |
|  | [Thin moss =0] | 4.163 | 8.934 | .217 | .641 | 64.293 | 1.599E-6 | 2585487842.992 |
|  | [Thin moss =1] | 0^b^ | . | . | . | . | . | . |
|  | [Moss thickness =0] | .550 | 8.394 | .004 | .948 | 1.733 | 1.240E-7 | 24205515.293 |
|  | [Moss thickness =1] | 0^b^ | . | . | . | . | . | . |
|  | [Tai Ni =0] | 3.218 | 6.233 | .266 | .606 | 24.971 | .000 | 5050302.599 |
|  | [Tai Ni =1] | 0^b^ | . | . | . | . | . | . |
|  | [Moss decay =0] | 9.367 | 14.422 | .422 | .516 | 11694.807 | 6.193E-9 | 22085289317942248.000 |
|  | [Moss decay =1] | 0^b^ | . | . | . | . | . | . |
|  | [Tairun =0] | 8.288 | 9.558 | .752 | .386 | 3977.096 | 2.911E-5 | 543416898889.571 |
|  | [Tairun =1] | 0^b^ | . | . | . | . | . | . |
|  | [Taizao =0] | -1.041 | 9.269 | .013 | .911 | .353 | 4.552E-9 | 27417306.130 |
|  | [Taizao =1] | 0^b^ | . | . | . | . | . | . |
|  | [Slippery tongue =0] | 35.459 | 46.977 | .570 | .450 | 2509503327072258.000 | 2.584E-25 | 2.437E+55 |
|  | [Slippery tongue =1] | 0^b^ | . | . | . | . | . | . |
|  | [Less moss =0] | -11.169 | 8.274 | 1.822 | .177 | 1.410E-5 | 1.277E-12 | 155.755 |
|  | [Less moss =1] | 0^b^ | . | . | . | . | . | . |
|  | [Mirror tongue =0] | 48.578 | 28.973 | 2.811 | .094 | 1250175598124655200000.000 | .000 | 5.744E+45 |
|  | [Mirror tongue =1] | 0^b^ | . | . | . | . | . | . |
|  | [Full tongue coating =0] | -3.315 | 3.475 | .910 | .340 | .036 | 4.001E-5 | 33.007 |
|  | [Full tongue coating =1] | 0^b^ | . | . | . | . | . | . |
|  | [Moss peeling =0] | -15.486 | .000 | . | . | 1.882E-7 | 1.882E-7 | 1.882E-7 |
|  | [Moss peeling =1] | 0^b^ | . | . | . | . | . | . |
|  | [White coating on the tongue =0] | 18.621 | 31.013 | .361 | .548 | 122135062.308 | 4.884E-19 | 30542356498681830000000000000000000.000 |
|  | [White coating on the tongue =1] | 0^b^ | . | . | . | . | . | . |
|  | [Yellow coating on the tongue =0] | 18.667 | 31.172 | .359 | .549 | 127944878.047 | 3.748E-19 | 43682158051002480000000000000000000.000 |
|  | [Yellow coating on the tongue =1] | 0^b^ | . | . | . | . | . | . |
|  | [Yellow and white coating on the tongue =0] | 18.246 | 31.360 | .339 | .561 | 83975362.693 | 1.699E-19 | 41515317571161200000000000000000000.000 |
|  | [Yellow and white coating on the tongue =1] | 0^b^ | . | . | . | . | . | . |
|  | [Grayish-black coating on the tongue =0] | 28.377 | 22.336 | 1.614 | .204 | 2108317533496.575 | 2.049E-7 | 21692956339580890000000000000000.000 |
|  | [Grayish-black coating on the tongue =1] | 0^b^ | . | . | . | . | . | . |
|  | [pulse float =0] | -6.761 | 5.097 | 1.760 | .185 | .001 | 5.316E-8 | 25.230 |
|  | [pulse float =1] | 0^b^ | . | . | . | . | . | . |
|  | [pulse depth =0] | -7.480 | 4.211 | 3.155 | .076 | .001 | 1.469E-7 | 2.168 |
|  | [pulse depth =1] | 0^b^ | . | . | . | . | . | . |
|  | [Pulse slow =0] | -9.506 | 10.409 | .834 | .361 | 7.439E-5 | 1.027E-13 | 53882.582 |
|  | [Pulse slow =1] | 0^b^ | . | . | . | . | . | . |
|  | [Pulse fat =0] | -7.130 | 3.768 | 3.581 | .058 | .001 | 4.974E-7 | 1.290 |
|  | [Pulse fat =1] | 0^b^ | . | . | . | . | . | . |
|  | [ Pulse flood =0] | 46.164 | 368.177 | .016 | .900 | 111902588907150840000.000 | 4.529E-294 | .^c^ |
|  | [Pulse flood =1] | 0^b^ | . | . | . | . | . | . |
|  | [ pulse fineness =0] | -3.157 | 5.414 | .340 | .560 | .043 | 1.047E-6 | 1728.184 |
|  | [ pulse fineness =1] | 0^b^ | . | . | . | . | . | . |
|  | [Weak pulse=0] | -9.972 | 9.579 | 1.084 | .298 | 4.670E-5 | 3.277E-13 | 6656.158 |
|  | [Weak pulse=1] | 0^b^ | . | . | . | . | . | . |
|  | [pulse slippery =0] | .635 | 3.816 | .028 | .868 | 1.888 | .001 | 3342.428 |
|  | [pulse slippery =1] | 0^b^ | . | . | . | . | . | . |
|  | [pulse astringency =0] | 14.086 | 12.028 | 1.371 | .242 | 1311093.422 | 7.570E-5 | 22706696142462356.000 |
|  | [pulse astringency =1] | 0^b^ | . | . | . | . | . | . |
|  | [pulse string =0] | -4.747 | 3.943 | 1.450 | .229 | .009 | 3.826E-6 | 19.699 |
|  | [pulse string =1] | 0^b^ | . | . | . | . | . | . |
|  | [pulse tightness =0] | 8.898 | 22.473 | .157 | .692 | 7314.271 | 5.434E-16 | 98454028579090050000000.000 |
|  | [pulse tightness =1] | 0^b^ | . | . | . | . | . | . |
|  | [pulse knot representation =0] | 2.492 | 6.340 | .154 | .694 | 12.081 | 4.847E-5 | 3010961.476 |
|  | [pulse knot representation =1] | 0^b^ | . | . | . | . | . | . |

**Table S2 Analysis Table of Five Model Training Sets (Baseline + TCM Symptoms)**

| Model | name | precision | recall | f1-score | support |
| --- | --- | --- | --- | --- | --- |
| Logistic | 1 | 0.61 | 0.699 | 0.652 | 103.0 |
|  | 2 | 0.619 | 0.456 | 0.525 | 57.0 |
|  | 3 | 0.504 | 0.629 | 0.56 | 97.0 |
|  | 4 | 0.602 | 0.609 | 0.605 | 92.0 |
|  | 5 | 0.6 | 0.409 | 0.486 | 66.0 |
|  | 6 | 0.5 | 0.472 | 0.486 | 72.0 |
|  | accuracy | 0.567 | 0.567 | 0.567 | 0.567 |
|  | macro avg | 0.573 | 0.546 | 0.552 | 487.0 |
|  | weighted avg | 0.571 | 0.567 | 0.563 | 487.0 |
| XGBoost | 1 | 0.844 | 0.75 | 0.794 | 72.0 |
|  | 2 | 0.782 | 0.835 | 0.808 | 103.0 |
|  | 3 | 0.797 | 0.825 | 0.81 | 57.0 |
|  | 4 | 0.774 | 0.742 | 0.758 | 97.0 |
|  | 5 | 0.769 | 0.761 | 0.765 | 92.0 |
|  | 6 | 0.786 | 0.833 | 0.809 | 66.0 |
|  | accuracy | 0.789 | 0.789 | 0.789 | 0.789 |
|  | macro avg | 0.792 | 0.791 | 0.791 | 487.0 |
|  | weighted avg | 0.789 | 0.789 | 0.788 | 487.0 |
| LGBM | 1 | 0.763 | 0.845 | 0.802 | 103.0 |
|  | 2 | 0.776 | 0.789 | 0.783 | 57.0 |
|  | 3 | 0.811 | 0.794 | 0.802 | 97.0 |
|  | 4 | 0.791 | 0.739 | 0.764 | 92.0 |
|  | 5 | 0.764 | 0.833 | 0.797 | 66.0 |
|  | 6 | 0.823 | 0.708 | 0.761 | 72.0 |
|  | accuracy | 0.786 | 0.786 | 0.786 | 0.786 |
|  | macro avg | 0.788 | 0.785 | 0.785 | 487.0 |
|  | weighted avg | 0.788 | 0.786 | 0.786 | 487.0 |
| Random Forest | 1 | 0.731 | 0.845 | 0.784 | 103.0 |
|  | 2 | 0.82 | 0.719 | 0.766 | 57.0 |
|  | 3 | 0.714 | 0.773 | 0.743 | 97.0 |
|  | 4 | 0.798 | 0.772 | 0.785 | 92.0 |
|  | 5 | 0.812 | 0.788 | 0.8 | 66.0 |
|  | 6 | 0.8 | 0.667 | 0.727 | 72.0 |
|  | accuracy | 0.768 | 0.768 | 0.768 | 0.768 |
|  | macro avg | 0.779 | 0.761 | 0.767 | 487.0 |
|  | weighted avg | 0.772 | 0.768 | 0.768 | 487.0 |
| GBDT | 1 | 0.816 | 0.816 | 0.816 | 103.0 |
|  | 2 | 0.776 | 0.667 | 0.717 | 57.0 |
|  | 3 | 0.747 | 0.763 | 0.755 | 97.0 |
|  | 4 | 0.772 | 0.772 | 0.772 | 92.0 |
|  | 5 | 0.812 | 0.848 | 0.83 | 66.0 |
|  | 6 | 0.76 | 0.792 | 0.776 | 72.0 |
|  | accuracy | 0.78 | 0.78 | 0.78 | 0.78 |
|  | macro avg | 0.78 | 0.776 | 0.777 | 487.0 |
|  | weighted avg | 0.78 | 0.78 | 0.78 | 487.0 |

**TableS3 Analysis Table of Five Model Validation Sets (Baseline + TCM Symptoms)**

| Model | name | precision | recall | f1-score | support |
| --- | --- | --- | --- | --- | --- |
| Logistic | 1 | 0.333 | 0.462 | 0.387 | 13.0 |
|  | 2 | 0.333 | 0.2 | 0.25 | 10.0 |
|  | 3 | 0.333 | 0.312 | 0.323 | 16.0 |
|  | 4 | 0.625 | 0.526 | 0.571 | 19.0 |
|  | 5 | 0.533 | 0.5 | 0.516 | 16.0 |
|  | 6 | 0.353 | 0.462 | 0.4 | 13.0 |
|  | accuracy | 0.425 | 0.425 | 0.425 | 0.425 |
|  | macro avg | 0.419 | 0.41 | 0.408 | 87.0 |
|  | weighted avg | 0.437 | 0.425 | 0.425 | 87.0 |
| XGBoost | 1 | 0.6 | 0.462 | 0.522 | 13.0 |
|  | 2 | 0.364 | 0.308 | 0.333 | 13.0 |
|  | 3 | 0.375 | 0.3 | 0.333 | 10.0 |
|  | 4 | 0.5 | 0.562 | 0.529 | 16.0 |
|  | 5 | 0.5 | 0.474 | 0.486 | 19.0 |
|  | 6 | 0.591 | 0.812 | 0.684 | 16.0 |
|  | accuracy | 0.506 | 0.506 | 0.506 | 0.506 |
|  | macro avg | 0.488 | 0.486 | 0.481 | 87.0 |
|  | weighted avg | 0.497 | 0.506 | 0.496 | 87.0 |
| LGBM | 1 | 0.571 | 0.615 | 0.593 | 13.0 |
|  | 2 | 0.333 | 0.3 | 0.316 | 10.0 |
|  | 3 | 0.462 | 0.375 | 0.414 | 16.0 |
|  | 4 | 0.45 | 0.474 | 0.462 | 19.0 |
|  | 5 | 0.611 | 0.688 | 0.647 | 16.0 |
|  | 6 | 0.538 | 0.538 | 0.538 | 13.0 |
|  | accuracy | 0.506 | 0.506 | 0.506 | 0.506 |
|  | macro avg | 0.494 | 0.498 | 0.495 | 87.0 |
|  | weighted avg | 0.5 | 0.506 | 0.501 | 87.0 |
| Random Forest | 1 | 0.316 | 0.462 | 0.375 | 13.0 |
|  | 2 | 0.444 | 0.4 | 0.421 | 10.0 |
|  | 3 | 0.615 | 0.5 | 0.552 | 16.0 |
|  | 4 | 0.571 | 0.421 | 0.485 | 19.0 |
|  | 5 | 0.429 | 0.562 | 0.486 | 16.0 |
|  | 6 | 0.545 | 0.462 | 0.5 | 13.0 |
|  | accuracy | 0.471 | 0.471 | 0.471 | 0.471 |
|  | macro avg | 0.487 | 0.468 | 0.47 | 87.0 |
|  | weighted avg | 0.497 | 0.471 | 0.476 | 87.0 |
| GBDT | 1 | 0.389 | 0.538 | 0.452 | 13.0 |
|  | 2 | 0.333 | 0.3 | 0.316 | 10.0 |
|  | 3 | 0.533 | 0.5 | 0.516 | 16.0 |
|  | 4 | 0.727 | 0.421 | 0.533 | 19.0 |
|  | 5 | 0.619 | 0.812 | 0.703 | 16.0 |
|  | 6 | 0.462 | 0.462 | 0.462 | 13.0 |
|  | accuracy | 0.517 | 0.517 | 0.517 | 0.517 |
|  | macro avg | 0.511 | 0.506 | 0.497 | 87.0 |
|  | weighted avg | 0.536 | 0.517 | 0.513 | 87.0 |

**Table S4 Analysis Table of Five Model Training Sets (Baseline + TCM Symptoms + Myocardial Injury Markers)**

| Model | name | precision | recall | f1-score | support |
| --- | --- | --- | --- | --- | --- |
| Logistic | 1 | 1.0 | 1.0 | 1.0 | 103.0 |
|  | 2 | 1.0 | 1.0 | 1.0 | 57.0 |
|  | 3 | 1.0 | 1.0 | 1.0 | 95.0 |
|  | 4 | 1.0 | 1.0 | 1.0 | 93.0 |
|  | 5 | 1.0 | 1.0 | 1.0 | 70.0 |
|  | 6 | 1.0 | 1.0 | 1.0 | 69.0 |
|  | accuracy | 1.0 | 1.0 | 1.0 | 1.0 |
|  | macro avg | 1.0 | 1.0 | 1.0 | 487.0 |
|  | weighted avg | 1.0 | 1.0 | 1.0 | 487.0 |
| XGBoost | 1 | 0.954 | 0.935 | 0.944 | 72.0 |
|  | 2 | 0.967 | 0.949 | 0.958 | 103.0 |
|  | 3 | 0.931 | 0.949 | 0.94 | 57.0 |
|  | 4 | 0.948 | 0.948 | 0.948 | 97.0 |
|  | 5 | 0.907 | 0.942 | 0.925 | 92.0 |
|  | 6 | 0.92 | 0.903 | 0.911 | 66.0 |
|  | accuracy | 0.939 | 0.939 | 0.939 | 0.939 |
|  | macro avg | 0.938 | 0.938 | 0.938 | 487.0 |
|  | weighted avg | 0.939 | 0.939 | 0.939 | 487.0 |
| LGBM | 1 | 0.926 | 0.93 | 0.928 | 229.0 |
|  | 2 | 0.931 | 0.949 | 0.94 | 156.0 |
|  | 3 | 0.943 | 0.947 | 0.945 | 263.0 |
|  | 4 | 0.958 | 0.94 | 0.949 | 218.0 |
|  | 5 | 0.958 | 0.975 | 0.966 | 162.0 |
|  | 6 | 0.925 | 0.902 | 0.913 | 163.0 |
|  | accuracy | 0.94 | 0.94 | 0.94 | 0.94 |
|  | macro avg | 0.94 | 0.941 | 0.94 | 487.0 |
|  | weighted avg | 0.94 | 0.94 | 0.94 | 487.0 |
| Random Forest | 1 | 0.942 | 0.961 | 0.951 | 102.0 |
|  | 2 | 0.903 | 0.918 | 0.911 | 61.0 |
|  | 3 | 0.988 | 0.885 | 0.934 | 96.0 |
|  | 4 | 0.896 | 0.966 | 0.93 | 89.0 |
|  | 5 | 0.953 | 0.938 | 0.946 | 65.0 |
|  | 6 | 0.933 | 0.946 | 0.94 | 74.0 |
|  | accuracy | 0.936 | 0.936 | 0.936 | 0.936 |
|  | macro avg | 0.936 | 0.936 | 0.935 | 487.0 |
|  | weighted avg | 0.938 | 0.936 | 0.936 | 487.0 |
| GBDT | 1 | 0.948 | 0.902 | 0.925 | 102.0 |
|  | 2 | 0.906 | 0.951 | 0.928 | 61.0 |
|  | 3 | 0.936 | 0.917 | 0.926 | 96.0 |
|  | 4 | 0.874 | 0.933 | 0.902 | 89.0 |
|  | 5 | 0.938 | 0.923 | 0.93 | 65.0 |
|  | 6 | 0.932 | 0.919 | 0.925 | 74.0 |
|  | accuracy | 0.922 | 0.922 | 0.922 | 0.922 |
|  | macro avg | 0.922 | 0.924 | 0.923 | 487.0 |
|  | weighted avg | 0.923 | 0.922 | 0.922 | 487.0 |

Note:

1 Qi and Yin deficiency syndrome;

2 Qi stagnation and blood stasis syndrome；

3 Phlegm turbidity obstruction syndrome;

4 Syndrome of Yin deficiency of heart and kidney;

5 Syndrome of blood stasis in the heart;

6 Qi deficiency and blood stasis syndrome.

Appendix I: TCM Diagnostic Criteria for Coronary Heart Disease

| Certificate type | clinical picture | Quantitative scoring  （A diagnosis can be made if the total score is ≥8 points.） |
| --- | --- | --- |
| Deficiency syndrome of both Qi and Yin | Chest pain is characterized by chest tightness and dull pain, which is particularly noticeable when subjected to physical exertion. Symptoms include shortness of breath, dry mouth, palpitations, fatigue, dizziness, insomnia, spontaneous sweating and night sweats. The tongue is plump, tender and red with little moisture, and the pulse is fine, weak and feeble | Qi deficiency  1) Chest tightness or chest pain may be triggered by physical exertion (4 points);  2) Fatigue (3 points)  3) Fatigue (3 points);  4) Shortness of breath (3 points)  5) Spontaneous sweating (3 points)  6) Weak pulse (2 points);  7) Pale, plump tongue or with tooth marks (2 points);  8) Palpitations (1 point).  Yin deficiency  1) Dull chest pain (3 points)  2) Hot palms, soles and chest (3 points);  3) Red tongue with little coating (3 points);  4) Night sweats (3 points);  5) Dry mouth (2 points)  6) Insomnia (2 points)  7) Fine pulse (2 points);  8) Dry mouth with no desire to drink (2 points).  A diagnosis can be made if the total score of a single syndrome element is ≥8 points and both qi deficiency and Yin deficiency syndrome elements are met simultaneously. |
| Phlegm blocking the heart pulse syndrome | Chest pain is characterized by chest tightness and pain. Symptoms include excessive phlegm, obesity, dizziness, insomnia, heavy body, fatigue and weakness, and sticky and uncomfortable stools. The tongue coating is thick and greasy, and the pulse is slippery | 1) Chest tightness and pain (3 points)  2) Excessive phlegm and obesity (3 points);  3) Thick and greasy tongue coating (3 points);  4) Sticky stools (2 points);  5) Heavy limbs (2 points);  6) Dizziness and insomnia (2 points);  7) Sticky and uncomfortable mouth (2 points);  8) Slippery pulse (2 points). |
| Syndrome of Yin deficiency of the heart and kidneys | Chest pain is characterized by intermittent pain. Symptoms include soreness and weakness in the waist and knees, palpitations, insomnia, hot flushes in the palms, soles and chest, dry mouth and throat, night sweats, red tongue with little coating, and fine and rapid pulse | 1) Dull chest pain (3 points)  2) Hot palms, soles and chest (3 points);  3) Red tongue with little coating (3 points);  4) Night sweats (3 points);  5) Day dry (2 points);  6) Insomnia (2 points)  7) Fine pulse (2 points);  8) Dry mouth with no desire to drink (2 points). |
| Syndrome of Qi stagnation and blood stasis | Chest pain is characterized by chest tightness and distension, often triggered by emotional distress. Symptoms include excessive breathing, distension and oppression in the epigastrium, abdomen and hypochondrium, and relief when belching or flatulence occurs. The tongue is purple or dark red, and the pulse is taut | Qi stagnation  1)Chest tightness, distension and pain (mostly induced by emotions)(3 points);  2) Impatient and irritable (3 points);  3) Costal distension or pain (3 points);  4) Epigastric distension (3 points)  5) Heating (2 points);  6) Bitter taste in the mouth (2 points)  7) Dark red tongue (2 points);  8) Taut pulse (2 points).  Blood stasis  1) Fixed chest pain (4 points);  2) The tongue is dark purple or has ecchymosis and petechiae on the tongue body (4 points);  3) The sublingual vein is dark purple (3 points);  4) Dark purple complexion (3 points);  5) There are petechiae or ecchymosis on the body (3 points);  6) Numbness of limbs (2 points);  7) Dark purple or dark red lips (2 points):  8) Sluggish pulse (2 points).  A diagnosis can be made if the total score of a single syndrome element is ≥8 points and both qi stagnation and blood stasis syndrome elements are met simultaneously. |
| Syndrome of blood stasis in the heart | Chest pain is characterized by fixed pain. Symptoms include a dark purple complexion, numbness in limbs, dark purple or dark red lips, a dark red or dark purple tongue with petechiae and ecchymosis on the tongue body, dark purple veins under the tongue, and a sluggish or connective pulse | 1) Fixed chest pain (4 points);  2) The tongue is dark purple or has ecchymosis and petechiae on the tongue body (4 points);  3) The sublingual vein is dark purple (3 points);  4) Dark purple complexion (3 points);  5) There are petechiae or ecchymosis on the body (3 points);  6) Numbness of limbs (2 points);  7) Lips are dark purple or dark red (2 points);  8) Sluggish pulse (2 points). |
| Qi deficiency and blood stasis syndrome | Chest pain is characterized by chest tightness and pain, which is triggered by physical exertion. Symptoms include shortness of breath, fatigue, listlessness, reluctance to speak, palpitations, spontaneous sweating, pale or dull complexion, a plump and pale tongue, and a deep and sluggish pulse | Qi deficiency :  1) Chest tightness or chest pain is triggered by physical exertion (4 points);  2) Fatigue (3 points)  3) The force (3 points);  4) Shortness of breath (3 points)  5) Spontaneous sweating (3 points)  6) Weak pulse (2 points);  7) Pale, plump tongue or with tooth marks (2 points);  8) Palpitations (1 point).  Blood stasis :  1) Fixed chest pain (4 points);  2) The tongue is dark purple or has ecchymosis and petechiae on the tongue body (4 points);  3) The sublingual vein is dark purple (3 points);  4) Dark purple complexion (3 points);  5) There are petechiae or ecchymosis on the body (3 points);  6) Numbness of limbs (2 points);  7) Lips are dark purple or dark red (2 points);  8) Sluggish pulse (2 points).  A diagnosis can be made if the total score of a single syndrome element is ≥8 points and both qi deficiency and blood stasis syndrome elements are met simultaneously. |
| Syndrome of deficiency and decline of Yang Qi | Chest pain is characterized by chest tightness and pain, which worsens when exposed to cold. Symptoms include aversion to cold, cold limbs, palpitations, spontaneous sweating, fatigue, pale complexion, loose stools, swollen limbs, pale and plump tongue with white coating, and deep and slow pulse. | 1) Chest tightness or pain (4 points);  2) Cold limbs and fear of cold (3 points);  3) Shortness of breath when moving (3 points);  4) Loose stools (2 points);  5) Frequent urination at night (2 points)  6) Coldness in the epigastrium, abdomen and waist (2 points);  7) Pale, plump and moist tongue (2 points);  8) Deep pulse (2 points). |
| Syndrome of cold and dampness stagnation | Chest pain is characterized by a sudden sharp pain that feels like a throbbing sensation, and cold pain is particularly prominent. Symptoms include coldness in the body, cold limbs, spontaneous cold sweats, pale complexion, palpitations, shortness of breath, thin white coating on the tongue, and a deep and tight pulse | 1)Chest pain occurs when exposed to cold (4 points);  2) Cold and cramped limbs (3 points);  3) Cold lumbosacral region (3 points);  4) Cold pain in the abdomen (3 points)  5) The tongue is dark blue or purple (3 points);  6) Pale complexion (2 points);  7) Pale complexion (2 points);  8) Deep or slow pulse (2 points). |
